# Toward disease-associated fecal microbiome signatures for domestic mammals

**DOI:** 10.1038/s42003-026-10647-5

**Published:** 2026-07-14

**Authors:** Dan Lin, Erika Ganda, Justin Silverman, Jiating Huang, Katherine Rose Gottwald, Haowen Hu, Puchun Niu, Jessica Anne Allerton McArt

**Affiliations:** 1 Department of Population Medicine and Diagnostic Sciences, College of Veterinary Medicine, Cornell University, Ithaca, NY USA; 2 Department of Animal Science and One Health Microbiome Center, Pennsylvania State University, University Park, PA USA; 3 College of Information Sciences and Technology, Pennsylvania State University, University Park, PA USA; 4 Department of Statistics, Pennsylvania State University, University Park, PA USA; 5 Department of Medicine, Pennsylvania State University, Hershey, PA USA; 6 Department of Epidemiology and Health Statistics, Zhejiang University School of Public Health, Hangzhou, China; 7 Department of Animal Science, College of Agriculture and Life Sciences, Cornell University, Ithaca, NY USA

**Keywords:** Microbiome, Diagnostic markers

## Abstract

Conserved fecal microbiome signatures of intestinal diseases across domestic mammals offer a non-invasive avenue to monitor animal health and advance One Health, yet systematic meta-analysis with cross-study, cross-disease, and cross-host validation remains lacking. We present a leave-one-dataset-out meta-analysis defining generalizable intestinal disease signatures in mammalian hosts. Analyzing 512 samples from four bovine diarrhea and three canine IBD studies, we identified 12 bovine and 8 canine stable signature genera marked by depleted short-chain fatty acid (SCFA) producers and enriched pathobionts through a compositional random-effects framework with per-study covariate-adjusted effect sizes. A minimal signature set matched full-feature models across five machine learning models, and study-aware transfer learning improved cross-study generalization. Across 367 samples, signatures outperformed for intestinal versus non-intestinal phenotypes in cross-disease prediction. Cross-host validation across equine, feline, caprine, swine, and human datasets (533 animal and 1,182 human samples) revealed canine IBD-associated signatures outperformed in feline intestinal disease prediction, while bovine diarrhea-associated signatures generalized better to herbivorous hosts and human intestinal diseases. These findings may support the existence of conserved microbial signatures across veterinary and human medicine, warranting further investigation toward diagnostic applications.

## Abstract (web summary)

In this study, Lin et al. proposed the identification of conserved fecal microbiome signatures of intestinal diseases for domestic mammals. The meta-analysis identified microbial signatures associated with depleted short-chain fatty acid producers and enriched pathobionts in bovine and canine diseases. The cross-disease and cross-host validation also revealed that the signatures were conserved and generalized across domestic mammals and may serve as microbial biomarkers for the early detection of intestinal diseases in domestic mammals.

## Introduction

As in humans, mammalian gastrointestinal tracts are complex ecosystems with large and diverse microbial communities. These gut microbes perform essential functions for their hosts, contributing not only to local processes such as food digestion, nutrient absorption, gut development, and pathogen defense, but also to systemic metabolic homeostasis through interorgan communication pathways, like the gut-brain^1^, gut-liver^2^, gut-breast^3^ and gut-skin^4^ axes. Disruptions to these microbial ecosystems, known as dysbiosis, are associated with many diseases in domestic mammals, including subacute ruminal acidosis^5^ and mastitis^6^ in dairy cows, colic in horses^7^, Inflammatory Bowel Disease (IBD) in dogs^8^, and diabetes in cats^9^. Gut microbial dysbiosis can even promote the selection of more virulent microbial strains and further adversely affect host health, like calf diarrhea^10^ and sheep gastrointestinal nematode infection^11^. These disease-gut microbiome associations further spurred research into shared microbial signatures across common diseases. In humans, common fecal microbiome alterations have been reported in a wide array of diseases, ranging from colorectal cancer and IBD to metabolic and cardiovascular conditions^12,13^. In mouse models, two distinct cardiovascular diseases have been shown to involve shared gut microbial and metabolic alterations^14^. These works provide confidence to identify reproducible gut microbiome patterns across diseases within humans and mice. Extending this exploration to domestic mammals can inform the development of clinically useful diagnostic and therapeutic approaches for diverse diseases.

Furthermore, comparing gut microbial signatures of common diseases across mammalian species can expand our understanding of gut microbial dysbiosis with diverse genetic backgrounds, and open doors for developing universal microbiome-based strategies for their health management. ‘Phylosymbiosis’^15^ has revealed certain gut microbial taxa can be unexpectedly prevalent across diverse wild mammalian species^16^, including even-toed ungulates on isolated regions^17^, mammalian herbivores^18,19^, primates^20,21^, and small mammals (mice, voles, shrews)^22^, regardless of whether they share habitats or diets. These studies have provided biological evidence to support the shared host-microbiome association patterns. To explore shared gut microbiome patterns across multiple diseases and mammal hosts, domestic mammals offer a more suitable model system than wild mammals, due to their well-controlled external environments and well-documented health records. Moreover, these domestic mammals share environments, exposures, and treatments with humans^23^, offering more relevant models for microbiome–disease studies than standardized, experimentally induced lab mice. However, such investigations have not been systematically conducted to date.

**Fig. 1:**
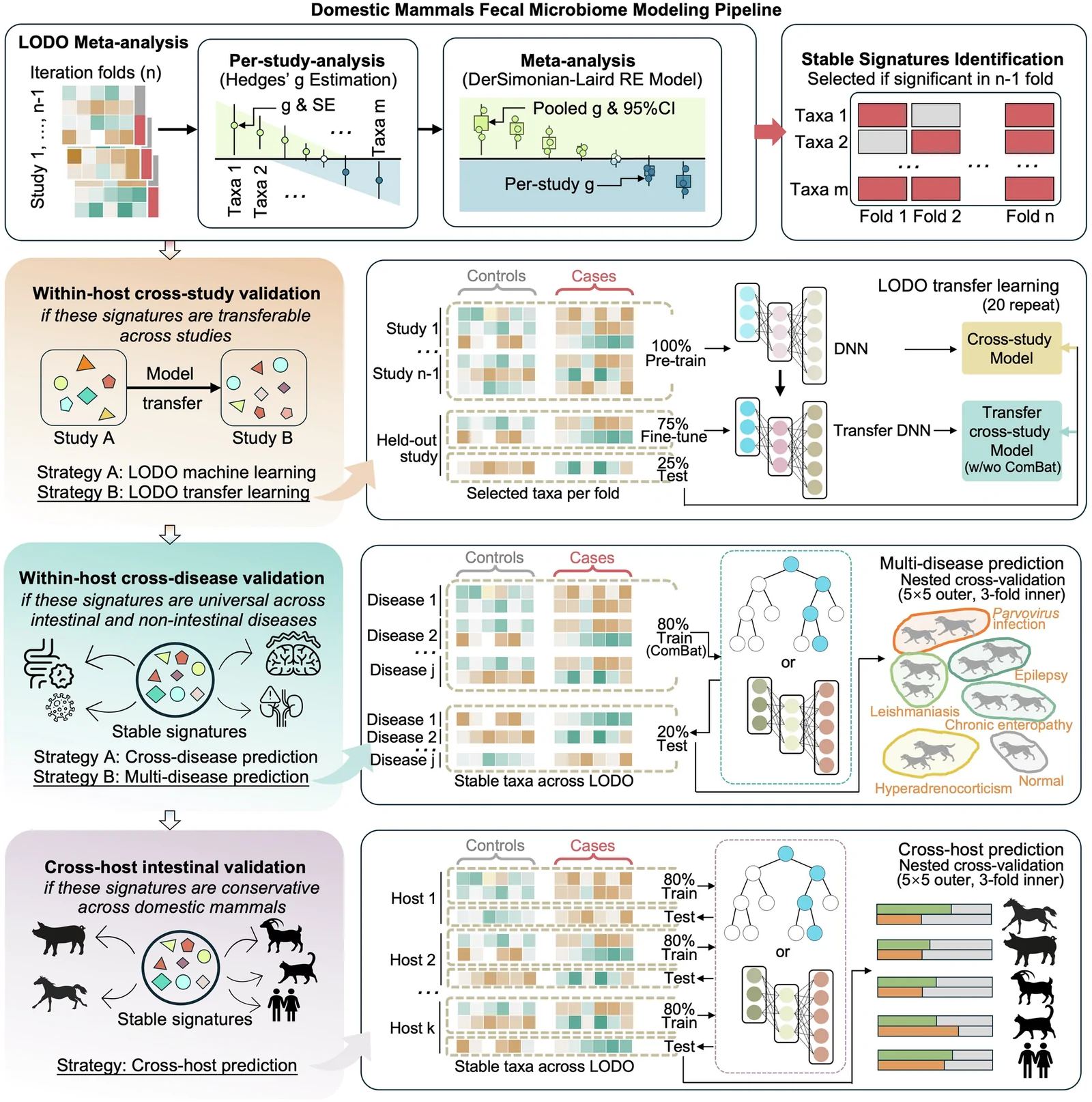
Predictive modeling workflow using publicly available fecal microbiome databases of domestic mammals under a case–control design. The framework encompasses four analytical tiers: (1) deriving stable disease-associated microbial signatures via leave-one-dataset-out (LODO) nested meta-analysis; (2) assessing cross-study reproducibility through LODO machine learning and deep neural network (DNN) transfer learning; (3) evaluating cross-disease specificity by applying signatures to independent intestinal and non-intestinal disease datasets within the same host; and (4) testing cross-host generalizability by applying bovine and canine signatures to other domestic mammal and human gut microbiome datasets. Disease icons and animal and human silhouettes were obtained from Freepik (https://www.freepik.com) under a free license with attribution.

**Fig. 2:**
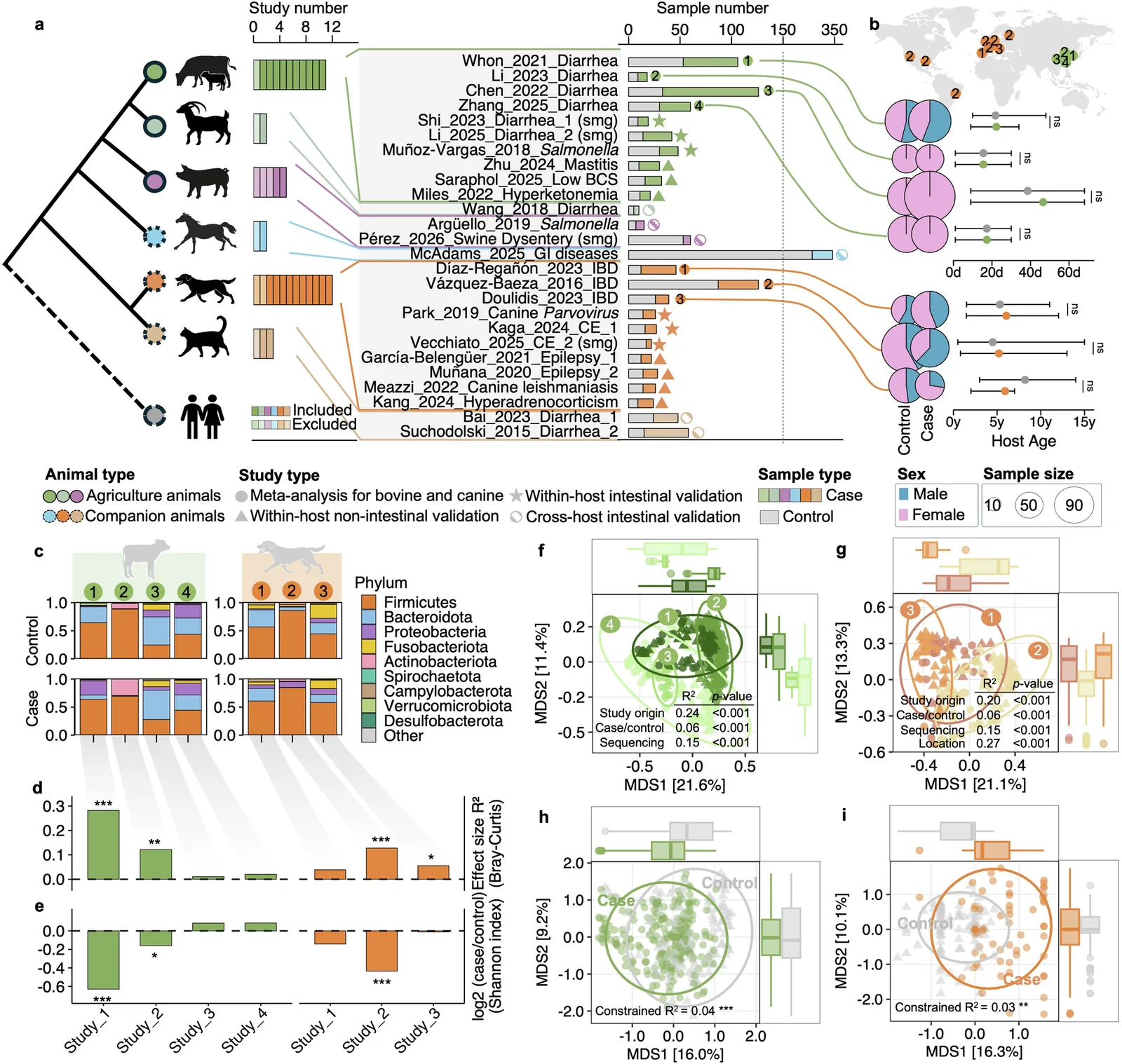
Dataset overview. **a** Overview of all fecal microbiome case–control datasets included in this study, organized by host species phylogeny. A total of 26 studies across 6 domesticated mammals (803 controls and 618 cases) were retrieved from public databases. Studies used for bovine and canine meta-analysis are numbered (1–4 and 1–3, respectively); remaining datasets served as within-host intestinal, within-host non-intestinal, or cross-host intestinal validation. Green and orange circles on the phylogenetic tree highlight the two primary hosts (bovine and canine). Study types are indicated by shape markers: filled circles for meta-analysis, stars for within-host intestinal validation, filled triangles for within-host non-intestinal validation, and hatched circles for cross-host intestinal validation. In the sample bar chart, colored and gray segments represent case and control samples, respectively, with bar colors corresponding to host species. The phylogenetic tree topology was retrieved from O’Leary et al.^91^. **b** Sampling sites, sex and host age distributions for the four bovine diarrhea and three canine inflammatory bowel disease (IBD) meta-analysis studies. Sex composition is shown as pie charts (pink for female, blue for male), with circle size proportional to sample size. **c** Mean phylum-level relative abundance of fecal microbiota in case and control groups across the four bovine and three canine meta-analysis studies. **d** Effect size (R²) of case–control status on genus-level microbiome composition in each meta-analysis study, assessed by permutational multivariate analysis of variance (PERMANOVA) on Bray–Curtis distances (999 permutations; *p < 0.05, **p < 0.01, ***p < 0.001). Green and orange bars represent bovine and canine studies, respectively. **e** Log₂ fold change (case/control) in fecal microbial alpha diversity (Shannon index) for each meta-analysis study, assessed by Wilcoxon rank-sum test (*p < 0.05, ***p < 0.001). Metric multidimensional scaling (MDS) of Bray–Curtis distances for the four bovine (**f**) and three canine (**g**) meta-analysis studies. Each study is assigned a distinct color and represented by a 95% confidence ellipse; boxplots depict sample distributions along each axis. Inset PERMANOVA R² values quantify the contributions of study origin, case–control status, sequencing platform, and geographic location to microbiome variation. MDS plots after removing the effects of study origin, sequencing platform, and geographic location via distance-based redundancy analysis (dbRDA), revealing residual case–control separation in bovine (**h**) and canine (**i**) cohorts. Case and control groups are shown in green and gray (bovine, **h**) or orange and gray (canine, **i**), each represented by a 95% confidence ellipse; boxplots depict sample distributions along each axis. In all boxplots, the center line indicates the median, box limits represent the 25th and 75th percentiles, and whiskers extend to 1.5× the interquartile range. Sample sizes (*n* biologically independent samples): bovine diarrhea meta-analysis: Study_1, *n* = 106 (53 controls, 53 cases); Study_2, *n* = 18 (9 controls, 9 cases); Study_3, *n* = 126 (33 controls, 93 cases); Study_4, *n* = 60 (30 controls, 30 cases); canine IBD meta-analysis: Study_1, *n* = 46 (12 controls, 34 cases); Study_2, *n* = 126 (87 controls, 39 cases); Study_3, *n* = 39 (26 controls, 13 cases). Animal and human silhouettes were obtained from Freepik (https://www.freepik.com).

Given the limited sample sizes, high heterogeneity, technical variability, and batch effects in existing case–control microbiome studies of domestic mammals (Supplementary Data 1), pooling all data for joint analysis and modeling remains challenging. To address this, we first conducted two meta-analyses on 521 fecal 16S samples from four bovine (calf) diarrhea and three canine IBD studies to define genus-level microbial signatures of intestinal disease. These two host-disease combinations were selected based on the availability of multiple comparable case–control 16S rRNA studies (Supplementary Data 1, 2 and Figs. 1 and 2a), their clinical importance as prevalent intestinal conditions in livestock and companion animals, respectively^24,25^, and their contrasting digestive physiologies as a ruminant herbivore and a monogastric companion animal^26^, which together facilitate exploration of both host-specific and conserved dysbiosis patterns. We then assessed their generalizability using a suite of modeling strategies (Fig. 1) across an additional 900 samples spanning bovine and canine intestinal and non-intestinal conditions, as well as caprine, swine, equine, and feline intestinal diseases, and further evaluated their predictive relevance in humans using seven MicrobiomeHD datasets^27^ totaling 1182 samples across three intestinal conditions. While not representing the entire gut, the fecal microbiome used here serves as a practical and widely used proxy due to its non-invasive accessibility and relevance to host health in large-scale animal studies^28^. Our goal is to explore whether consistent, generalizable microbial signatures exist across studies, multiple diseases and domestic mammalian hosts, potentially informing lightweight diagnostic tools in veterinary contexts and supporting cross-host insight into gut dysbiosis under the One Health framework.

## Results

### Meta-analysis dataset overview

We analyzed a total of 310 samples (185 controls and 125 cases) from four bovine (calf) diarrhea case–control studies^10,29–31^ conducted in two Asian countries, and 211 samples (125 controls and 86 cases) from three canine IBD case–control studies^8,23,32^ across several European and American countries (Fig. 2b). All datasets were profiled using 16S rRNA amplicon sequencing on the Illumina MiSeq, HiSeq, or NovaSeq platforms (Supplementary Data 2). Although the overall sample size remains modest, this work represents, to the best of our knowledge, the first meta-analysis of diarrhea and IBD in domestic mammals. Within each individual study, case–control groups were relatively balanced in sex and age composition (all *p* > 0.05 by Fisher’s Exact test for sex and Wilcoxon rank sum test for age; Fig. 2b), with the exception of canine IBD Study_2 where sex distribution differed (*p* = 0.04), which may reduce potential confounders in the subsequent meta-analysis.

To ensure our unified pipeline produces biologically consistent results with these prior studies, we first investigated whether reported core fecal microbiome in each host would be recapitulated. In both hosts, *Firmicutes* and *Bacteroidota* were consistently the most abundant phyla across both controls and cases (bovine controls: 24.7–88.6% and 13.0–50.0%; canine controls: 44.8–86.2% and 6.00–30.7%; bovine cases: 28.0–69.4% and 12.2–52.3%; canine cases: 58.1–84.7% and 16.6–22.5%; Fig. 2c). At the genus level, bovine controls were characterized by *Bacteroides* (18.8 ± 14.4%; mean ± SD of relative abundance across all samples), *Faecalibacterium* (7.60 ± 11.6%), and *Alloprevotella* (3.53 ± 6.66%), whereas cases were dominated by *Bacteroides* (18.2 ± 14.9%), *Escherichia–Shigella* (11.7 ± 17.4%), and *Faecalibacterium* (6.14 ± 9.36%). In canine samples, controls were dominated by *Peptoclostridium* (30.8 ± 20.4%), *Fusobacterium* (6.37 ± 13.4%), and *Bacteroides* (6.26 ± 10.4%), while cases were characterized by *Streptococcus* (14.5 ± 22.6%), *Peptoclostridium* (13.4 ± 21.9%), and *Bacteroides* (9.47 ± 14.8%) (Supplementary Fig. 1a). These results were consistent with original study findings^8,10,23,29–32^, and indicated the distinct genus-level gut microbiota profiles between diarrhea/IBD cases and healthy controls within each host, though inter-study variability was observed.

We next confirmed that our unified pipeline could recapitulate previously reported associations between the fecal microbiome and diarrhea/IBD within each host. Consistently, all studies reproduced their original findings, with most datasets (2/4 in calf diarrhea and 2/3 in canine IBD) showing that community composition was significantly associated with disease status (PERMANOVA test on Bray–Curtis distance, *p* < 0.05, Fig. 2d), further supported by weighted UniFrac and Jaccard metrics (Supplementary Fig. 1b). Moreover, case groups consistently exhibited significant reductions in taxon richness, evenness, and phylogenetic diversity relative to controls, as estimated by Shannon diversity (Fig. 2e), Chao1, and Faith’s PD (Supplementary Fig. 1b), with 2/4 bovine and 1/3 canine studies reaching statistical significance (Wilcoxon rank sum test, *p* < 0.05). These results further indicated an altered gut microbiome in these mammals with intestinal diseases.

When combining all samples from these case–control studies within each host, PERMANOVA indicated that although disease status was statistically significant (on Bray–Curtis distance, *p* < 0.001), it only explained 6% of the variation in fecal microbial composition (Fig. 2f, g). In contrast, study origin and sequencing platform explained 24% and 15% of variance in bovine, and 20% and 27% in canine datasets, respectively. Both values were substantially higher than the contribution of disease status. Even after adjusting for study origin and sequencing platform effects using Distance-based redundancy analysis (dbRDA, on Bray–Curtis distance), disease status explained only 4% and 3% of the residual variance in fecal microbial composition (bovine: constrained R² = 4%, F = 15.9, *p* < 0.001; canine: constrained R² = 3%, F = 3.46, *p* = 0.004; Fig. 2h, i). These findings demonstrated substantial inter-study variability in gut microbiome composition within the same host and disease, a common feature of microbiome meta-analyses^33–35^.

### Gut dysbiosis featured a shared depletion of beneficial taxa alongside host-specific enrichment patterns

**Fig. 3:**
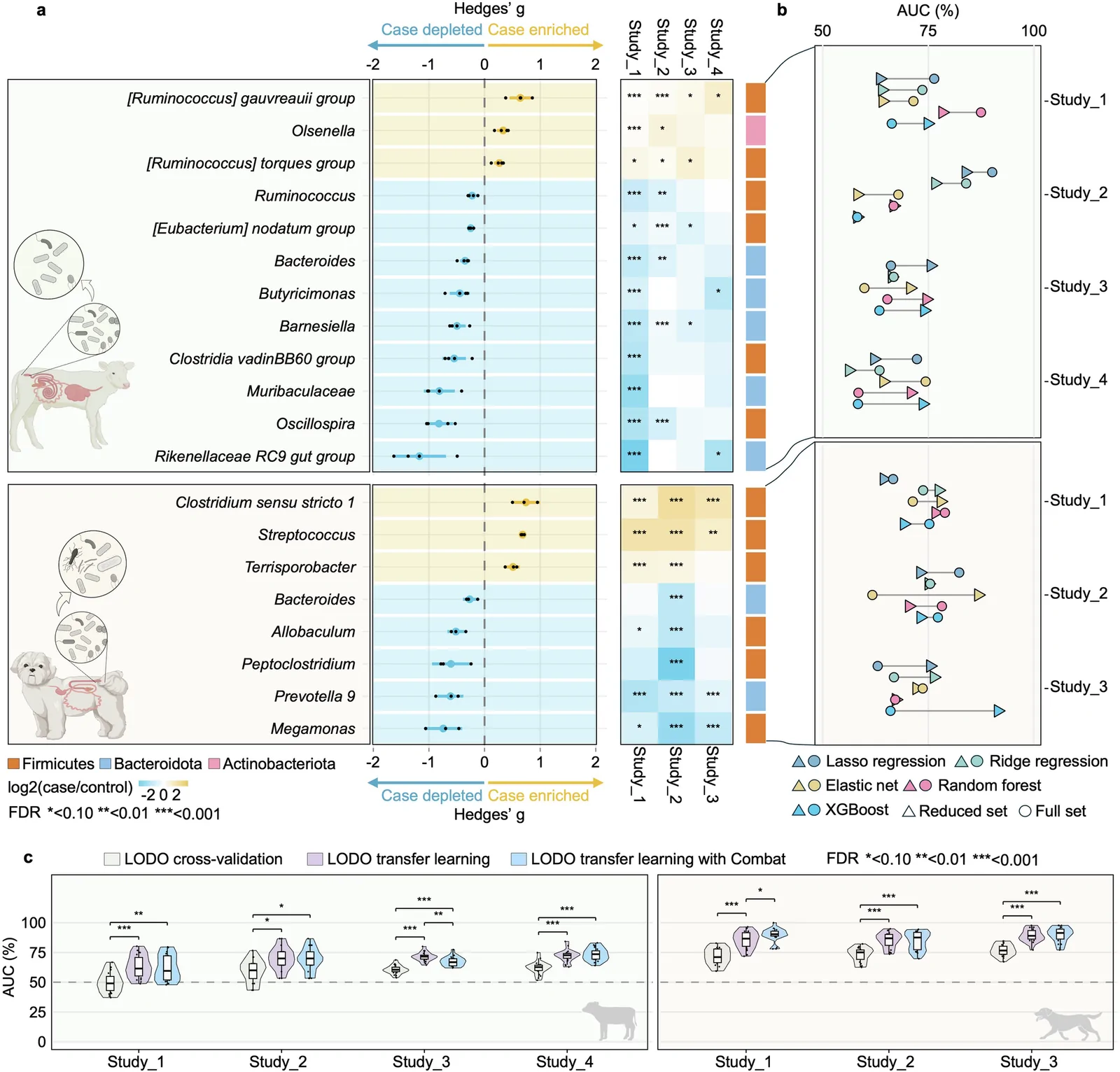
Meta-analysis-derived disease signatures for bovine diarrhea and canine IBD, and within-host cross-study validation. **a** Leave-one-dataset-out (LODO) nested meta-analysis results for bovine diarrhea (4 studies) and canine IBD (3 studies). Forest plots show the pooled Hedges’ g effect size across folds (median, large dot; per-study g, small dots) with 95% confidence interval (CI) for each signature genus; yellow and blue backgrounds indicate case enrichment and depletion, respectively. The heatmap color reflects the log₂(case/control) abundance ratio. Colored squares next to each genus indicate phylum membership (orange for *Firmicutes*, blue for *Bacteroidota*, pink for *Actinobacteriota*). Per-study false discovery rate (FDR)-corrected significance is indicated (*p < 0.10, **p < 0.01, ***p < 0.001). Illustrations of the calf, dog, gastrointestinal tract, and microbiome were created with BioRender (Lin, D. (2026) https://BioRender.com/qk42h33). **b** Within-host cross-study classification performance (area under the curve, AUC) of five machine learning classifiers (LASSO, Ridge, Elastic Net, Random Forest, XGBoost) trained on signature genera (reduced set, triangles) or all genera (full set, circles) under the LODO nested cross-validation framework, evaluated on each held-out study for bovine and canine. Each classifier is color-coded: blue for LASSO, green for Ridge, yellow for Elastic Net, pink for Random Forest, and teal for XGBoost; triangles denote the reduced (signature) set and circles denote the full set. **c** Comparison of three cross-study transfer strategies (LODO cross-validation, LODO transfer learning, and LODO transfer learning with ComBat batch correction) for bovine and canine. AUC distributions across 20 repeated runs are shown as color-coded violin plots with embedded boxplots (white for LODO cross-validation, purple for LODO transfer learning, and blue for LODO transfer learning with ComBat), with individual repeat values overlaid as points. FDR-corrected pairwise comparisons: *p < 0.10, **p < 0.01, ***p < 0.001. In all boxplots, the center line indicates the median, box limits represent the 25th and 75th percentiles, and whiskers extend to 1.5× the interquartile range. Sample sizes (*n* biologically independent samples): bovine diarrhea meta-analysis: Study_1, *n* = 106 (53 controls, 53 cases); Study_2, *n* = 18 (9 controls, 9 cases); Study_3, *n* = 126 (33 controls, 93 cases); Study_4, *n* = 60 (30 controls, 30 cases); canine IBD meta-analysis: Study_1, *n* = 46 (12 controls, 34 cases); Study_2, *n* = 126 (87 controls, 39 cases); Study_3, *n* = 39 (26 controls, 13 cases). Animal and human silhouettes were obtained from Freepik (https://www.freepik.com).

The Leave-One-Dataset-Out (LODO)-nested meta-analysis identified 12 bovine and 8 canine stable taxa consistently altered across studies (Fig. 3a; mean taxa retention across folds: 83.3% bovine, 91.7% canine; each fold’s results in Supplementary Table 1 and Supplementary Table 2), corresponding to all taxa significant in the full pooled meta-analysis for bovine and eight of nine for canine (Supplementary Fig. 2), and robust across CLR-based zero-handling parameterizations and FDR thresholds of 0.05–0.20 (Supplementary Fig. 3). In both hosts, the common depletion of *Bacteroidota* commensals, notably *Bacteroides* (bovine: median Hedges’ g across folds = −0.35 [95% CI −0.44 to −0.26]; canine: g = −0.27 [−0.39 to −0.15]), along with *Rikenellaceae RC9 gut group* (g = −1.17 [−1.65 to −0.69]), *Barnesiella* (g = −0.50 [−0.65 to −0.34]) and *Muribaculaceae* (family-level) (g = −0.81 [−1.09 to −0.53]) in bovine, and *Prevotella 9* (g = −0.61 [−0.85 to −0.38]) and *Megamonas* (g = −0.74 [−1.08 to −0.40]) in canine, points to a compromised capacity for complex polysaccharide degradation^36,37^. This was accompanied by a broad loss of short-chain fatty acid (SCFA)-producing taxa in both hosts, particularly *Oscillospira*^38^ (g = −0.82 [−1.07 to −0.57]) and *Ruminococcus*^38^ (g = −0.22 [−0.30 to −0.15]), as well as *Butyricimonas*^39^ (g = −0.45 [−0.63 to −0.27]) in bovine, and *Allobaculum*^40^ (g = −0.52 [−0.67 to −0.36]) and *Peptoclostridium*^40^ (g = −0.61 [−0.95 to −0.27]) in canine, reflecting a shared collapse of beneficial microbial communities.

Distinct enrichment patterns characterized the gut microbial dysbiosis in each host-specific intestinal disease. Canine IBD was commonly marked by the proliferation of potential pathobionts including *Streptococcus*^8,41^ (g = +0.68 [+0.65 to +0.72]), *Clostridium sensu stricto 1*^8^ (g = +0.74 [+0.49 to +1.00]), and *Terrisporobacter*^42^ (g = +0.51 [+0.39 to +0.63]), indicating a shift from passive commensal loss toward active pathobiont expansion, suggestive of immune containment failure. In contrast, bovine diarrhea was enriched in *[Ruminococcus] torques group*^43^ (g = +0.27 [+0.17 to +0.36]) and *Olsenella*^44^ (g = +0.33 [+0.22 to +0.44]), suggesting concurrent mucosal barrier erosion and lactate-driven suppression of commensal recovery. These findings reveal a conserved pattern of commensal depletion across both hosts, overlaid with host-specific compositional shifts pointing to divergent pathobiological mechanisms, though broader validation remains warranted.

### The identified microbes generalize robustly across datasets with markedly reduced feature sets

To evaluate the cross-dataset generalizability of the identified signatures, we applied a LODO machine learning framework across all folds (Figs. 1 and 3b; sensitivity and specificity in Supplementary Fig. 4). The per-fold signature sets comprised a small fraction of the full feature space (bovine diarrhea: 20–39 (9–18%) of 214 genera; canine IBD: 13 (13%) of 98 genera; Supplementary Table 1). Despite this compactness, prediction performance on the held-out study was comparable to models trained on the full feature set (four bovine diarrhea studies: 69.0%, 68.7%, 72.2%, 65.5% vs. 75.1%, 73.4%, 64.3%, 65.4%; three canine IBD studies: 73.2%, 75.7%, 76.6% vs. 73.2%, 75.0%, 67.4%; mean area under the receiver operating characteristic curve (AUC) across five classifiers), indicating the identified taxa capture the majority of the disease-discriminative signal. Feature importance scores within full-feature models further supported this, with signature genera consistently assigning higher importance than non-signature genera across studies and classifiers (Wilcoxon signed-rank test, *p* < 0.05; Supplementary Fig. 5). Hyperparameter sensitivity analysis across LODO folds showed that prediction performance was largely insensitive to the choice of hyperparameter values across all five classifiers (Supplementary Fig. 6), suggesting that the observed performance reflects genuine signal rather than model-tuning artifacts. These results demonstrate that the identified signatures capture generalizable diagnostic signals that remain robust across machine learning classifiers and hyperparameter settings, even across independent studies of the same host-disease combination despite considerable inter-study heterogeneity (Fig. 2c–i).

### Transfer learning further improves cross-dataset adaptability of the identified microbes

To evaluate the benefit of local fine-tuning for cross-study generalization, we implemented a LODO transfer learning framework (Fig. 1). Transfer learning consistently outperformed direct LODO cross-study prediction across both hosts (four bovine diarrhea studies: 49.9%, 59.2%, 60.6%, and 62.0% AUC for LODO cross-study vs. 63.2%, 70.0%, 71.5%, and 72.1% for LODO transfer learning; three canine IBD studies: 71.7%, 73.1%, and 76.9% vs. 85.4%, 85.3%, and 89.3%; all FDR-corrected *p* < 0.10; Fig. 3c; sensitivity and specificity in Supplementary Fig. 7a), confirming that target-domain fine-tuning benefits generalization.

To further characterize factors influencing transfer learning performance, we examined the effects of batch correction and network depth. Applying ComBat to the training data within each fold prior to fine-tuning offered no improvement over transfer learning alone (though the combination still outperformed direct cross-study prediction; FDR-corrected *p* < 0.10 in 6/7 studies; Fig. 3c). Network depth, however, did prove important, with medium and deep configurations consistently outperforming shallow networks (FDR-corrected *p* < 0.10 in 6/7 studies; Supplementary Fig. 7b), indicating that sufficient model capacity is required to capture cross-study disease signals.

### The identified microbes may help predict other intestinal diseases within the same host

**Fig. 4:**
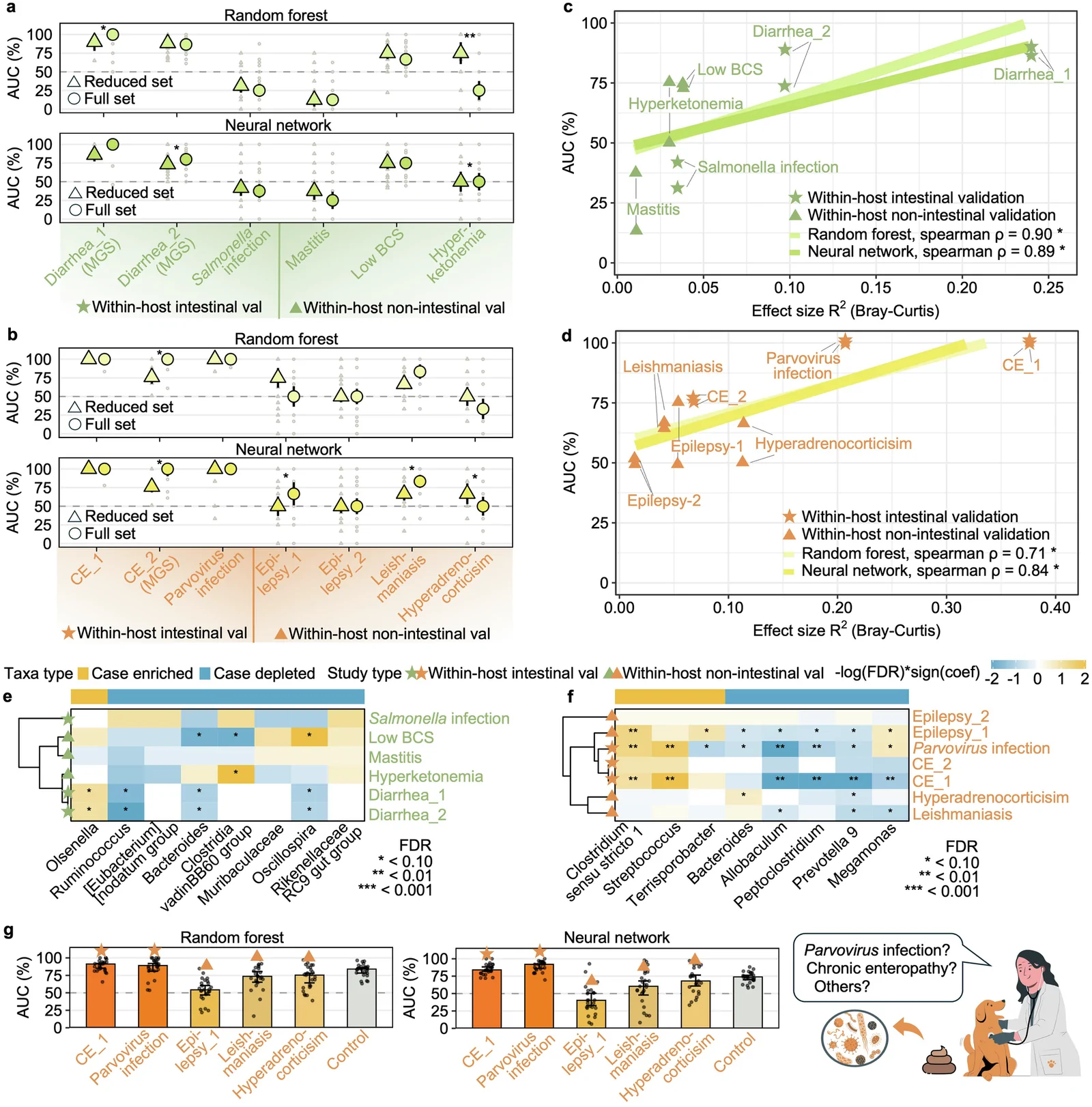
Within-host cross-disease validation of bovine diarrhea and canine IBD- associated signatures. Binary classification performance (AUC) of Random Forest and Neural Network models using bovine diarrhea-associated (**a**) or canine IBD-associated (**b**) stable signature genera (reduced set, triangles) or all genera (full set, circles), evaluated across within-host intestinal (stars) and non-intestinal (triangles) validation datasets via 5 repeats × 5-fold cross-validation. Individual repeat values are shown as gray points. FDR-corrected significance: *p < 0.10, **p < 0.01, ***p < 0.001. Spearman correlation between the PERMANOVA effect size (R², Bray–Curtis distances, 999 permutations) of signature genera and classification AUC across bovine (**c**) and canine (**d**) validation datasets, for Random Forest (lighter color) and Neural Network (darker color). Intestinal validation datasets are shown as star markers and non-intestinal datasets as triangle markers. MaAsLin2 association heatmaps showing the relationship between bovine diarrhea (**e**) or canine IBD (**f**) signature genera and disease status across all within-host validation datasets. Color reflects –log(FDR) × sign(coefficient), indicating direction and significance of association; asterisks denote FDR-corrected significance (*p < 0.10, **p < 0.01, ***p < 0.001). Row annotations indicate taxa type (yellow for case-enriched, blue for case-depleted) and study type (star for within-host intestinal validation, triangle for within-host non-intestinal validation). **g**. Multi-class disease prediction performance (AUC per class) in canine, using the IBD signature genera to simultaneously distinguish six classes (chronic enteropathy [CE], *parvovirus* infection, epilepsy, leishmaniasis, hyperadrenocorticism, and normal), evaluated by Random Forest and Neural Network with 5-fold stratified cross-validation × 5 repeats. Individual fold values are overlaid as points. The veterinarian and dog illustration was created with Storyset (https://storyset.com/illustration/veterinary/pana). Error bars indicate 95% confidence intervals across cross-validation repeats. Sample sizes (*n* biologically independent samples) for within-host validation datasets. Bovine intestinal: Diarrhea_1 (*n* = 19; 9 controls, 10 cases), Diarrhea_2 (*n* = 42; 14 controls, 28 cases), *Salmonella* infection (*n* = 48; 30 controls, 18 cases). Bovine non-intestinal: Mastitis (*n* = 30; 10 controls, 20 cases), Low body condition score (BCS) (*n* = 32; 16 controls, 16 cases), Hyperketonemia (*n* = 21; 11 controls, 10 cases). Canine intestinal: *Parvovirus* infection (*n* = 26; 14 controls, 12 cases), CE_1 (*n* = 27; 16 controls, 11 cases), CE_2 (*n* = 22; 17 controls, 5 cases). Canine non-intestinal: Epilepsy_1 (*n* = 22; 12 controls, 10 cases), Epilepsy_2 (*n* = 28; 14 controls, 14 cases), Leishmaniasis (*n* = 26; 14 controls, 12 cases), Hyperadrenocorticism (*n* = 24; 9 controls, 15 cases).

Given that health conditions often exhibit overlapping gut microbiome signatures^12,45^, we explored whether the diarrhea/IBD-associated signatures identified in our meta-analysis could be validated across multiple disease phenotypes within the same host. Signature-based models consistently outperformed on intestinal versus non-intestinal conditions in both hosts, with a larger gap in canine (random forest (RF): 92.0% vs. 60.4%; neural network (NN): 92.0% vs. 58.3%; mean AUC across validation datasets and repeats; Fig. 4a, b; sensitivity and specificity in Supplementary Fig. 8a) than bovine datasets (RF: 70.0% vs. 54.2%; NN: 67.0% vs. 54.2%), while achieving performance broadly comparable to full-genus models. In bovine datasets, signatures showed limited generalizability to *Salmonella* infection in adult cattle, possibly reflecting age-related microbiome differences between neonatal source cohorts and adult validation data^46^. Predictive performance was strongly correlated with the effect size of signature-associated microbiome dysbiosis between cases and controls (Spearman ρ = 0.90 for RF and 0.89 for NN in bovine, ρ = 0.71 and 0.84 in canine, all *p* < 0.05; Fig. 4c, d), suggesting that predictive performance is driven by the degree to which these signatures are dysregulated in the target condition.

To identify clues to model interpretability, we characterized signature associations across disease phenotypes using MaAsLin2. In bovine datasets (Fig. 4e), the two diarrhea cohorts showed consistent enrichment of *Olsenella* and depletion of *Ruminococcus*, *Bacteroides*, and *Oscillospira* (FDR-corrected *p* < 0.10; Fig. 4e), though coverage of signature genera was incomplete in both cohorts owing to metagenomic-to-16S taxonomic mismatches. The low body condition score (BCS) bovine cohort also showed depletion of *Bacteroides* and *Clostridia vadinBB60 group* (FDR-corrected *p* < 0.10). In canine datasets (Fig. 4f), the chronic enteropathy (CE) and *Parvovirus* infection cohorts showed the strongest signals (FDR-corrected *p* < 0.10 in most taxa; Fig. 4f), with enrichment of *Clostridium sensu stricto 1* and *Terrisporobacter* also observed in epilepsy, and depletion of *Allobaculum*, *Prevotella 9* and *Megamonas* in leishmaniasis, suggesting shared potential pathobionts across phenotypes that may partially account for the cross-disease generalizability of the signature models.

To further assess discriminative capacity, we trained a multi-disease classifier pooling all five canine validation studies (all adult dogs, avoiding the age confound observed in bovine; Supplementary Data 2), excluding the SMG-based CE study to ensure platform consistency, and retaining the larger of two epilepsy cohorts (Supplementary Data 2). Per-class AUC was still highest for intestinal conditions: CE achieved median AUCs of 91.3% (95% CI 85.3–92.1%) under RF and 84.1% (81.9–88.3%) under NN, and *parvovirus* infection 89.1% (80.9–92.0%) and 92.0% (86.3–92.4%), respectively. Non-intestinal conditions yielded lower but above-chance performance for hyperadrenocorticism (RF: 75.4% [64.2–78.1%]; NN: 68.1% [60.9–76.5%]) and leishmaniasis (RF: 73.6% [65.2–80.5%]; NN: 60.4% [47.9–68.1%]). The Normal class (control samples in each study) was also well-discriminated (RF: 84.0% [78.3–85.9%]; NN: 74.0% [69.9–76.3%]). The similar pattern in AUPR is present in Supplementary Fig. 8b. These results demonstrate that a reduced set of intestinal disease-associated taxa retains multi-class discriminative power, supporting potential utility in clinical screening for canine diseases, with analogous multi-disease prediction in bovine warranting future exploration as appropriate datasets become available.

### The identified microbes may help predict intestinal diseases for multiple mammalian hosts

**Fig. 5:**
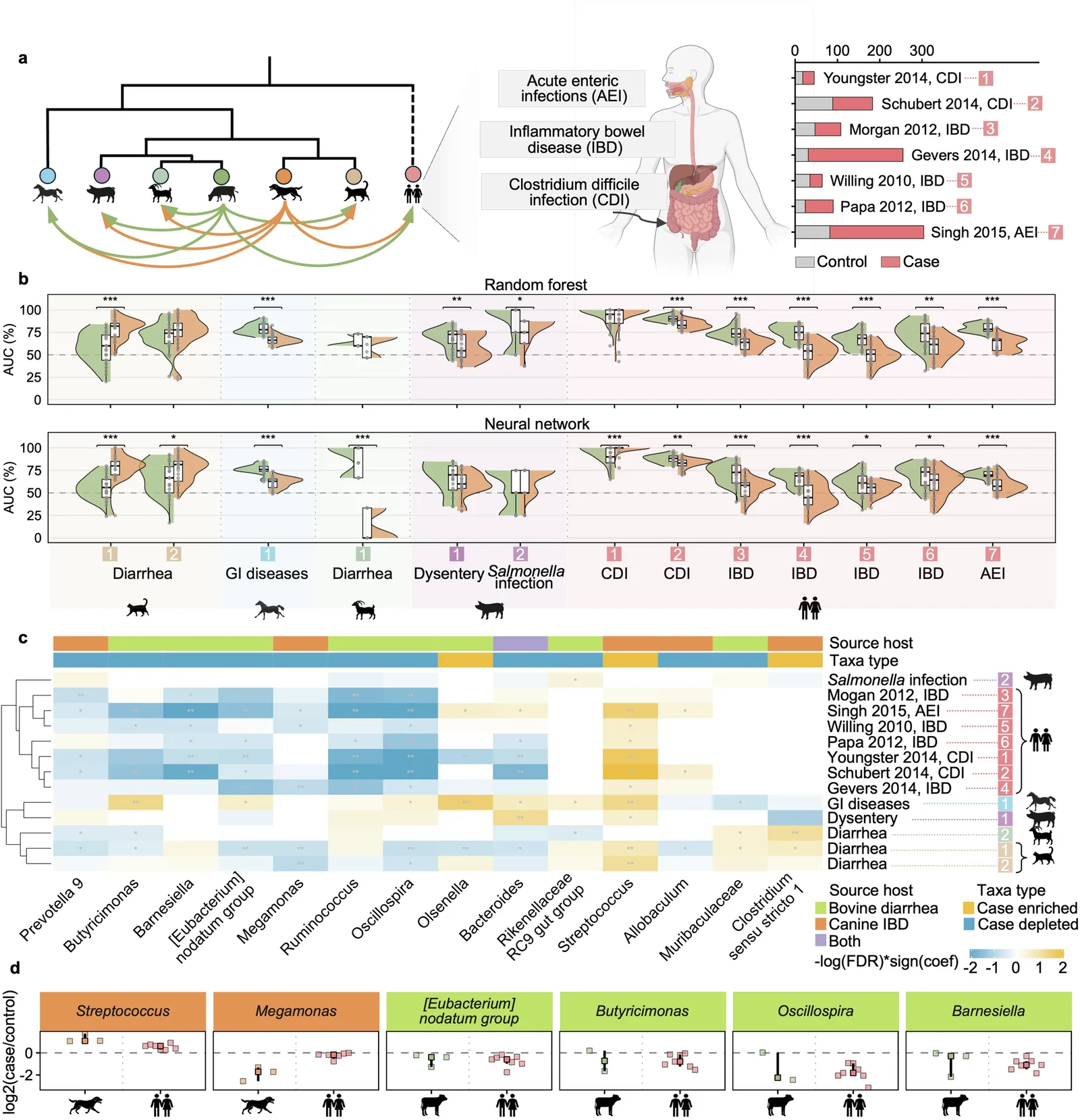
Cross-host intestinal validation of bovine diarrhea and canine IBD- associated signatures. **a** Schematic overview of the cross-host validation. Bovine diarrhea (green arrows) and canine IBD (orange arrows)-associated stable signatures were applied to fecal microbiome datasets from other domestic mammals (feline, equine, caprine, swine) and humans. Human intestinal disease datasets (Clostridium difficile infection [CDI], IBD, and acute enteric infections [AEI]) were retrieved from MicrobiomeHD; sample sizes are shown for each dataset. Illustrations of the human and gastrointestinal tract were created with BioRender (Lin, D. (2026) https://BioRender.com/kg8nx5q). **b** Cross-host classification performance (AUC) of Random Forest and Neural Network models, trained using bovine diarrhea-associated (green) or canine IBD-associated (orange) signature genera and evaluated on held-out datasets from each host species via 5 repeats × 5-fold cross-validation. AUC distributions are shown as violin plots with embedded boxplots, with individual repeat values overlaid as points. FDR-corrected pairwise comparisons between bovine and canine signatures: *p < 0.10, **p < 0.01, ***p < 0.001. **c** MaAsLin2 association heatmap of genera shared between bovine diarrhea and canine IBD-associated signatures across all cross-host validation datasets. Color reflects –log(FDR) × sign(coefficient), indicating direction and significance of association; asterisks denote FDR-corrected significance (*p < 0.10, **p < 0.01, ***p < 0.001). Annotation bars indicate the signature source host (green for bovine, orange for canine, purple for both) and taxa type (yellow for case-enriched, blue for case-depleted). **d** Log₂(case/control) abundance ratios of six genera shared between bovine diarrhea and canine IBD signatures across animal and human intestinal disease datasets. Each host species is identified by a silhouette icon. Individual data points (lighter shade) represent per-dataset log₂(case/control) ratios; darker points with black outlines indicate the median, and error bars represent 95% confidence intervals of the median. In all boxplots, the center line indicates the median, box limits represent the 25th and 75th percentiles, and whiskers extend to 1.5× the interquartile range. Sample sizes (*n* biologically independent samples) for animal cross-host validation datasets: feline diarrhea (*n* = 48; 24 controls, 24 cases and *n* = 58; 15 controls, 43 cases), swine *Salmonella* infection (*n* = 15; 7 controls, 8 cases), swine dysentery (*n* = 60; 53 controls, 7 cases), caprine diarrhea (*n* = 10; 5 controls, 5 cases), equine gastrointestinal (GI) disease (*n* = 342; 263 controls, 79 cases). Animal and human silhouettes were obtained from Freepik (https://www.freepik.com).

Finally, we examined the extent to which these intestinal disease-associated signatures generalize across other domestic mammals and humans (Fig. 5a). The canine IBD-associated signature generalized more effectively to feline diarrhea than the bovine diarrhea-associated signature, despite comprising fewer genera (8 vs. 11 and 8 vs. 10 genera retained, Supplementary Table 3; cohort 1, RF: 80% [51–96%] vs. 60% [23–81%], Wilcoxon signed-rank test, *p* < 0.001; NN: 78% [63–96%] vs. 56% [27–78%], *p* < 0.001; cohort 2, RF: 76% [56–96%] vs. 72% [49–93%]; NN: 79% [46–96%] vs. 66% [20–89%], *p* < 0.05; Fig. 5b), possibly reflecting their shared carnivorous dietary niche and associated gut microbiome composition^47^. In contrast, the bovine signature outperformed in other herbivorous hosts, including equine mixed-GI disease prediction (RF: 76% [68–88%] vs. 65% [57–75%]; NN: 74% [65–83%] vs. 62% [49–72%]; both *p* < 0.001) and caprine diarrhea prediction (NN: 96% vs. 5%, *p* < 0.001). The bovine signature also outperformed the canine signature across all human intestinal disease datasets, with average AUC ranging from 72–87% vs. 54–80% for *Clostridium difficile* infection (CDI) and IBD, and 76% vs. 65% for acute enteric infection (*p* < 0.05 for all datasets except Youngster 2014). The sensitivity and specificity are shown in Supplementary Fig. 9.

When we examined the relationship between these signatures and host intestinal disease phenotypes, we found that intestinal disease datasets from caprine, swine, equine and feline hosts clustered separately from most human datasets (Fig. 5c). Across hosts, the associated genera changed in the same direction as observed in the original bovine diarrhea and canine IBD datasets in most validation conditions. For example, in feline datasets, *Streptococcus* and *Clostridium sensu stricto 1* were enriched, while *Megamonas* and *Prevotella 9* were depleted (FDR-corrected *p* < 0.10), mirroring patterns observed in canine IBD. Notably, *Streptococcus* was also case-enriched across multiple intestinal disease datasets in multiple hosts (i.e., swine, equine and human), and *Megamonas* was consistently case-depleted across multiple human validation datasets (Fig. 5c, d). Several bovine diarrhea-associated signatures, including *[Eubacterium] nodatum group*, *Butyricimonas*, *Barnesiella*, and *Oscillospira*, were similarly case-depleted across multiple human intestinal disease datasets (Fig. 5c, d). These patterns suggest that intestinal diseases across domestic mammals share a conserved gut microbiome disturbance, characterized by the depletion of SCFA-producing and carbohydrate-fermenting commensals and the expansion of potential pathobionts, which may explain the cross-host generalizability of these signatures.

## Discussion

This study represents a comprehensive analysis of disease-associated fecal microbial patterns in domestic mammals. Building on the growing recognition of the gut microbiome’s role in human health status, we extended this paradigm to livestock and companion animals, whose microbiome–health relationships have been understudied despite their value as natural animal models for revealing conserved microbial shift patterns across mammalian hosts.

Meta-analysis of bovine diarrhea and canine IBD consistently revealed reduced fecal microbial diversity in disease^23,29,31,32^, aligned with prior observations in human gut disorders and individual animal cohorts^13^, suggesting that taxonomic diversity loss is a conserved signal of gut dysbiosis across mammals. Beyond diversity, both hosts exhibited a shared pattern of commensal depletion, comprising loss of polysaccharide-degrading *Bacteroidota* members and SCFA-producing *Firmicutes*, alongside host-specific pathobiont enrichment. These patterns were broadly consistent with individual cohort findings, notably the depletion of *Bacteroides*^29,31^, *Ruminococcus*^29,30^, *Oscillospira*^29^, and *Butyricimonas*^29^ in bovine diarrhea and the depletion of *Allobaculum*^23,32^, and enrichment of *Streptococcus*^8,23,32^ and *Clostridium sensu stricto 1*^8,32^ in canine IBD. Cross-species validation further extended this conserved pattern, with *Streptococcus* case-enriched across feline, swine, equine, and human intestinal disease datasets and several bovine diarrhea-associated SCFA-producing signatures consistently case-depleted across multiple human datasets. This cross-host consistency may reflect convergent evolution of gut dysbiosis under intestinal inflammation, whereby phylogenetically diverse hosts converge on similar microbial disruptions regardless of their distinct digestive physiologies. However, whether these shared taxonomic shifts translate to conserved functional and metabolic consequences, such as reduced SCFA production, impaired mucosal barrier integrity, and dysregulated immune homeostasis, and how these alterations interact with host genetics and physiology across species, remains to be established through integrative metatranscriptomic and metabolomic profiling across host species.

While host-specific enrichment patterns pointed to divergent pathobiological mechanisms, with canine IBD showing active pathobiont expansion suggestive of immune containment failure and bovine diarrhea enriched in mucosal barrier-eroding and lactate-producing taxa, these findings should be interpreted with caution, as the limited number of studies and samples, inter-study heterogeneity, and variation in disease severity and sequencing depth may mask genuine disease signals as false negatives or amplify study-specific effects as false positives. For instance, *Faecalibacterium* depletion, reported across the majority of constituent studies in both hosts^23,29,31,32^, and *Escherichia-Shigella* enrichment^8,10,23,29,32^, prominent in individual cohorts with acute pathogen-driven diarrhea, both fell short of our LODO meta-analysis pipeline. Furthermore, the retrospective case–control design of the constituent studies limits confounder removal. For example, IBD cases in two canine cohorts^8,32^ received hydrolyzed protein diets as part of the diagnostic workup while controls were fed standard commercial diets, introducing a dietary confound that may have driven the depletion of diet-sensitive taxa such as *Prevotella* independently of disease status. These limitations call for large-scale, standardized, well-annotated, prospectively collected datasets to disentangle disease-specific from confounding signals in animal microbiome meta-analysis.

Despite these limitations, the identified signatures demonstrated promising predictive potential across multiple clinical contexts. Within a host-disease setting, a small subset of stable genera performed comparably to full-feature models across multiple machine learning models, suggesting that the predictive signal is grounded in the underlying biology rather than being model-specific, and possibly supporting the feasibility of low-cost targeted detection approaches such as qPCR panels for intestinal disease screening in veterinary practice. At the cross-study level, transfer learning substantially improved generalization over direct model application, consistent with evidence from human cohorts that pretraining and fine-tuning can borrow disease-relevant microbial knowledge to aid diagnosis in data-limited settings^48^, a strategy particularly valuable in animal microbiome research where data scarcity remains a persistent constraint. The signatures also generalized more effectively to intestinal than non-intestinal conditions, suggesting convergence on common microbial disruptions across distinct gut diseases and supporting their potential utility as broad intestinal disease indicators, while limited transferability to non-intestinal phenotypes may reflect disease-type specificity. Finally, feline diarrhea was better predicted by canine IBD-associated signatures, and bovine diarrhea-associated signatures showed higher alignment with herbivorous hosts’ intestinal diseases, patterns that may reflect shared digestive ecology but warrant further investigation into their biological basis and potential utility under the One Health framework.

The findings of this study offer several concrete recommendations for future animal experimental designs. At the observational level, prospective multi-center case–control studies with standardized metadata collection, including age, breed, diet, disease severity, and sequencing platform, are essential to reduce confounding and enable robust cross-study comparison. Dietary composition should be matched between cases and controls at enrollment, and simultaneous collection of metatranscriptomic and metabolomic data alongside taxonomic profiling would enable functional validation of the taxonomic shifts identified here^49^. Increasing the number of independent studies per host-disease combination through coordinated multi-site data collection would also strengthen LODO-based stability assessment and cross-study generalization. At the experimental level, germ-free or gnotobiotic animal models selectively colonized with the depleted commensals or enriched pathobionts identified here could directly test their causal contributions to intestinal disease, distinguishing primary disease drivers from secondary responders^50^. Fecal microbiota transplantation from bovine diarrhea or canine IBD donors into germ-free recipients of other species could test whether the identified dysbiosis (e.g., convergent loss of SCFA-producing commensals and expansion of pathobionts) is sufficient to reproduce intestinal disease phenotypes across hosts, providing mechanistic insight into why these signatures generalize beyond their host of origin^51^. Such insight would advance our understanding of the shared pathobiological basis of intestinal diseases across domestic mammals, and could inform the development of broadly applicable diagnostic and therapeutic strategies.

In summary, although this study is constrained by limited animal fecal microbiome datasets, genus-level taxonomic resolution, and an observational design that precludes causal inference, the pipeline integrating leave-one-dataset-out meta-analysis with multi-layered cross-study, cross-disease, and cross-host validation yields robust intestinal disease-associated signatures in domestic mammals, though the generalizability of the pipeline, the practical utility of these signatures across clinical contexts, and the underlying biological mechanisms all remain subjects for future investigation.

## Methods

### Study collection

We first compiled a comprehensive list of domestic mammal disease-related studies on gut metagenome by searching in public databases including NCBI Sequence Read Archive (SRA) (https://www.ncbi.nlm.nih.gov/sra)^52^ and European Nucleotide Archive (ENA) (https://www.ebi.ac.uk/ena)^53^ based on exhaustive keywords such as “fecal microbiome”, “bovine OR cattle OR cow OR calf OR buffalo”, “canine OR dog”, “feline OR cat”, “swine OR pig OR sow”, “ovine OR caprine OR sheep OR goat”, and “equine OR horse” (as of February 2026). We also included one of our previous studies regarding bovine hyperketonemia^54^. The materials of each study were manually reviewed, and 35 studies were included under the following criteria: (1) an observational case–control study for a specific health event, with clearly defined group allocation criteria based on disease status or physiological condition (i.e., infected vs. uninfected), and (2) availability of 16S or SMG (shotgun metagenomics) microbiome fecal samples and corresponding metadata. Four of these studies were removed because they had unclear study design (i.e., lack of published paper which made it impossible to determine whether they followed a case–control design), included fewer than 10 total samples, or missing metadata (i.e., group assignment), even after contacting the authors.

Among the 31 retained studies, only bovine (calf) diarrhea had four independent 16S studies and canine IBD had three independent 16S studies, which enabled meta-analysis within these two host-disease models. Accordingly, we performed meta-analysis using these two disease-microbiome models, while the remaining studies were assigned to three validation scenarios based on host identity and disease type to assess the generalizability of identified microbial signatures (Fig. 2a). Diseases were broadly classified as intestinal or non-intestinal based on their primary site of pathology, consistent with the meta-analysis focus on intestinal diseases (diarrhea and IBD) involving direct fecal microbial disruption. This yielded the following categories: within-host intestinal (same host and disease type but cross-study), within-host non-intestinal (same host but cross-disease and cross-study), and cross-host intestinal (same disease type but cross-host). An additional five studies were excluded at this stage due to mixed host species or disease types that precluded consistent categorization. The original study list and the exclusion scheme are shown in Supplementary Data 1.

Within each of the remaining 26 studies, samples were excluded if they: (1) were undergoing treatment, post-treatment, or in the recovery phase; (2) involved antibiotic use; or (3) were not fecal samples. After this filtering, the initial 4,501 samples were reduced to 803 controls and 618 cases, including 125 controls and 185 cases from bovine (calf) diarrhea and 125 controls and 86 cases from canine IBD for meta-analysis. Specific information for each study, including sample sizes, diseases and conditions, sample exclusion criteria, and references, is shown in Supplementary Data 2.

### Profiling of 16S amplicon and shotgun metagenomic data

All 22 16S datasets were processed through our unified 16S processing pipeline. We downloaded raw sequencing data from their repositories before importing them into the Qiime2 (v 2024.10)^55^ microbiome bioinformatics platform using the q2-tools module. The primer sequences were first removed from each data set using the q2-cutadapt tool, and the open-source software package DADA2 (v 1.26)^56^ was used to de-noise the sequences into amplicon sequence variants (ASVs) using the q2-dada2 module with the trunc-len parameter of zero. Taxonomy was then assigned to ASVs based on SILVA (v 138) database^57^ with a Naïve Bayes classifier using the q2-feature-classifier module, where those classified as mitochondria or chloroplasts were removed. For downstream analyses, ASVs were collapsed to genus level using the q2-taxa modules. Finally, those taxa with prevalence <10% and relative abundance < 0.01% across all samples were removed to avoid noise caused by low-abundance taxa.

Raw metagenomic reads from four SMG datasets^58–61^ were quality filtered using fastp (v 0.23.1)^62^ based on a minimum Q Score of 20 and a minimum sequence length of 50 bp, and subsequently mapped to the reference genome of corresponding hosts (Bos taurus, GenBank accession number: GCA_002263795.4 and GCA_021347905.1; Canis lupus, GenBank accession number: GCA_011100685.1; Sus scrofa, GenBank accession number: GCA_000003025.6) using BWA-MEM with default parameters (v 0.7.17)^63^ to remove host reads. Remaining high-quality reads were used for genus-level metagenomic taxonomic profiling with MetaPhlAn4 (database: mpa_vJan25_CHOCOPhlAnSGB_202503; v 4.2.4)^64^ with default parameters. Given the methodological heterogeneity between 16S amplicon sequencing and shotgun metagenomic approaches, as well as the limited number of available SMG datasets (*n* = 4) relative to 16S datasets (*n* = 22), all following cross-study analysis (i.e., leave-one-dataset-out meta-analyses, leave-one-dataset-out machine learning and transfer learning, and multi-disease prediction) were performed exclusively on the 16S datasets, while the four SMG datasets were reserved for within-study validation analyses (i.e., cross-disease and cross-host validation).

### Meta-analysis for bovine diarrhea and canine IBD

As shown in Fig. 1, we performed a leave-one-dataset-out (LODO) random-effects meta-analysis separately for bovine (calf) diarrhea (four studies) and canine IBD (three studies) to identify microbial taxa consistently associated with disease across independent cohorts.

#### Compositional data transformation

For each study, zero counts in ASV table were replaced using the count zero multiplicative (CZM) method (R package zCompositions v 1.6.0^65^), with the imputed value set to 65% of the estimated upper threshold (frac = 0.65), followed by centered log-ratio (CLR) transformation.

#### Per-study effect size estimation

For each taxon in each study, a covariate-adjusted effect size was estimated by ordinary least squares regression of CLR-transformed values on disease status (case = 1, control = 0), with log-transformed sequencing depth and available demographic covariates (i. e., age, sex, body weight, breed group) included where applicable. The regression t-statistic for disease status was converted to a bias-corrected standardized mean difference (Hedges’ g^66^):

1 $$g=J\cdot t\sqrt{\frac{1}{{n}_{1}}+\frac{1}{{n}_{0}}},\,J=1-\frac{3}{4\left({n}_{1}+{n}_{0}-2\right)-1}$$

where ${{{{\rm{n}}}}}_{1}\,$ and ${{{{\rm{n}}}}}_{0}$ are case and control counts and ${{{\rm{J}}}}$ is the small-sample correction factor. The standard error was estimated as:

2 $${\mbox{SE}}=\sqrt{\frac{1}{{n}_{1}}+\frac{1}{{n}_{0}}+\frac{{g}^{2}}{2\left({n}_{1}+{n}_{0}\right)}}$$

#### Leave-one-dataset-out stability selection with random-effects meta-analysis

Per-study Hedges’ g estimates were combined into a pooled effect size (pooled Hedges’ g) using the DerSimonian–Laird random-effects model as implemented in R package metafor (v 4.8.0^67^). To identify taxa with consistent disease associations across independent cohorts, we applied a LODO framework: in each fold, one study was held out and the meta-analysis was performed on the remaining training studies (three bovine, two canine). Within each training fold, a taxon was retained as a candidate if the pooled Hedges’ g had FDR < 0.10, direction consistency exceeded 0.5, and it was present in ≥2 training studies. A taxon was considered stable if selected in ≥3 of 4 bovine folds (≥75%) or ≥2 of 3 canine folds (≥67%). The illustration of LODO meta-analysis is shown in Fig. 1.

#### Sensitivity analysis

To evaluate the robustness of the identified signatures to analytical parameter choices, we repeated the full LODO pipeline across 16 parameter combinations defined by four zero-replacement strategies (CZM with frac = 0.5, 0.65, or 0.8, and the geometric Bayesian multiplicative replacement method GBM (zCompositions v 1.6.0^65^)) and four FDR thresholds (0.05, 0.10, 0.15, and 0.20). For each combination, stable taxa were identified using the same LODO stability criteria to assess which of the selected signatures were consistently recovered.

### Machine learning models for validation

As shown in Fig. 1, we performed a three-step validation for generalizability of the identified bovine diarrhea/canine IBD-associated signatures. First, within-host cross-study generalizability was evaluated using LODO machine learning and transfer learning strategies. Second, within-host cross-disease generalizability was assessed by testing signature performance across intestinal and non-intestinal disease conditions. Third, cross-host generalizability was examined by applying the signatures to intestinal disease datasets from other domestic mammals and humans.

#### LODO machine learning for within-host cross-study validation

To evaluate the cross-dataset generalizability of the identified signatures, model evaluation also followed a LODO scheme, in which one study was held out as the test set while the remaining studies served as the training set, yielding four folds for bovine diarrhea and three folds for canine IBD. Five machine learning models were implemented using scikit-learn (v 1.6.1)^68^ and XGBoost (v 3.2.0)^69^ in Python 3.11: logistic regression with L1 regularization (LASSO), logistic regression with L2 regularization (Ridge), elastic net (SGDClassifier with combined L1/L2 penalty), random forest (RF), and gradient boosting (XGBoost). These models were selected for their established performance on high-dimensional microbiome data and interpretability^35^. To prevent data leakage, feature selection was fully nested within each LODO fold, where signatures were identified by re-running the covariate-adjusted DerSimonian–Laird random-effects meta-analysis exclusively on the training studies of that fold. This produced a per-fold signature feature set, which was compared against the full feature set (all taxa present in ≥3 of 4 bovine studies or ≥2 of 3 canine studies). Hyperparameters were tuned within each fold using inner three-fold stratified cross-validation (GridSearchCV, scoring = the area under the ROC curve (AUC)), searching over the following grids: RF (n_estimators ∈ {50, 100, 200, 500, 1000}, max_depth ∈ {None, 10, 20}, min_samples_leaf ∈ {1, 3, 5}); LASSO and Ridge (C ∈ {0.001, 0.01, 0.1, 1, 10, 100}); elastic net (α ∈ {0.0001, 0.001, 0.01, 0.1, 1}, l1_ratio ∈ {0.15, 0.5, 0.85}); XGBoost (n_estimators ∈ {50, 100, 200, 500, 1000}, max_depth ∈ {3, 6}, learning_rate ∈ {0.01, 0.1}). To address class imbalance, class weights were set to “balanced” for LASSO, Ridge, elastic net, and RF, whereas XGBoost used scale_pos_weight = n_negative/n_positive. Performance was assessed on each held-out study using the AUC, sensitivity, and specificity.

#### LODO transfer learning for within-host cross-study validation

To assess whether pre-training on source datasets and fine-tuning on the target dataset can further improve generalization to a held-out target study, we implemented a transfer learning framework (Fig. 1) using multilayer perceptron (MLP) classifiers (scikit-learn MLPClassifier^68^), applying the same per-fold signatures as the LODO pipeline. Neural networks were chosen as they support weight-based fine-tuning, enabling pre-trained models to adapt to a new dataset via continued gradient descent^70^. Three candidate architectures were evaluated via inner three-fold stratified cross-validation on the source training data: shallow (32 units), medium (64–32 units), and deep (64–32–16 units), all using ReLU activations, L2 regularization (α = 0.001), learning rate 0.001, and early stopping (patience = 20 iterations, validation fraction = 15%).

For each LODO fold, the target study was split into 75% and 25% subsets using stratified random sampling, repeated 20 times with different random seeds. All three strategies were evaluated on the identical 25% test set within each repeat. The LODO cross-validation strategy replicated the same LODO machine learning design as above but using the MLP classifier, training on the source studies (three for bovine diarrhea, two for canine IBD) and evaluating directly on the 25% test set to serve as a baseline for comparison with the transfer learning strategies. The LODO transfer strategy pre-trained the network on the source studies, then fine-tuned it on the 75% target training subset in two sequential phases: a linear probe phase with the encoder frozen (100 epochs, lr = 0.0001), followed by full-network fine-tuning with early stopping (up to 100 additional epochs, lr = 0.0001, patience = 10 on validation AUC), before evaluating on the held-out 25% test set. The LODO transfer with ComBat strategy using pyComBat package (v 0.20)^71^ followed the same procedure, but ComBat batch correction was only applied to the source training data prior to pre-training. AUC, sensitivity, and specificity were recorded on the 25% test set for each repeat, across both signature and full feature sets.

#### Within-host cross-disease validation

To evaluate whether the LODO-derived gut microbiome signatures generalize beyond the index disease, the identified stable signatures were applied as feature sets to independent validation cohorts encompassing a broader range of conditions within the same host: six bovine intestinal and non-intestinal studies (two independent diarrhea cohorts^58,59^, *Salmonella* infection^72^, mastitis^73^, low body condition score (BCS)^74^, and hyperketonemia^54^) and seven canine studies (*parvovirus* infection^75^, two independent chronic enteropathy (CE) cohorts^60,76^, two independent epilepsy cohorts^77,78^, leishmaniasis^79^, and hyperadrenocorticism^80^). For each validation dataset, two feature sets were evaluated: the LODO-stable signature genera and a full feature set. RF and MLP were selected for cross-disease evaluation as they represent complementary learning paradigms. Performance (AUC, sensitivity, and specificity) was assessed via five × five stratified repeated cross-validation with inner three-fold hyperparameter tuning using the same search spaces described above.

#### Within-host multi-disease prediction

To further assess whether the LODO-derived stable signatures can simultaneously distinguish multiple disease states, all 16S-based canine validation studies were pooled into a five-class classification framework (*parvovirus* infection, CE, epilepsy, leishmaniasis, hyperadrenocorticism, and a shared “Normal” class). As all included dogs were adults, this design avoided the age confound present in bovine studies. To counteract unequal study contributions across disease classes, study-aware sample weighting was applied to the RF and study-aware oversampling to the MLP. Model tuning and evaluation followed the same nested five × five stratified repeated cross-validation framework with inner three-fold hyperparameter tuning using the same search spaces. To mitigate batch effects, ComBat correction^71^ was applied to each training partition using study identity as the batch variable, with the resulting correction parameters then applied to the corresponding held-out test fold to prevent data leakage. Performance was reported as weighted one-vs-rest AUC and weighted Area Under the Precision-Recall Curve (AUPR) averaged across all repetitions.

#### Cross-host intestinal validation

To evaluate whether these fecal microbiome signatures associated with bovine and canine intestinal diseases generalize across mammalian species, each signature set was applied independently as a fixed feature set to validate cohorts spanning five additional hosts: equine (mixed gastrointestinal (GI) disease)^81^, feline (two diarrhea cohorts)^82,83^, caprine (diarrhea)^84^, swine (dysentery and *Salmonella* infection)^61,85^, and human (three *Clostridioides difficile* infection cohorts, enteric diarrheal disease, and three IBD cohorts, sourced from the MicrobiomeHD database^27^). The model configurations, and nested five × five stratified repeated cross-validation with inner three-fold hyperparameter tuning followed the same procedure as within-host cross-disease validation. Performance was reported as AUC, sensitivity, and specificity averaged across all repetitions.

### Statistics and reproducibility

#### Microbiome analyses

Alpha diversity metrics (Chao1 and Shannon index) were calculated by using the R package vegan (v 2.6.10)^86^ per group per study, while Faith’s phylogenetic diversity (Faith PD) was computed using the picante package (v 1.8.2)^87^. *P*-values for normal-disease comparisons were determined by the Wilcoxon rank-sum test. PERMANOVA was performed with vegan (v 2.6.10)^86^, for which the effect size (R^2^) of healthy statuses on microbiome variation was calculated using the adonis function, and the *p*-value was generated based on 999 permutations. Distance-based redundancy analysis (dbRDA) was performed on Bray–Curtis dissimilarities using the dbrda function in vegan (v 2.6.10)^86^, conditioning on study origin, sequencing platform, host breed, and geographic location. Genus-level associations with disease status were identified using MaAsLin2^88^, with healthy controls as the reference group. Available metadata including age, sex, sequencing read depth, and sequencing batch were included as covariates to control for potential confounders. Multiple testing was corrected using the Benjamini–Hochberg procedure, with a false discovery rate (FDR) threshold of 0.10.

#### Other analyses

All processed data were analyzed in the R 4.3.2 platform. Two-sided Wilcoxon rank-sum tests were used for all case–control comparisons, and *p*-value < 0.05 was considered statistically significant. For paired comparisons of model performance across feature sets or models, two-sided Wilcoxon signed-rank tests were used with *p*-value < 0.05 as the significance threshold.

### Ethics declarations

This study analyzed publicly available sequencing data from previously published studies; no new human or animal samples were collected. Accordingly, no Institutional Animal Care and Use Committee (IACUC) or Institutional Review Board (IRB) approval was required.

### Reporting summary

Further information on research design is available in the Nature Portfolio Reporting Summary linked to this article.

## Supplementary information

Transparent Peer Review file

Supplementary Information

Description of Additional Supplementary files

Supplementary Data 1

Supplementary Data 2

Reporting Summary

## Data Availability

All data used in the article are either publicly available or have been directly obtained from the authors of the original publications, as summarized in Supplementary Data 2: NCBI’s Sequence Read Archive (SRA) with the accession number PRJEB25741 for Whon et al. ^10^, PRJNA918869 for Li et al.^29^, PRJNA716761 for Chen et al.^30^, PRJNA1263132 for Zhang et al.^31^, PRJNA956982 for Shi et al.^58^, PRJNA1184454 for Li et al.^59^, PRJEB25138 for Muñoz-Vargas et al.^72^, PRJNA1095683 for Zhu et al.^73^, PRJNA1173238 for Saraphol et al.^74^, PRJNA905458 for Díaz-Regañón et al.^32^, PRJEB13362 for Vázquez-Baeza et al.^23^, PRJNA861113 for Doulidis et al.^8^, PRJNA474706 for Park et al.^75^, PRJNA1012802 for Kaga et al.^76^, PRJNA1167336 for Vecchiato et al.^60^, PRJNA746550 for García-Belenguer et al.^77^, PRJNA612483 for Muñana et al.^78^, PRJNA837097 for Meazzi et al.^79^, and PRJNA1150743 for Kang et al.^80^, PRJNA521510 for Argüello et al.^85^, PRJNA1218713 for Pérez et al.^61^, PRJNA436893 for Wang et al.^84^, PRJNA908214 for Bai et al.^82^, SRP047088 for Suchodolski et al.^83^, and PRJNA1032075 for McAdams et al^81^. The MicrobiomeHD dataset validated in this study is available on Zenodo: https://zenodo.org/records/840333^27,89^. Source data for the figures in this study are available at: https://doi.org/10.5281/zenodo.20693754^90^. Any remaining information can be obtained from the corresponding author upon reasonable request.

## Code availability

Code developed in this study is available at the GitHub repository: https://github.com/lindan1128/Mammal-FecalMicrobiome-Project and the Zenodo repository: https://doi.org/10.5281/zenodo.20693754^90^.
