# Supplementary material for: Toward disease-associated fecal microbiome signatures for domestic mammals
Source: Commun Biol. 2026 Jul 14;9:1474. doi: 10.1038/s42003-026-10647-5 (PMC13737101; doi:10.1038/s42003-026-10647-5)
Supplement: Supplementary file 1 [file 42003_2026_10647_MOESM1_ESM.pdf]

nature portfolio

Peer Review File

Toward diseases-associated fecal microbiome signatures for domestic mammals

Corresponding Author: Dr Dan Lin

This file contains all reviewer reports in order by version, followed by all author rebuttals in order by version.

Attachments originally included by the reviewers as part of their assessment can be found at the end of this file.

Version 0:

Reviewer comments:

Reviewer #1

(Remarks to the Author)
The study presented a unified pipeline aiming to identify a robust set of microbiome signatures for certain intestinal pathological conditions by conducting a metadata analysis of published reports. The authors did not generate any new experimental data. While the study is seemingly interesting, the general utility of the pipeline is not fully discussed and questionable, particularly in the era of rapidly evolving LLM/AI models.

1. L159, Define “marginal separation”. L180, replace the word MOST with a number or %.
2. L189, define “the key taxa”. What is the mean % of the key taxa retained after two rounds of corrections in all cases.
3. Repeated reference citations (e.g., L206 – L234) for individual studies are unnecessary.
4. The authors should also focus on the discrepancy in major findings between this meta-analysis and the original publications.
5. A paragraph In the Discussion session on recommendations for future animal experimental designs would be helpful, based on what is learned from the current study.

Reviewer #2

(Remarks to the Author)
Authors present a cross-study microbiome meta-analysis in animals to identify a reduced set of disease associated genera for calf diarrhea and canine IBD. At the core of the paper is a differential abundance + filtering pipeline used to define them. It is followed by machine learning to claim robustness and generalizability.

I have several major concerns related to the applied methodology and consecutively validity of the discovered signature and predictive performance.

In my opinion the evaluation design likely produces overly optimistic results and the meta-analysis is not conducted in a rigorous manner.

Point1. Feature selection OUTSIDE cross-validation = OVERLY OPTIMISTIC AUC

Authors define disease-associated genera via prevalence filtering, a hard log2FC threshold computed from mean relative abundances and Wilcoxon rank-sum + BH-FDR. Then the resulting reduced genus set is used for model training/evaluation. Afterwards authors conclude that >85% feature reduction maintains performance. This pipeline is problematic if the signature selection is done once using the full dataset and only then cross-validation is applied (which is how it is described). This creates information leakage = validation folds influence which genera are allowed into the model. Then AUC/accuracy is strongly inflated.

VERY IMPORTANT: redo ALL ML analyses with NESTED selection. Within each fold recompute the full signature selection only on the training split, then evaluate on held-out samples. Also report stability of selected genera across folds (otherwise

“signature” is not stable).

Point2. Meta-analysis is NOT a real meta-analysis

Authors call the work a meta-analysis but the main differential abundance step appears to be a pooled procedure (filters + Wilcoxon + BH correction) on merged data. At the same time authors themselves show that study origin - platform explain far more variance than disease status.
In such setting, pooled rank tests can identify study-driven taxa as “disease signatures” even if direction differs between studies. Therefore the claimed of robust disease-associated genera list is not convincingly supported.

One shall compute per study effect sizes for each genus and combine them formally (random-effects meta-analysis) or at minimum provide leave one study out analysis showing that the discovered signature is stable and not driven by one study.

Point3. Batch correction in transfer learning may leak target-study information

For multi-disease prediction authors state ComBat is applied within each CV iteration which is GOOD and correct. However in transfer learning sections it is stated that batch correction was performed “before model development” to assess transferability. This is not ok if ComBat parameters are estimated using any samples that later appear in the target test split. This is again leakage and can artificially increase cross-study transfer AUC.

Authors shall clarify and ensure that ComBat/MMUPHin fitting is always done on training data only and never using target-test samples.

Point4. Strange Random Forest setup = 20 trees to avoid overfitting is not justified

Authors use RF with 20 trees and justify it by avoiding overfitting. This is unusual. RF typically becomes more stable with more trees overfitting is not controlled by using an extremely small number of trees, but rather by depth and min-samples and proper validation. With 20 trees results can be unstable and sensitive to random seed.

Please tune RF hyperparameters properly (nested CV) and show sensitivity of results to number of trees. If the signature set changes then it is not robust.

Point5. Relative abundance + hard thresholds + pseudocount = biased and unstable signature genera

The signature discovery uses relative abundances, mean-based fold change with an arbitrary pseudocount + hard thresholds. This combination is known to be unstable in sparse compositional microbiome data and can produce spurious enrichments or miss moderate but consistent effects.
Authors mention alternative DA methods like MaasLin2, ALDEx2, etc but the headline signature still comes from the threshold-based pipeline.

One shall redo signature discovery using compositional aware log-ratio approaches (CLR with zero handling) and report sensitivity to pseudocount and threshold choices.

Reviewer #3

(Remarks to the Author)
Comments for the Authors
The authors present a well-structured and informative study that systematically investigates fecal microbiome signatures associated with intestinal diseases in domestic mammals, using cross-study integration alongside multi-disease and cross-host validation to assess the robustness and transferability of these signals. The abstract effectively communicates the overall motivation within a One Health framework and outlines a coherent analytical strategy to address study heterogeneity and batch effects. Overall, the work appears carefully executed, and the study design is logically aligned with the stated objectives. While the main message is clear, several points could be refined to improve readability and to better delineate the scope and interpretation of the conclusions.

Major Comments
1. Could the authors clarify the rationale for focusing the meta-analysis on canine and bovine cohorts? For example, it would be helpful to understand whether this choice was driven primarily by data availability, study comparability, clinical relevance, geographic representation, or an a priori hypothesis about cross-host transferability.
2. The manuscript appears to apply a neural network approach (like a deep neural network; DNN) alongside other classifiers. Could the authors briefly explain why this model family was selected for the present task and what advantages were expected in this setting? Making this motivation more explicit in the Methods and/or Introduction may help readers interpret the modeling choices.
Minor Comments
1. Throughout the manuscript, there appear to be several minor issues such as omitted abbreviations, typographical errors, and formatting inconsistencies. A careful proofreading pass to correct these points would improve overall clarity and readability. (e.g. Page 4, Line 113 & 115: 3 → three; Page 6, Line 205: AUC; Page 8, Line 258: AUPR; etc.)
2. Figure 1: It may improve clarity if the authors more explicitly indicate how datasets are partitioned (e.g., within-study vs

cross-study splits; train/validation/test) and where batch correction is applied relative to data splitting and model training. A small annotation or step numbering could help readers follow the workflow.
3. Figure 2: The overall readability could be improved. In particular, creating a clearer visual separation between panels D and E could help. For panels F–I, placing index labels in a consistent position (e.g., aligned on the left) could improve uniformity and make the figure easier to scan.
4. Page 5, Lines 169–177 & Figure 3: It may be helpful to align the order of panel descriptions with the panel order (e.g., by swapping the order of 3B and 3C, or adjusting the narrative accordingly) to improve coherence. In addition, several text elements appear to overlap within the figure; reducing overlap and improving spacing would enhance legibility.
5. Page 6, Lines 205–207: Referring to “studies 1, 2, 3” may be slightly difficult to parse. Using a more explicit and consistent convention (e.g., “Study (1)”, “Study (2)”, “Study (3)” or “Study_1/ Study_2/ Study_3”) may improve readability, especially if it matches the figure labels.
6. Figure 4A: The overall pipeline may be difficult to follow immediately. Could the authors consider making the directionality and sequence of steps more explicit (e.g., clearer arrows) to help readers understand the processing flow?

Reviewer #4

(Remarks to the Author)
Lin et al. tackle a highly significant problem in this study, and their methodological approach is sound. My primary concern lies with the limited sample size, which is a critical consideration for a meta-analysis and makes it challenging to fully assess the robustness of the findings. The study's impact would be greatly enhanced by incorporating a larger and more diverse set of datasets to validate the proposed disease-associated signatures.

Version 1:

Reviewer comments:

Reviewer #2

(Remarks to the Author)
The authors have redesigned the analytical pipeline in line with my critiques. The pooled Wilcoxon approach is replaced with a formal random-effects meta-analysis embedded in a leave-one-dataset-out framework. CLR-based compositional handling with a 16-combination sensitivity analysis dis OK. Hyperparameter tuning is now proper and ComBat application to training data only is now explicitly stated. I recommend acceptance pending one minor correction: supplementary figure references on lines 387–388 are off-by-one (should be Figure S2 and Figure S3 respectively).

Reviewer #3

(Remarks to the Author)
The authors have provided a meticulous response to the reviewers' comments. The revised manuscript is well-structured and significantly improved in terms of clarity and flow. I believe the paper is now well-suited for publication in its current form.

Reviewer #4

(Remarks to the Author)
Thank you to the author for the revisions. After carefully reviewing the updated submission, I confirm that all my previous comments have been satisfactorily addressed. I have no further corrections or objections. Therefore, I recommend acceptance of the paper as is.

Open Access This Peer Review File is licensed under a Creative Commons Attribution 4.0 International License, which permits use, sharing, adaptation, distribution and reproduction in any medium or format, as long as you give appropriate credit to the original author(s) and the source, provide a link to the Creative Commons license, and indicate if changes were made.
In cases where reviewers are anonymous, credit should be given to 'Anonymous Referee' and the source.
The images or other third party material in this Peer Review File are included in the article's Creative Commons license, unless indicated otherwise in a credit line to the material. If material is not included in the article's Creative Commons license and your intended use is not permitted by statutory regulation or exceeds the permitted use, you will need to obtain permission directly from the copyright holder.
To view a copy of this license, visit https://creativecommons.org/licenses/by/4.0/

Point-by-Point Response to Reviewers

Manuscript: COMMSBIO-25-12342

"Toward diseases-associated fecal microbiome signatures for domestic mammals"

We thank the editor and reviewers for the constructive feedback. In response to Reviewer #1, we clarified the utility of the pipeline and added Discussion paragraphs on discrepancies with original publications and future experimental design recommendations. In response to Reviewer #2, we redesigned the entire analytical pipeline, replacing the pooled analysis with a compositionally aware LODO random-effects meta-analysis, implementing fully nested feature selection and hyperparameter tuning in ML, and ensuring no data leakage in batch correction. In response to Reviewer #3, we clarified the rationale for host and model selection, improved figure readability, and performed thorough proofreading. In response to Reviewer #4, we expanded the datasets and acknowledged sample size limitations throughout the manuscript.

Below, we provide a detailed point-by-point response to each comment. Reviewer comments are shown in black, our responses are shown in blue, and revised text from the manuscript is highlighted in red. Revised main figures are included at the end of this letter. All reference numbers cited in this letter correspond to those in the Manuscript.pdf.

Reviewer #1

The study presented a unified pipeline aiming to identify a robust set of microbiome signatures for certain intestinal pathological conditions by conducting a metadata analysis of published reports. The authors did not generate any new experimental data. While the study is seemingly interesting, the general utility of the pipeline is not fully discussed and questionable, particularly in the era of rapidly evolving LLM/AI models.

A: We thank the reviewer for raising this point. The leave-one-dataset-out (LODO) meta-analysis framework with multi-layered cross-study, cross-disease, and cross-host validation developed here is a reusable methodological contribution for the animal microbiome field, where data scarcity remains a persistent constraint, and can be readily applied to other host-disease combinations as more datasets become available. The curated, biologically validated signatures identified through this pipeline could also serve as high-quality inputs for future AI/LLM-based clinical decision support systems in veterinary medicine.

We have discussed the potential practical utility of this pipeline in the fourth paragraph of the Discussion (Lines 341–357):

"Despite these, the identified signatures demonstrated promising predictive potential across multiple clinical contexts. Within a host-disease setting, a small subset of stable genera performed

comparably to full-feature models across multiple machine learning models, suggesting that the predictive signal is grounded in the underlying biology rather than model-specific, and possibly supporting the feasibility of low-cost targeted detection approaches such as qPCR panels for intestinal disease screening in veterinary practice. At the cross-study level, transfer learning substantially improved generalization over direct model application, consistent with evidence from human cohorts that pretraining and fine-tuning can borrow disease-relevant microbial knowledge to aid diagnosis in data-limited settings [48], a strategy particularly valuable in animal microbiome research where data scarcity remains a persistent constraint. The signatures also generalized more effectively to intestinal than non-intestinal conditions, suggesting convergence on common microbial disruptions across distinct gut diseases and supporting their potential utility as broad intestinal disease indicators, while limited transferability to non-intestinal phenotypes may reflects disease-type specificity."

We have also acknowledged in the last paragraph of the Discussion (Lines 377–383) that while the pipeline yields robust signatures, its generalizability and practical utility across clinical contexts remain subjects for future investigation:

"In summary, although this study is constrained by limited animal fecal microbiome datasets, genus-level taxonomic resolution, and an observational design that precludes causal inference, the pipeline integrating leave-one-dataset-out meta-analysis with multi-layered cross-study, cross-disease, and cross-host validation yields robust intestinal disease-associated signatures in domestic mammals, though the generalizability of the pipeline, the practical utility of these signatures across clinical contexts, and the underlying biological mechanisms all remain subjects for future investigation."

Comment 1.1: L159, Define "marginal separation". L180, replace the word MOST with a number or %.

A: Thanks. We replaced "marginal separation" with quantitative dbRDA results in Results (Lines 159–162):

"Even after adjusting for study origin and sequencing platform effects using Distance-based redundancy analysis (dbRDA, on Bray–Curtis distance), disease status explained only 4% and 3% of the residual variance in fecal microbial composition (bovine: constrained R^2 = 4%, F = 15.91, $p < 0.001$; canine: constrained R^2 = 3%, F = 3.46, $p = 0.004$; Figures 2h and 2i)."

The constrained R^2 values and corresponding p-values are also now annotated directly on Figures 2h and 2i.

Regarding "MOST," the original section heading (L180) was removed as part of the revised meta-analysis framework. The new section heading in Results reads "Gut dysbiosis featured a shared depletion of beneficial taxa alongside host-specific enrichment patterns" (Lines 165–166). Exact retention rates are now reported: "mean taxa retention across folds: 83.3% bovine, 91.7% canine" (Results, Lines 168–169).

Comment 1.2: L189, define "the key taxa". What is the mean % of the key taxa retained after two rounds of corrections in all cases.

A: The original two-round correction approach has been replaced with a leave-one-dataset-out (LODO) random-effects meta-analysis framework (Figure 1). A taxon is now defined as a stable signature if selected in ≥3 of 4 bovine folds (≥75%) or ≥2 of 3 canine folds (≥67%). This is described in Methods, "Meta-analysis for bovine diarrhea and canine IBD" (Lines 466–471):

"A taxa was considered stable if selected in ≥3 of 4 bovine folds (≥75%) or ≥2 of 3 canine folds (≥67%)."

Regarding the mean % of taxa retained, as described in Results (Lines 167–171):

"The Leave-One-Dataset-Out (LODO)-nested meta-analysis identified 12 bovine and 8 canine stable taxa consistently altered across studies (Figure 3a; mean taxa retention across folds: 83.3% bovine, 91.7% canine; each fold's results in Table S3 and S4), corresponding to all taxa significant in the full pooled meta-analysis for bovine and eight of nine for canine (Figure S3), and robust across CLR-based zero-handling parameterizations and FDR thresholds of 0.05–0.20 (Figure S4)."

Comment 1.3: Repeated reference citations (e.g., L206–L234) for individual studies are unnecessary.

A: We streamlined citations throughout the Results section so that individual study references are cited once at their point of introduction rather than repeated in subsequent sentences.

Comment 1.4: The authors should also focus on the discrepancy in major findings between this meta-analysis and the original publications.

A: We addressed this at multiple points in the revised manuscript.

In Results, we explicitly compared our findings with the original publications. Lines 140–142 note that genus-level profiles "were consistent with original study findings, and indicated the distinct genus-level gut microbiota profiles between diarrhea/IBD cases and healthy controls within each host, though inter-study variability was observed." Lines 143–152 further confirm that "all studies reproduced their original findings, with most datasets (2/4 in calf diarrhea and 2/3 in canine IBD) showing that community composition was significantly associated with disease status (PERMANOVA test on Bray–Curtis distance, $p < 0.05$, Figure 2d), further supported by weighted UniFrac and Jaccard metrics (Figure S1b)."

In Discussion, we discuss both consistencies and discrepancies in detail:

The second paragraph (Discussion, Lines 306–324) discusses **consistencies** between our meta-analysis and individual cohort findings, including conserved diversity loss, shared commensal de-

pletion patterns, and cross-species validation of these patterns:

"Meta-analysis of bovine diarrhea and canine IBD consistently revealed reduced fecal microbial diversity in disease [29,31,32,23], aligned with prior observations in human gut disorders and individual animal cohorts [13], suggesting that taxonomic diversity loss is a conserved signal of gut dysbiosis across mammals. Beyond diversity, both hosts exhibited a shared pattern of commensal depletion, comprising loss of polysaccharide-degrading *Bacteroidota* members and SCFA-producing *Firmicutes*, alongside host-specific pathobiont enrichment. These patterns were broadly consistent with individual cohort findings, notably the depletion of *Bacteroides* [29,31], *Ruminococcus* [30,29], *Oscillospira* [29], and *Butyricimonas* [29] in bovine diarrhea and the depletion of *Allobaculum* [32,23], and enrichment of *Streptococcus* [32,23,8] and *Clostridium sensu stricto 1* [32,8] in canine IBD. Cross-species validation further extended this conserved pattern, with *Streptococcus* case-enriched across feline, swine, equine, and human intestinal disease datasets and several bovine diarrhea-associated SCFA-producing signatures consistently case-depleted across multiple human datasets. This cross-host consistency may reflect convergent evolution of gut dysbiosis under intestinal inflammation, whereby phylogenetically diverse hosts converge on similar microbial disruptions regardless of their distinct digestive physiologies. However, whether these shared taxonomic shifts translate to conserved functional and metabolic consequences, such as reduced SCFA production, impaired mucosal barrier integrity, and dysregulated immune homeostasis, and how these alterations interact with host genetics and physiology across species, remains to be established through integrative metatranscriptomic and metabolomic profiling across host species."

The third paragraph (Discussion, Lines 325–340) discusses **discrepancies**, including taxa that were consistently reported in individual studies but did not pass our LODO pipeline (e.g., *Faecalibacterium*, *Escherichia-Shigella*), and potential confounding factors such as dietary differences:

"While host-specific enrichment patterns pointed to divergent pathobiological mechanisms, with canine IBD showing active pathobiont expansion suggestive of immune containment failure and bovine diarrhea enriched in mucosal barrier-eroding and lactate-producing taxa, these findings should be interpreted with caution, as the limited number of studies and samples, inter-study heterogeneity, and variation in disease severity and sequencing depth may mask genuine disease signals as false negatives or amplify study-specific effects as false positives. For instance, *Faecalibacterium* depletion, reported across the majority of constituent studies in both hosts [29,31,32,23], and *Escherichia-Shigella* enrichment [10,29,32,23,8], prominent in individual cohorts with acute pathogen-driven diarrhea, both fell short of our LODO meta-analysis pipeline. Furthermore, the retrospective case-control design of the constituent studies limits confounder removal. For example, IBD cases in two canine cohorts [32,8] received hydrolyzed protein diets as part of the diagnostic workup while controls were fed standard commercial diets, introducing a dietary confound that may have driven the depletion of diet-sensitive taxa such as *Prevotella* independently of disease status. These limitations call for large-scale, standardized, well-annotated, prospectively collected datasets to disentangle disease-specific from confounding signals in animal microbiome meta-analysis."

Comment 1.5: A paragraph in the Discussion section on recommendations for future animal

experimental designs would be helpful, based on what is learned from the current study.

A: We added a dedicated paragraph in Discussion (Lines 358–376) providing recommendations from two angles, the observational level (study design, metadata standardization, multi-omics) and the experimental level (gnotobiotic models, fecal microbiota transplantation):

"The findings of this study offer several concrete recommendations for future animal experimental designs. At the observational level, prospective multi-center case-control studies with standardized metadata collection, including age, breed, diet, disease severity, and sequencing platform, are essential to reduce confounding and enable robust cross-study comparison. Dietary composition should be matched between cases and controls at enrollment, and simultaneous collection of metatranscriptomic and metabolomic data alongside taxonomic profiling would enable functional validation of the taxonomic shifts identified here [49]. Increasing the number of independent studies per host-disease combination through coordinated multi-site data collection would also strengthen LODO-based stability assessment and cross-study generalization. At the experimental level, germ-free or gnotobiotic animal models selectively colonized with the depleted commensals or enriched pathobionts identified here could directly test their causal contributions to intestinal disease, distinguishing primary disease drivers from secondary responders [50]. Fecal microbiota transplantation from bovine diarrhea or canine IBD donors into germ-free recipients of other species could test whether the identified dysbiosis (e.g., convergent loss of SCFA-producing commensals and expansion of pathobionts) are sufficient to reproduce intestinal disease phenotypes across hosts, providing mechanistic insight into why these signatures generalize beyond their host of origin [51]. Such insight would advance our understanding of the shared pathobiological basis of intestinal diseases across domestic mammals, and could inform the development of broadly applicable diagnostic and therapeutic strategies."

Reviewer #2

Authors present a cross-study microbiome meta-analysis in animals to identify a reduced set of disease associated genera for calf diarrhea and canine IBD. At the core of the paper is a differential abundance + filtering pipeline used to define them. It is followed by machine learning to claim robustness and generalizability. I have several major concerns related to the applied methodology and consecutively validity of the discovered signature and predictive performance. In my opinion the evaluation design likely produces overly optimistic results and the meta-analysis is not conducted in a rigorous manner.

A: We thank the reviewer for these detailed and constructive critiques. We agree with the concerns raised and have redesigned the entire analytical pipeline accordingly. Below, we address each point in the order that follows the revised pipeline: (1) compositional data transformation (Point 2.5), (2) LODO random-effects meta-analysis for signature selection (Point 2.2), (3) nested feature selection within machine learning (ML) validation (Point 2.1), (4) ML hyperparameter tuning (Point 2.4),

and (5) batch correction in transfer learning (Point 2.3). The complete revised pipeline is illustrated in Figure 1 of the revised manuscript.

Point 2.5: Relative abundance + hard thresholds + pseudocount = biased and unstable signature genera.

The signature discovery uses relative abundances, mean-based fold change with an arbitrary pseudocount + hard thresholds. This combination is known to be unstable in sparse compositional microbiome data and can produce spurious enrichments or miss moderate but consistent effects. Authors mention alternative DA methods like MaAsLin2, ALDEx2, etc. but the headline signature still comes from the threshold-based pipeline.

One shall redo signature discovery using compositional aware log-ratio approaches (CLR with zero handling) and report sensitivity to pseudocount and threshold choices.

A: The threshold-based pipeline has been entirely replaced with a compositionally aware approach. Zero counts are now handled using the count zero multiplicative (CZM) method (zCompositions v 1.6.0, frac = 0.65), followed by centered log-ratio (CLR) transformation (Methods, "Meta-analysis for bovine diarrhea and canine IBD," Lines 450–453):

"Compositional data transformation. For each study, zero counts in ASV table were replaced using the count zero multiplicative (CZM) method (R package zCompositions v 1.6.0 [65]), with the imputed value set to 65% of the estimated upper threshold (frac = 0.65), followed by centered log-ratio (CLR) transformation."

To assess sensitivity to analytical parameter choices, we performed a systematic sensitivity analysis as described in the same section (Lines 472–477):

"Sensitivity analysis. To evaluate the robustness of the identified signatures to analytical parameter choices, we repeated the full LODO pipeline across 16 parameter combinations defined by four zero-replacement strategies (CZM with frac = 0.5, 0.65, or 0.8, and the geometric Bayesian multiplicative replacement method GBM (zCompositions v 1.6.0 [65])) and four FDR thresholds (0.05, 0.10, 0.15, and 0.20). For each combination, stable taxa were identified using the same LODO stability criteria to assess which of the selected signatures were consistently recovered."

As reported in Results (Lines 169–171), the identified signatures were "robust across CLR-based zero-handling parameterizations and FDR thresholds of 0.05–0.20 (Figure S4)."

Point 2.2: Meta-analysis is NOT a real meta-analysis.

Authors call the work a meta-analysis but the main differential abundance step appears to be a pooled procedure (filters + Wilcoxon + BH correction) on merged data. At the same time authors themselves show that study origin – platform explain far more variance than disease status. In such setting, pooled rank tests can identify study-driven taxa as "disease signatures" even if direction differs between studies. Therefore the claimed of robust disease-associated genera list is not

convincingly supported.

One shall compute per study effect sizes for each genus and combine them formally (random-effects meta-analysis) or at minimum provide leave one study out analysis showing that the discovered signature is stable and not driven by one study.

A: We replaced the pooled Wilcoxon-based pipeline with a formal random-effects meta-analysis, as described in Methods, "Meta-analysis for bovine diarrhea and canine IBD" (Lines 446–471). The revised pipeline consists of four steps, of which Step 1 (Compositional data transformation) and Step 4 (Sensitivity analysis) have been described in Point 2.5 above. The remaining two steps are:

"Per-study effect size estimation. For each taxon in each study, a covariate-adjusted effect size was estimated by ordinary least squares regression of CLR-transformed values on disease status (case = 1, control = 0), with log-transformed sequencing depth and available demographic covariates (i.e., age, sex, body weight, breed group) included where applicable. The regression t-statistic for disease status was converted to a bias-corrected standardized mean difference (Hedges' g [66])." (Lines 454–458)

"Leave-one-dataset-out stability selection with random-effects meta-analysis. Per-study Hedges' g estimates were combined into a pooled effect size (pooled Hedges' g) using the DerSimonian–Laird random-effects model as implemented in R package metafor (v 4.8.0 [67]). To identify taxa with consistent disease associations across independent cohorts, we applied a LODO framework: in each fold, one study was held out and the meta-analysis was performed on the remaining training studies (three bovine, two canine). Within each training fold, a taxa was retained as a candidate if the pooled Hedges' g had FDR < 0.10, direction consistency exceeded 0.5, and it was present in ≥ 2 training studies. A taxa was considered stable if selected in ≥ 3 of 4 bovine folds ($\geq 75\%$) or ≥ 2 of 3 canine folds ($\geq 67\%$). The illustration of LODO meta-analysis is shown in Fig 1." (Lines 463–471)

The LODO meta-analysis illustration is shown in Figure 1 of the revised manuscript. As reported in Results (Lines 167–171):

"The Leave-One-Dataset-Out (LODO)-nested meta-analysis identified 12 bovine and 8 canine stable taxa consistently altered across studies (Figure 3a; mean taxa retention across folds: 83.3% bovine, 91.7% canine; each fold's results in Table S3 and S4), corresponding to all taxa significant in the full pooled meta-analysis for bovine and eight of nine for canine (Figure S3), and robust across CLR-based zero-handling parameterizations and FDR thresholds of 0.05–0.20 (Figure S4)."

Hedges' g values with 95% CIs for each signature taxon are detailed in Results (Lines 172–193):

"In both hosts, the common depletion of *Bacteroidota* commensals, notably *Bacteroides* (bovine: median Hedges' g across folds = −0.35 [95% CI −0.44 to −0.26]; canine: g = −0.27 [−0.39 to −0.15]), along with *Rikenellaceae RC9 gut group* (g = −1.17 [−1.65 to −0.69]), *Barnesiella* (g = −0.50 [−0.65 to −0.34]) and *Muribaculaceae* (family-level) (g = −0.81 [−1.09 to −0.53]) in bovine, and *Prevotella 9* (g = −0.61 [−0.85 to −0.38]) and *Megamonas* (g = −0.74 [−1.08

to −0.40]) in canine, points to a compromised capacity for complex polysaccharide degradation [36,37]. This was accompanied by a broad loss of short-chain fatty acid (SCFA)-producing taxa in both hosts, particularly *Oscillospira* [38] (g = −0.82 [−1.07 to −0.57]) and *Ruminococcus* [38] (g = −0.22 [−0.30 to −0.15]), as well as *Butyricimonas* [39] (g = −0.45 [−0.63 to −0.27]) in bovine, and *Allobaculum* [40] (g = −0.52 [−0.67 to −0.36]) and *Peptoclostridium* [40] (g = −0.61 [−0.95 to −0.27]) in canine, reflecting a shared collapse of beneficial microbial communities."

"Distinct enrichment patterns characterized the gut microbial dysbiosis in each host-specific intestinal disease. Canine IBD was commonly marked by the proliferation of potential pathobionts including *Streptococcus* [8,41] (g = +0.68 [+0.65 to +0.72]), *Clostridium sensu stricto 1* [8] (g = +0.74 [+0.49 to +1.00]), and *Terrisporobacter* [42] (g = +0.51 [+0.39 to +0.63]), indicating a shift from passive commensal loss toward active pathobiont expansion, suggestive of immune containment failure. In contrast, bovine diarrhea was enriched in *[Ruminococcus] torques group* [43] (g = +0.27 [+0.17 to +0.36]) and *Olsenella* [44] (g = +0.33 [+0.22 to +0.44]), suggesting concurrent mucosal barrier erosion and lactate-driven suppression of commensal recovery. These findings reveal a conserved pattern of commensal depletion across both hosts, overlaid with host-specific compositional shifts pointing to divergent pathobiological mechanisms, though broader validation remains warranted."

Point 2.1: Feature selection OUTSIDE cross-validation = OVERLY OPTIMISTIC AUC.

Authors define disease-associated genera via prevalence filtering, a hard log2FC threshold computed from mean relative abundances and Wilcoxon rank-sum + BH-FDR. Then the resulting reduced genus set is used for model training/evaluation. Afterwards authors conclude that >85% feature reduction maintains performance. This pipeline is problematic if the signature selection is done once using the full dataset and only then cross-validation is applied (which is how it is described). This creates information leakage = validation folds influence which genera are allowed into the model. Then AUC/accuracy is strongly inflated.

VERY IMPORTANT: redo ALL ML analyses with NESTED selection. Within each fold recompute the full signature selection only on the training split, then evaluate on held-out samples. Also report stability of selected genera across folds (otherwise "signature" is not stable).

A: With signatures derived from the LODO meta-analysis (Point 2.2), we implemented a LODO machine learning framework to further validate them: in each LODO fold, the per-fold signatures selected by the meta-analysis on training studies are used as features for ML models, which are then evaluated on the held-out study. This evaluates whether these signatures retain predictive power on independent studies and whether the reduced signature set matches the discriminative capacity of the full genus set. Signature identification is fully nested within each fold to prevent data leakage. The complete procedure is described in Methods, "LODO machine learning for within-host cross-study validation" (Lines 485–506):

"LODO machine learning for within-host cross-study validation. To evaluate the cross-dataset generalizability of the identified signatures, model evaluation also followed a LODO scheme, in which

one study was held out as the test set while the remaining studies served as the training set, yielding four folds for bovine diarrhea and three folds for canine IBD. Five machine learning models were implemented using scikit-learn (v 1.6.1) [68] and XGBoost (v 3.2.0) [69] in Python 3.11: logistic regression with L1 regularization (LASSO), logistic regression with L2 regularization (Ridge), elastic net (SGDClassifier with combined L1/L2 penalty), random forest (RF), and gradient boosting (XGBoost). These models were selected for their established performance on high-dimensional microbiome data and interpretability [35]. To prevent data leakage, feature selection was fully nested within each LODO fold, where signatures were identified by re-running the covariate-adjusted DerSimonian–Laird random-effects meta-analysis exclusively on the training studies of that fold. This produced a per-fold signature feature set, which was compared against the full feature set (all taxa present in $\geq$3 of 4 bovine studies or $\geq$2 of 3 canine studies). Hyperparameters were tuned within each fold using inner three-fold stratified cross-validation (GridSearchCV, scoring = the area under the ROC curve (AUC)), searching over the following grids: RF (n_estimators $\in$ {50, 100, 200, 500, 1000}, max_depth $\in$ {None, 10, 20}, min_samples_leaf $\in$ {1, 3, 5}); LASSO and Ridge (C $\in$ {0.001, 0.01, 0.1, 1, 10, 100}); elastic net ($\alpha \in$ {0.0001, 0.001, 0.01, 0.1, 1}, l1_ratio $\in$ {0.15, 0.5, 0.85}); XGBoost (n_estimators $\in$ {50, 100, 200, 500, 1000}, max_depth $\in$ {3, 6}, learning_rate $\in$ {0.01, 0.1}). To address class imbalance, class weights were set to 'balanced' for LASSO, Ridge, elastic net, and RF, whereas XGBoost used scale_pos_weight = n_negative / n_positive. Performance was assessed on each held-out study using the AUC, sensitivity, and specificity."

Regarding signature stability across LODO meta-analysis folds: comparing per-fold signatures to the stable signatures identified across all folds, mean taxa retention was 83.3% for bovine and 91.7% for canine (Results, Lines 168–169). Regarding LODO machine learning results: per-fold signature sets comprised only 20–39 (9–18%) of the full 214 bovine genera and 13 (13%) of the full 98 canine genera (Table S3). As reported in Results (Lines 197–206):

"Despite this compactness, prediction performance on held-out study was comparable to models trained on the full feature set (four bovine diarrhea studies: 69.0%, 68.7%, 72.2%, 65.5% vs. 75.1%, 73.4%, 64.3%, 65.4%; three canine IBD studies: 73.2%, 75.7%, 76.6% vs. 73.2%, 75.0%, 67.4%; mean area under the receiver operating characteristic curve (AUC) across five classifiers), indicating the identified taxa capture the majority of the disease-discriminative signal. Feature importance scores within full-feature models further supported this, with signature genera consistently assigning higher importance than non-signature genera across studies and classifiers (Wilcoxon signed-rank test, $p < 0.05$; Figure S5)."

Point 2.4: Strange Random Forest setup = 20 trees to avoid overfitting is not justified.

Authors use RF with 20 trees and justify it by avoiding overfitting. This is unusual. RF typically becomes more stable with more trees; overfitting is not controlled by using an extremely small number of trees, but rather by depth and min-samples and proper validation. With 20 trees results can be unstable and sensitive to random seed.

Please tune RF hyperparameters properly (nested CV) and show sensitivity of results to number of trees. If the signature set changes then it is not robust.

A: The fixed 20-tree RF has been replaced with proper nested hyperparameter tuning via GridSearchCV within each LODO fold with inner three-fold stratified CV. The same strategy applies not only to RF but to all five classifiers. As described in Methods, "LODO machine learning for within-host cross-study validation" (Lines 498–506):

"Hyperparameters were tuned within each fold using inner three-fold stratified cross-validation (GridSearchCV, scoring = the area under the ROC curve (AUC)), searching over the following grids: RF (n_estimators $\in$ {50, 100, 200, 500, 1000}, max_depth $\in$ {None, 10, 20}, min_samples_leaf $\in$ {1, 3, 5}); LASSO and Ridge (C $\in$ {0.001, 0.01, 0.1, 1, 10, 100}); elastic net ($\alpha \in$ {0.0001, 0.001, 0.01, 0.1, 1}, l1_ratio $\in$ {0.15, 0.5, 0.85}); XGBoost (n_estimators $\in$ {50, 100, 200, 500, 1000}, max_depth $\in$ {3, 6}, learning_rate $\in$ {0.01, 0.1}). To address class imbalance, class weights were set to 'balanced' for LASSO, Ridge, elastic net, and RF, whereas XGBoost used scale_pos_weight = n_negative / n_positive. Performance was assessed on each held-out study using the AUC, sensitivity, and specificity." Furthermore, this nested hyperparameter tuning strategy is applied consistently across all modeling tasks in this study, including within-host cross-study (LODO ML and transfer learning), within-host cross-disease, within-host multi-disease, and cross-host validation.

The corresponding results are in Results (Lines 207–212):

"Hyperparameter sensitivity analysis across LODO folds showed that prediction performance was largely insensitive to the choice of hyperparameter values across all five classifiers (Figure S6), suggesting that the observed performance reflects genuine signal rather than model-tuning artifacts. These results demonstrate that the identified signatures capture generalizable diagnostic signals that remain robust across machine learning classifiers and hyperparameter settings, even across independent studies of the same host-disease combination despite considerable inter-study heterogeneity (Figures 2c–2i)."

Point 2.3: Batch correction in transfer learning may leak target-study information.

For multi-disease prediction authors state ComBat is applied within each CV iteration which is GOOD and correct. However in transfer learning sections it is stated that batch correction was performed "before model development" to assess transferability. This is not ok if ComBat parameters are estimated using any samples that later appear in the target test split. This is again leakage and can artificially increase cross-study transfer AUC.

Authors shall clarify and ensure that ComBat/MMUPHin fitting is always done on training data only and never using target-test samples.

A: Thank you for raising this point. We confirm that ComBat parameters were indeed estimated from training data only and never using target-test samples in our implementation. We acknowl-

edge that the original description was ambiguous and may have caused confusion. In the revised manuscript, we have clarified this explicitly in both the transfer learning and multi-disease prediction sections to avoid any confusion.

In Methods, "LODO transfer learning for within-host cross-study validation" (Lines 517–529):

"For each LODO fold, the target study was split into 75% and 25% subsets using stratified random sampling, repeated 20 times with different random seeds. All three strategies were evaluated on the identical 25% test set within each repeat. [...] The LODO transfer with ComBat strategy followed the same procedure, but ComBat batch correction was only applied to the source training data prior to pre-training."

In Methods, "Within-host multi-disease prediction" (Lines 551–553):

"To mitigate batch effects, ComBat correction [71] was applied to each training partition using study identity as the batch variable, with the resulting correction parameters then applied to the corresponding held-out test fold to prevent data leakage."

Reviewer #3

The authors present a well-structured and informative study that systematically investigates fecal microbiome signatures associated with intestinal diseases in domestic mammals, using cross-study integration alongside multi-disease and cross-host validation to assess the robustness and transferability of these signals. The abstract effectively communicates the overall motivation within a One Health framework and outlines a coherent analytical strategy to address study heterogeneity and batch effects. Overall, the work appears carefully executed, and the study design is logically aligned with the stated objectives. While the main message is clear, several points could be refined to improve readability and to better delineate the scope and interpretation of the conclusions.

A: We thank the reviewer for the positive assessment. As suggested, we have clarified the rationale for selecting bovine diarrhea and canine IBD for meta-analysis and for the choice of machine learning models at each validation stage. We have also improved figure readability through a more detailed workflow figure (Figure 1), increased spacing between panels, and consistent positioning of panel index labels across all figures. Additionally, we performed a careful proofreading pass to correct abbreviations, number formatting, and typographical inconsistencies throughout the manuscript.

Major Comments

Comment 3.1: Could the authors clarify the rationale for focusing the meta-analysis on canine and bovine cohorts? For example, it would be helpful to understand whether this choice

was driven primarily by data availability, study comparability, clinical relevance, geographic representation, or an a priori hypothesis about cross-host transferability.

A: The selection was driven by a combination of data availability, clinical relevance, and biological rationale. Among 31 retained studies from our systematic search, only bovine (calf) diarrhea and canine IBD had sufficient independent 16S studies (four and three, respectively) to support a LODO meta-analysis; no other host-disease combination had more than two studies. Beyond data availability, these two conditions represent prevalent intestinal diseases in livestock and companion animals, and their contrasting digestive physiologies (ruminant vs. monogastric) facilitate exploration of both host-specific and conserved dysbiosis patterns. We clarified this rationale in Introduction (Lines 96–105):

"Given the limited sample sizes, high heterogeneity, technical variability, and batch effects in existing case–control microbiome studies of domestic mammals (Table S1), pooling all data for joint analysis and modeling remains challenging. To address this, we first conducted two meta-analyses on 521 fecal 16S samples from four bovine (calf) diarrhea and three canine IBD studies to define genus-level microbial signatures of intestinal disease. These two host-disease combinations were selected based on the availability of multiple comparable case-control 16S rRNA studies (Tables S1-S2 and Figure 2a), their clinical importance as prevalent intestinal conditions in livestock and companion animals respectively [24,25], and their contrasting digestive physiologies as a ruminant herbivore and a monogastric companion animal [26], which together facilitate exploration of both host-specific and conserved dysbiosis patterns."

Comment 3.2: The manuscript appears to apply a neural network approach (like a deep neural network; DNN) alongside other classifiers. Could the authors briefly explain why this model family was selected for the present task and what advantages were expected in this setting? Making this motivation more explicit in the Methods and/or Introduction may help readers interpret the modeling choices.

A: We clarified the rationale for model selection at three points in the revised Methods, each tailored to the specific validation task:

For LODO machine learning (Methods, "LODO machine learning for within-host cross-study validation," Lines 489–493): "Five machine learning models were implemented using scikit-learn (v 1.6.1) [68] and XGBoost (v 3.2.0) [69] in Python 3.11: logistic regression with L1 regularization (LASSO), logistic regression with L2 regularization (Ridge), elastic net (SGDClassifier with combined L1/L2 penalty), random forest (RF), and gradient boosting (XGBoost). These models were selected for their established performance on high-dimensional microbiome data and interpretability [35]."

For transfer learning (Methods, "LODO transfer learning for within-host cross-study validation," Lines 507–512): "We implemented a transfer learning framework (Fig 1) using multilayer perceptron (MLP) classifiers (scikit-learn MLPClassifier [68]), applying the same per-fold signatures as the

LODO pipeline. Neural networks were chosen as they support weight-based fine-tuning, enabling pre-trained models to adapt to a new dataset via continued gradient descent [70]."

For cross-disease validation (Methods, "Within-host cross-disease validation," Lines 538–540): "RF and MLP were selected for cross-disease evaluation as they represent complementary learning paradigms."

For multi-disease prediction (Methods, "Within-host multi-disease prediction," Lines 549–551): "Model tuning and evaluation followed the same nested five × five stratified repeated cross-validation framework with inner three-fold hyperparameter tuning using the same search spaces."

For cross-host validation (Methods, "Cross-host intestinal validation," Lines 562–564): "The model configurations, and nested five × five stratified repeated cross-validation with inner three-fold hyperparameter tuning followed the same procedure as within-host cross-disease validation."

Minor Comments

Comment 3.3: Throughout the manuscript, there appear to be several minor issues such as omitted abbreviations, typographical errors, and formatting inconsistencies. A careful proofreading pass to correct these points would improve overall clarity and readability. (e.g. Page 4, Line 113 & 115: 3 → three; Page 6, Line 205: AUC; Page 8, Line 258: AUPR; etc.)

A: Thanks. We performed a thorough proofreading pass. All abbreviations are now defined at first use, and formatting inconsistencies have been corrected throughout. Additionally, numbers less than 10 are now spelled out (e.g., "three") unless they appear as part of a fraction (e.g., 1/3), are paired with numbers greater than 10 in the same sentence, or occur in a statistical/quantitative context.

Comment 3.4: Figure 1: It may improve clarity if the authors more explicitly indicate how datasets are partitioned (e.g., within-study vs cross-study splits; train/validation/test) and where batch correction is applied relative to data splitting and model training. A small annotation or step numbering could help readers follow the workflow.

A: Figure 1 has been substantially redesigned to address all of these points. The updated figure is organized into four clearly delineated analytical tiers with explicit annotations:

1. **LODO meta-analysis** (top panel): illustrates the per-study Hedges' g estimation, DerSimonian–Laird random-effects pooling, and stable signature identification across LODO folds.
2. **Within-host cross-study validation** (second panel): shows the LODO machine learning (Strategy A) and transfer learning (Strategy B) framework, with train/test splits explicitly labeled (100% source pre-train, 75% target fine-tune, 25% target test, repeated 20 times), and ComBat application annotated as applied only to source training data ("w/wo ComBat").

3. **Within-host cross-disease validation** (third panel): shows both cross-disease prediction (Strategy A) and multi-disease prediction (Strategy B) using nested cross-validation (5×5 outer, 3-fold inner), with the 80%/20% train/test split and ComBat application within each training partition explicitly labeled ("Train (ComBat)").
4. **Cross-host intestinal validation** (bottom panel): shows 80%/20% train/test splits per host dataset using nested cross-validation (5×5 outer, 3-fold inner), with stable taxa across LODO as feature sets.

The revised Figure 1 caption reads: "The framework encompasses four analytical tiers: (1) deriving stable disease-associated microbial signatures via leave-one-dataset-out (LODO) nested meta-analysis; (2) assessing cross-study reproducibility through LODO machine learning and transfer learning; (3) evaluating cross-disease specificity by applying signatures to independent intestinal and non-intestinal disease datasets within the same host; and (4) testing cross-host generalizability by applying bovine and canine signatures to other domestic mammal and human gut microbiome datasets."

To further improve readability, the titles of Figures 3–5 were also aligned with the pipeline tiers in Figure 1:

- **Figure 3.** Meta-analysis-derived disease signatures for bovine diarrhea and canine IBD, and within-host cross-study validation.
- **Figure 4.** Within-host cross-disease validation of bovine diarrhea and canine IBD-associated signatures.
- **Figure 5.** Cross-host intestinal validation of bovine diarrhea and canine IBD-associated signatures.

The revised Figure 1 is included at the end of this letter.

Comment 3.5: Figure 2: The overall readability could be improved. In particular, creating a clearer visual separation between panels D and E could help. For panels F–I, placing index labels in a consistent position (e.g., aligned on the left) could improve uniformity and make the figure easier to scan.

A: Figure 2 has been redesigned with improved spacing between panels, consistent upper-left positioning of panel labels, and clearer visual separation. The revised Figure 2 is included at the end of this letter.

Comment 3.6: Page 5, Lines 170–178 & Figure 3: It may be helpful to align the order of panel descriptions with the panel order (e.g., by swapping the order of 3B and 3C, or adjusting the narrative accordingly) to improve coherence. In addition, several text elements appear to overlap within the figure; reducing overlap and improving spacing would enhance legibility.

A: We aligned the narrative to follow the panel order sequentially. Figure 3 has been redesigned

with improved spacing and font sizing to eliminate text overlap. The revised Figure 3 is included at the end of this letter.

Comment 3.7: Page 6, Lines 206–208: Referring to "studies 1, 2, 3" may be slightly difficult to parse. Using a more explicit and consistent convention (e.g., "Study (1)", "Study (2)", "Study (3)" or "Study_1/ Study_2/ Study_3") may improve readability, especially if it matches the figure labels.

A: We adopted the "Study_1/Study_2/Study_3/Study_4" convention throughout the manuscript text and all figures for the meta-analysis studies to avoid confusion.

Comment 3.8: Figure 4A: The overall pipeline may be difficult to follow immediately. Could the authors consider making the directionality and sequence of steps more explicit (e.g., clearer arrows) to help readers understand the processing flow?

A: The original Figure 4A has been replaced by the redesigned Figure 1. The LODO transfer learning pipeline is now shown in the second panel with explicit directional arrows. For each LODO fold, repeated 20 times, the held-out study is split into 75% fine-tune and 25% test. The figure shows two parallel paths from the DNN pre-trained on source studies (Study 1 to Study $n-1$): (1) the cross-study model applies the pre-trained DNN directly to the 25% test set without fine-tuning; (2) the transfer model fine-tunes the pre-trained DNN on the 75% fine-tune subset of the held-out study, then evaluates on the same 25% test set (w/wo ComBat). The directional arrows and data flow are now clearly labeled at each step.

Additionally, to further improve the readability of the pipeline for readers, we reorganized the Methods section to follow the flow of Figure 1, with explicit sub-headings for each step:

- *Meta-analysis for bovine diarrhea and canine IBD* (Lines 446–477), including: Compositional data transformation; Per-study effect size estimation; Leave-one-dataset-out stability selection with random-effects meta-analysis; Sensitivity analysis.
- *Machine learning models for validation* (Lines 478–565), including:
  - LODO machine learning for within-host cross-study validation (Lines 485–506)
  - LODO transfer learning for within-host cross-study validation (Lines 507–530)
  - Within-host cross-disease validation (Lines 531–542)
  - Within-host multi-disease prediction (Lines 543–555)
  - Cross-host intestinal validation (Lines 556–565)

Reviewer #4

Comment 4.1: Lin et al. tackle a highly significant problem in this study, and their methodological approach is sound. My primary concern lies with the limited sample size, which is a critical consideration for a meta-analysis and makes it challenging to fully assess the robustness of the findings. The study's impact would be greatly enhanced by incorporating a larger and more diverse set of datasets to validate the proposed disease-associated signatures.

A: We share this concern and addressed it from two aspects.

First, we extended the literature search cutoff from February 2025 to February 2026 (Methods, "Study collection," Lines 386–400), which increased the total number of retained studies from 19 to 26. This resulted in expanded datasets for both meta-analysis and validation:

1. We added a fourth bovine diarrhea study (Zhang et al., 2025 [31]), increasing the meta-analysis from 461 to 521 samples (310 bovine + 211 canine; Results, Lines 118–120).
2. We expanded both cross-disease and cross-host validation. For cross-disease, we now include six bovine (two independent diarrhea cohorts [58,59], *Salmonella* infection [72], mastitis [73], low body condition score [74], and hyperketonemia [54]) and seven canine (*parvovirus* infection [75], two chronic enteropathy cohorts [76,60], two epilepsy cohorts [77,78], leishmaniasis [79], and hyperadrenocorticism [80]) validation datasets (Methods, Lines 531–537). For cross-host, we now include equine [81], feline (two diarrhea cohorts) [82,83], caprine [84], swine [85,61], and human (seven MicrobiomeHD datasets [27]) (Methods, Lines 556–562). Overall, the total number of validation datasets increased from 13 to 19 (46% increase), and total validation samples increased from 1,464 to 2,082 (42% increase) (Tables S1–S2).

Second, we explicitly acknowledge the sample size limitation at multiple points in the revised manuscript:

- Discussion (Lines 328–330): "these findings should be interpreted with caution, as the limited number of studies and samples, inter-study heterogeneity, and variation in disease severity and sequencing depth may mask genuine disease signals as false negatives or amplify study-specific effects as false positives."
- Discussion (Lines 338–340): "These limitations call for large-scale, standardized, well-annotated, prospectively collected datasets to disentangle disease-specific from confounding signals in animal microbiome meta-analysis."
- Discussion (Lines 346–350): "At the cross-study level, transfer learning substantially improved generalization over direct model application, consistent with evidence from human cohorts that pretraining and fine-tuning can borrow disease-relevant microbial knowledge to aid diagnosis in data-limited settings [48], a strategy particularly valuable in animal microbiome research where data scarcity remains a persistent constraint."
- Discussion (Lines 364–366): "Increasing the number of independent studies per host-disease combination through coordinated multi-site data collection would also strengthen LODO-based stability assessment and cross-study generalization."

- Discussion (Lines 377–383): “In summary, although this study is constrained by limited animal fecal microbiome datasets, genus-level taxonomic resolution, and an observational design that precludes causal inference, the pipeline integrating leave-one-dataset-out meta-analysis with multi-layered cross-study, cross-disease, and cross-host validation yields robust intestinal disease-associated signatures in domestic mammals, though the generalizability of the pipeline, the practical utility of these signatures across clinical contexts, and the underlying biological mechanisms all remain subjects for future investigation.”

We also note in Results (Lines 264–268) that multi-disease prediction is currently limited to canine due to dataset availability: “The similar pattern in AUPR is present in Figure S8b. These results demonstrate that a reduced set of intestinal disease-associated taxa retains multi-class discriminative power, supporting potential utility in clinical screening for canine diseases, with analogous multi-disease prediction in bovine warranting future exploration as appropriate datasets become available.”

Revised Figures

Figure 1. Predictive modeling workflow using publicly available fecal microbiome databases of domestic mammals under a case–control design. The framework encompasses four analytical tiers: (1) deriving stable disease-associated microbial signatures via leave-one-dataset-out (LODO) nested meta-analysis; (2) assessing cross-study reproducibility through LODO machine learning and transfer learning; (3) evaluating cross-disease specificity by applying signatures to independent intestinal and non-intestinal disease datasets within the same host; and (4) testing cross-host generalizability by applying bovine and canine signatures to other domestic mammal and human gut microbiome datasets.

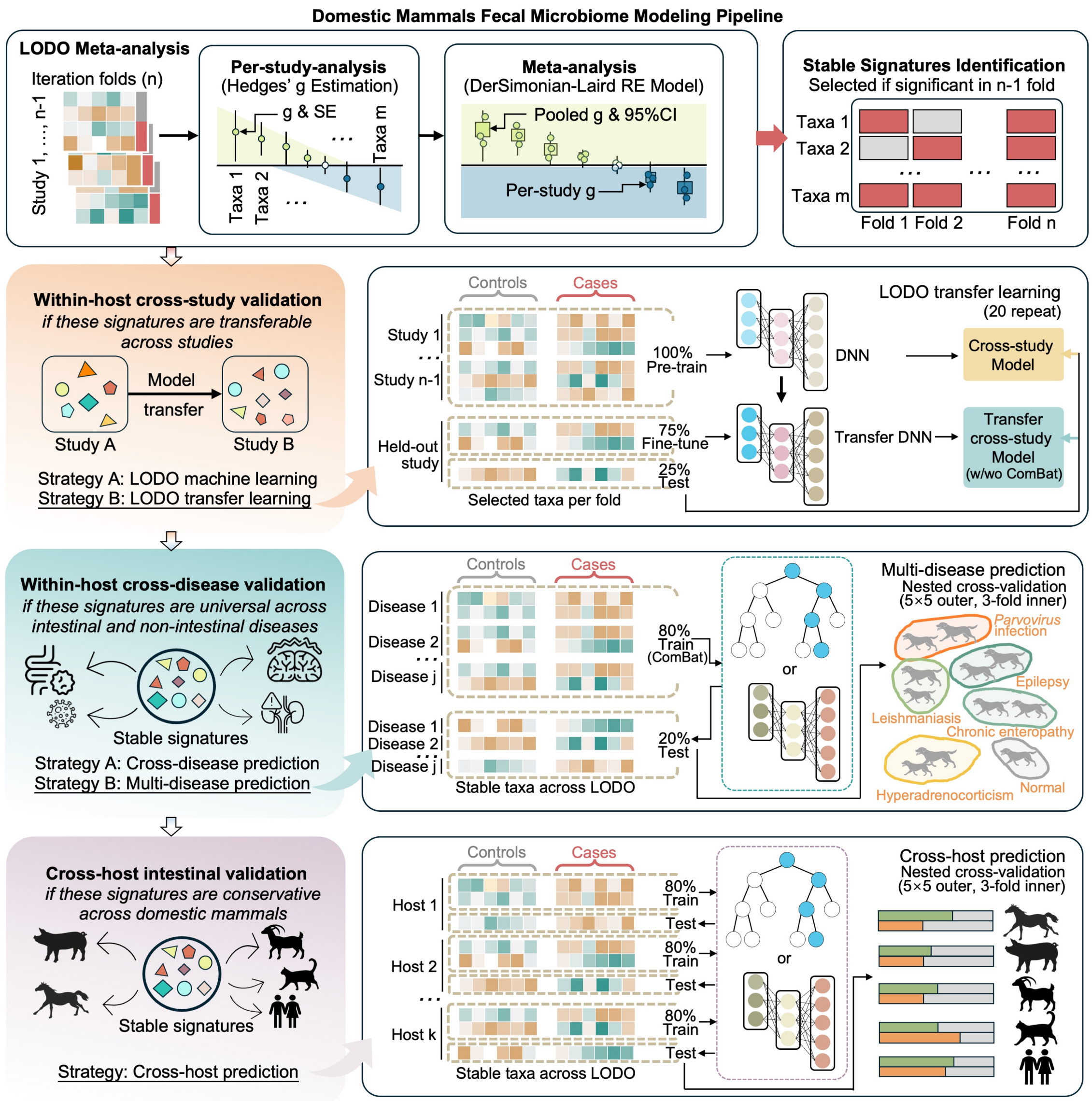

Figure 2. Dataset overview. a. Overview of all fecal microbiome case–control datasets included in this study, organized by host species phylogeny. A total of 26 studies across 6 domesticated mammals (803 controls and 618 cases) were retrieved from public databases. Studies used for bovine and canine meta-analysis are numbered (1–4 and 1–3, respectively); remaining datasets served as within-host intestinal, within-host non-intestinal, or cross-host intestinal validation. The phylogenetic tree topology was retrieved from O'Leary et al., Science (2023) [89]. b. Sampling sites, sex and host age distributions for the four bovine diarrhea (top) and three canine IBD (bottom) meta-analysis studies. c. Mean phylum-level relative abundance of fecal microbiota in case and control groups across the four bovine and three canine meta-analysis studies. d. Effect size (R^2) of case–control status on genus-level microbiome composition in each meta-analysis study, assessed by PERMANOVA on Bray–Curtis distances (999 permutations; $*p < 0.05$, $**p < 0.01$, $***p < 0.001$). e. Log_2 fold change (case/control) in fecal microbial alpha diversity (Shannon index) for each meta-analysis study, assessed by Wilcoxon rank-sum test ($*p < 0.05$, $***p < 0.001$). f, g. Metric multidimensional scaling (MDS) of Bray–Curtis distances for the four bovine (f) and three canine (g) meta-analysis studies. Each study is represented by a 95% confidence ellipse; boxplots depict sample distributions along each axis. Inset PERMANOVA R^2 values quantify the contributions of study origin, case–control status, sequencing platform, and geographic location to microbiome variation. h, i. MDS plots after removing the effects of study origin, sequencing platform, and geographic location via distance-based redundancy analysis (dbRDA), revealing residual case–control separation in bovine (h) and canine (i) cohorts. Each group is represented by a 95% confidence ellipse; boxplots depict sample distributions along each axis.

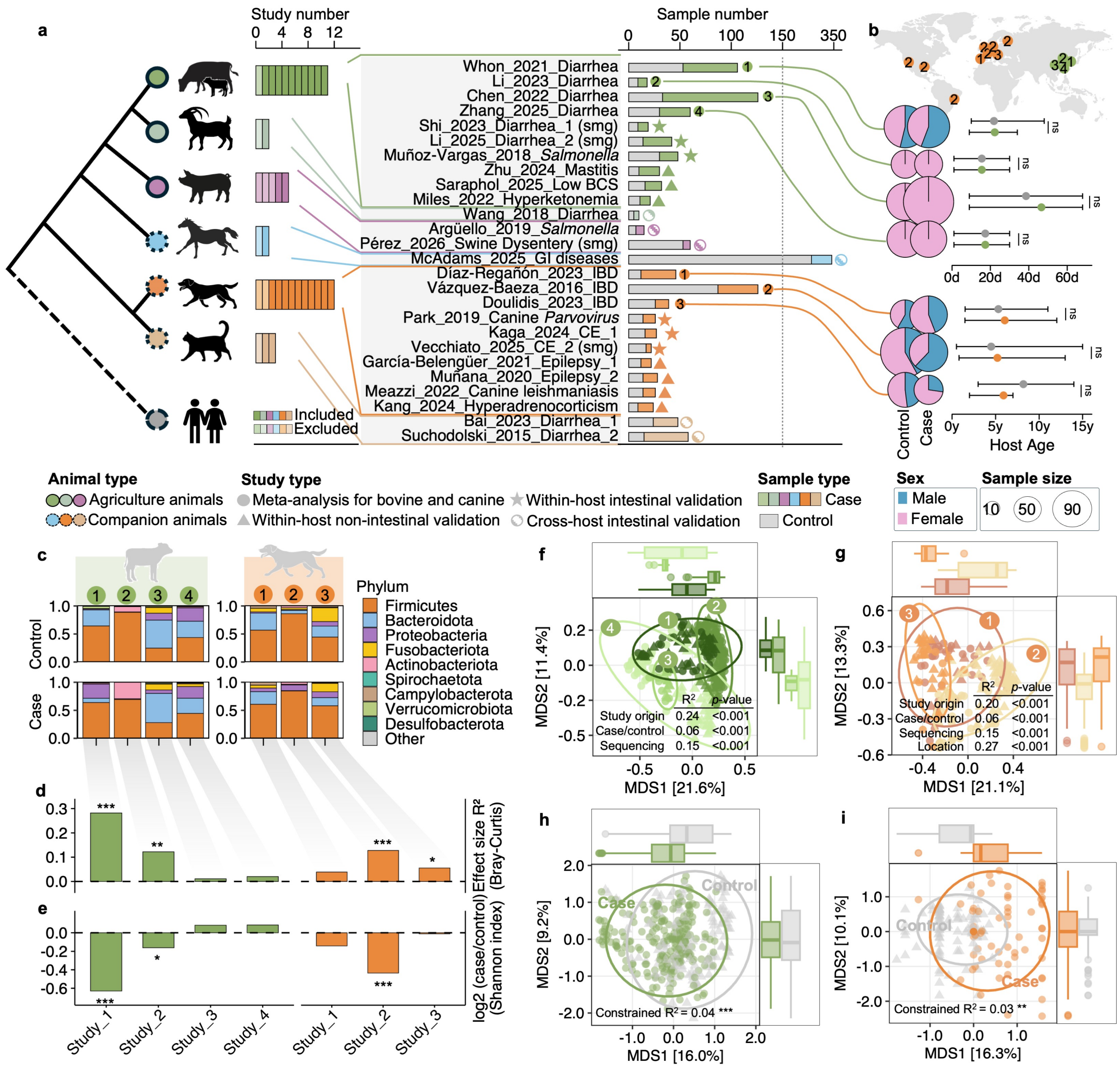

a
Study number
Sample number
Whon_2021_Diarrhea
Li_2023_Diarrhea
Chen_2022_Diarrhea
Zhang_2025_Diarrhea
Shi_2023_Diarrhea_1 (smg)
Li_2025_Diarrhea_2 (smg)
Muñoz-Vargas_2018_Salmonella
Zhu_2024_Mastitis
Saraphol_2025_Low BCS
Miles_2022_Hyperketonemia
Wang_2018_Diarrhea
Argüello_2019_Salmonella
Pérez_2026_Swine Dysentery (smg)
McAdams_2025_GI diseases
Díaz-Regañón_2023_IBD
Vázquez-Baeza_2016_IBD
Doulidis_2023_IBD
Park_2019_Canine Parvovirus
Kaga_2024_CE_1
Vecchiato_2025_CE_2 (smg)
García-Belengüer_2021_Epilepsy_1
Muñana_2020_Epilepsy_2
Meazzi_2022_Canine leishmaniasis
Kang_2024_Hyperadrenocorticism
Bai_2023_Diarrhea_1
Suchodolski_2015_Diarrhea_2
Included
Excluded
b
Control
Case
Host Age
Animal type
Agriculture animals
Companion animals
Study type
Meta-analysis for bovine and canine
Within-host non-intestinal validation
Within-host intestinal validation
Cross-host intestinal validation
Sample type
Case
Control
Sex
Male
Female
Sample size
c
Control
Case
Phylum
Firmicutes
Bacteroidota
Proteobacteria
Fusobacteriota
Actinobacteriota
Spirochaetota
Campylobacterota
Verrucomicrobiota
Desulfobacterota
Other
d
Effect size R² (Bray-Curtis)
e
log2 (case/control) (Shannon index)
Study_1
Study_2
Study_3
Study_4
f
MDS1 [21.6%]
MDS2 [11.4%]
Study origin 0.24 <0.001
Case/control 0.06 <0.001
Sequencing 0.15 <0.001
g
MDS1 [21.1%]
MDS2 [13.3%]
Study origin 0.20 <0.001
Case/control 0.06 <0.001
Sequencing 0.15 <0.001
Location 0.27 <0.001
h
Case
Control
Constrained R² = 0.04 ***
MDS1 [16.0%]
MDS2 [9.2%]
i
Control
Case
Constrained R² = 0.03 **
MDS1 [16.3%]
MDS2 [10.1%]

Figure 3. Meta-analysis-derived disease signatures for bovine diarrhea and canine IBD, and within-host cross-study validation. a. Leave-one-dataset-out (LODO) nested meta-analysis results for bovine diarrhea (top, 4 studies) and canine IBD (bottom, 3 studies). Forest plots show the pooled Hedges' g effect size across folds (median, large dot; per-study g, small dots) with 95% CI for each signature genus; orange and blue backgrounds indicate case enrichment and depletion, respectively. The heatmap color reflects the $\log_2$(case/control) abundance ratio, and per-study FDR-corrected significance is indicated (*$p < 0.10$, **$p < 0.01$, ***$p < 0.001$). Illustrations of the calf, dog, gastrointestinal tract, and microbiome were created with BioRender. b. Within-host cross-study classification performance (AUC) of five machine learning classifiers (LASSO, Ridge, Elastic Net, Random Forest, XGBoost) trained on signature genera (reduced set, triangles) or all genera (full set, circles) under the LODO nested cross-validation framework, evaluated on each held-out study for bovine (top) and canine (bottom). c. Comparison of three cross-study transfer strategies (LODO cross-validation, LODO transfer learning, and LODO transfer learning with ComBat batch correction) for bovine (left) and canine (right). AUC distributions across 20 repeated runs are shown as violin plots with embedded boxplots, with individual repeat values overlaid as points. FDR-corrected pairwise comparisons: *$p < 0.10$, **$p < 0.01$, ***$p < 0.001$.

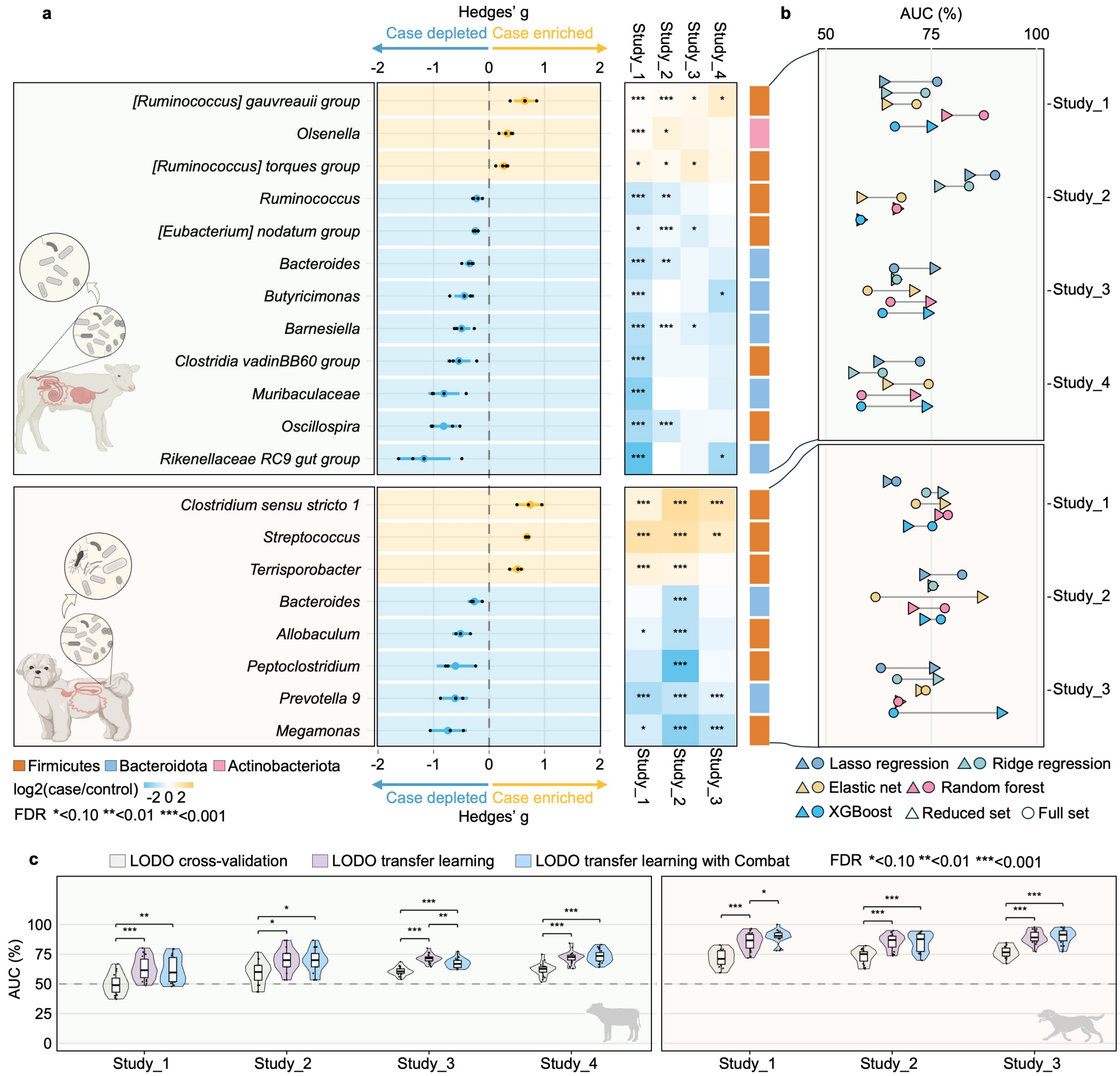

a
Hedges' g
Case depleted
Case enriched
[Ruminococcus] gauvreauii group
Olsenella
[Ruminococcus] torques group
Ruminococcus
[Eubacterium] nodatum group
Bacteroides
Butyricimonas
Barnesiella
Clostridia vadinBB60 group
Muribaculaceae
Oscillospira
Rikenellaceae RC9 gut group
Clostridium sensu stricto 1
Streptococcus
Terrisporobacter
Bacteroides
Allobaculum
Peptoclostridium
Prevotella 9
Megamonas
Study_1
Study_2
Study_3
Study_4
Firmicutes
Bacteroidota
Actinobacteriota
log2(case/control)
FDR *<0.10 **<0.01 ***<0.001
b
AUC (%)
Lasso regression
Ridge regression
Elastic net
Random forest
XGBoost
Reduced set
Full set
c
LODO cross-validation
LODO transfer learning
LODO transfer learning with Combat
FDR *<0.10 **<0.01 ***<0.001
AUC (%)

Figure 4. Within-host cross-disease validation of bovine diarrhea and canine IBD-associated signatures. a, b. Binary classification performance (AUC) of Random Forest and Neural Network models using bovine diarrhea-associated (a) or canine IBD-associated (b) stable signature genera (reduced set, triangles) or all genera (full set, circles), evaluated across within-host intestinal (stars) and non-intestinal (filled triangles) validation datasets via 5 repeats $\times$ 5-fold cross-validation. Individual repeat values are shown as grey points. FDR-corrected significance: *$p < 0.10$, **$p < 0.01$, ***$p < 0.001$. c, d. Spearman correlation between the PERMANOVA effect size (R^2, Bray–Curtis distances, 999 permutations) of signature genera and classification AUC across bovine (c) and canine (d) validation datasets, for Random Forest and Neural Network models. e, f. MaAsLin2 association heatmaps showing the relationship between bovine diarrhea (e) or canine IBD (f) signature genera and disease status across all within-host validation datasets. Color reflects $-\log(\text{FDR}) \times \text{sign(coefficient)}$, indicating direction and significance of association; asterisks denote FDR-corrected significance (*$p < 0.10$, **$p < 0.01$, ***$p < 0.001$). g. Multi-class disease prediction performance (AUC per class) in canine, using the IBD signature genera to simultaneously distinguish six classes (chronic enteropathy, *parvovirus* infection, epilepsy, leishmaniasis, hyperadrenocorticism, and normal), evaluated by Random Forest and Neural Network with 5-fold stratified cross-validation $\times$ 5 repeats. Individual fold values are overlaid as points. The veterinarian and dog illustration was created with Storyset.

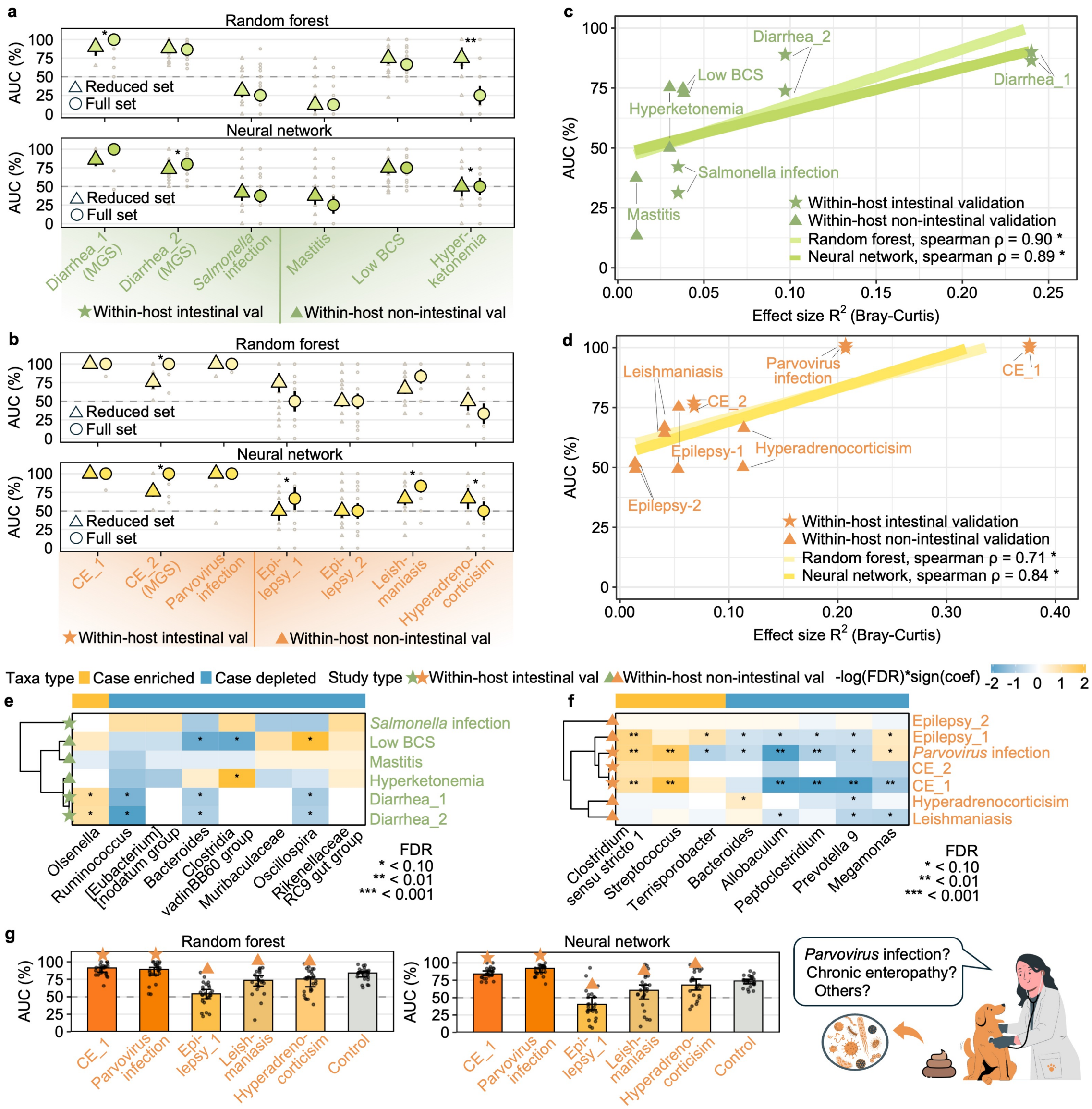
a
Random forest
AUC (%)
Reduced set
Full set
Neural network
Diarrhea_1 (MGS)
Diarrhea_2 (MGS)
Salmonella infection
Mastitis
Low BCS
Hyper-ketonemia
Within-host intestinal val
Within-host non-intestinal val
b
Random forest
Neural network
CE_1
CE_2 (MGS)
Parvovirus infection
Epi-lepsy_1
Epi-lepsy_2
Leish-maniasis
Hyperadreno-corticisim
c
Diarrhea_2
Low BCS
Hyperketonemia
Diarrhea_1
Salmonella infection
Mastitis
Within-host intestinal validation
Within-host non-intestinal validation
Random forest, spearman ρ = 0.90 *
Neural network, spearman ρ = 0.89 *
Effect size R2 (Bray-Curtis)
d
Leishmaniasis
Parvovirus infection
CE_1
CE_2
Epilepsy-1
Hyperadrenocorticisim
Epilepsy-2
Random forest, spearman ρ = 0.71 *
Neural network, spearman ρ = 0.84 *
Taxa type
Case enriched
Case depleted
Study type
-log(FDR)*sign(coef)
e
Olsenella
Ruminococcus
[Eubacterium] nodatum group
Bacteroides
Clostridia vadinBB60 group
Muribaculaceae
Oscillospira
Rikenellaceae RC9 gut group
FDR
* < 0.10
** < 0.01
*** < 0.001
f
Epilepsy_2
Epilepsy_1
CE_2
CE_1
Hyperadrenocorticisim
Leishmaniasis
Clostridium sensu stricto 1
Streptococcus
Terrisporobacter
Allobaculum
Peptoclostridium
Prevotella 9
Megamonas
g
Control
Parvovirus infection?
Chronic enteropathy?
Others?

Figure 5. Cross-host intestinal validation of bovine diarrhea and canine IBD-associated signatures. a. Schematic overview of the cross-host validation. Bovine diarrhea (green arrows) and canine IBD (orange arrows)-associated stable signatures were applied to fecal microbiome datasets from other domestic mammals (canine, equine, caprine, swine) and humans. Human intestinal disease datasets (CDI, IBD, and AEI) were retrieved from MicrobiomeHD; sample sizes are shown for each dataset. Illustrations of the human and gastrointestinal tract were created with BioRender. b. Cross-host classification performance (AUC) of Random Forest (top) and Neural Network (bottom) models, trained using bovine diarrhea-associated (green) or canine IBD-associated (orange) signature genera and evaluated on held-out datasets from each host species via 5 repeats $\times$ 5-fold cross-validation. Individual repeat values are overlaid as points. FDR-corrected pairwise comparisons between bovine and canine signatures: $*p < 0.10$, $**p < 0.01$, $***p < 0.001$. c. MaAsLin2 association heatmap of genera shared between bovine diarrhea and canine IBD-associated signatures across all cross-host validation datasets. Color reflects $-\log(\text{FDR}) \times \text{sign(coefficient)}$, indicating direction and significance of association; asterisks denote FDR-corrected significance ($*p < 0.10$, $**p < 0.01$, $***p < 0.001$). Top annotation bars indicate the signature source host and taxa type (case enriched or depleted). d. Log_2(case/control) abundance ratios of six genera shared between bovine/canine and human intestinal diseases.

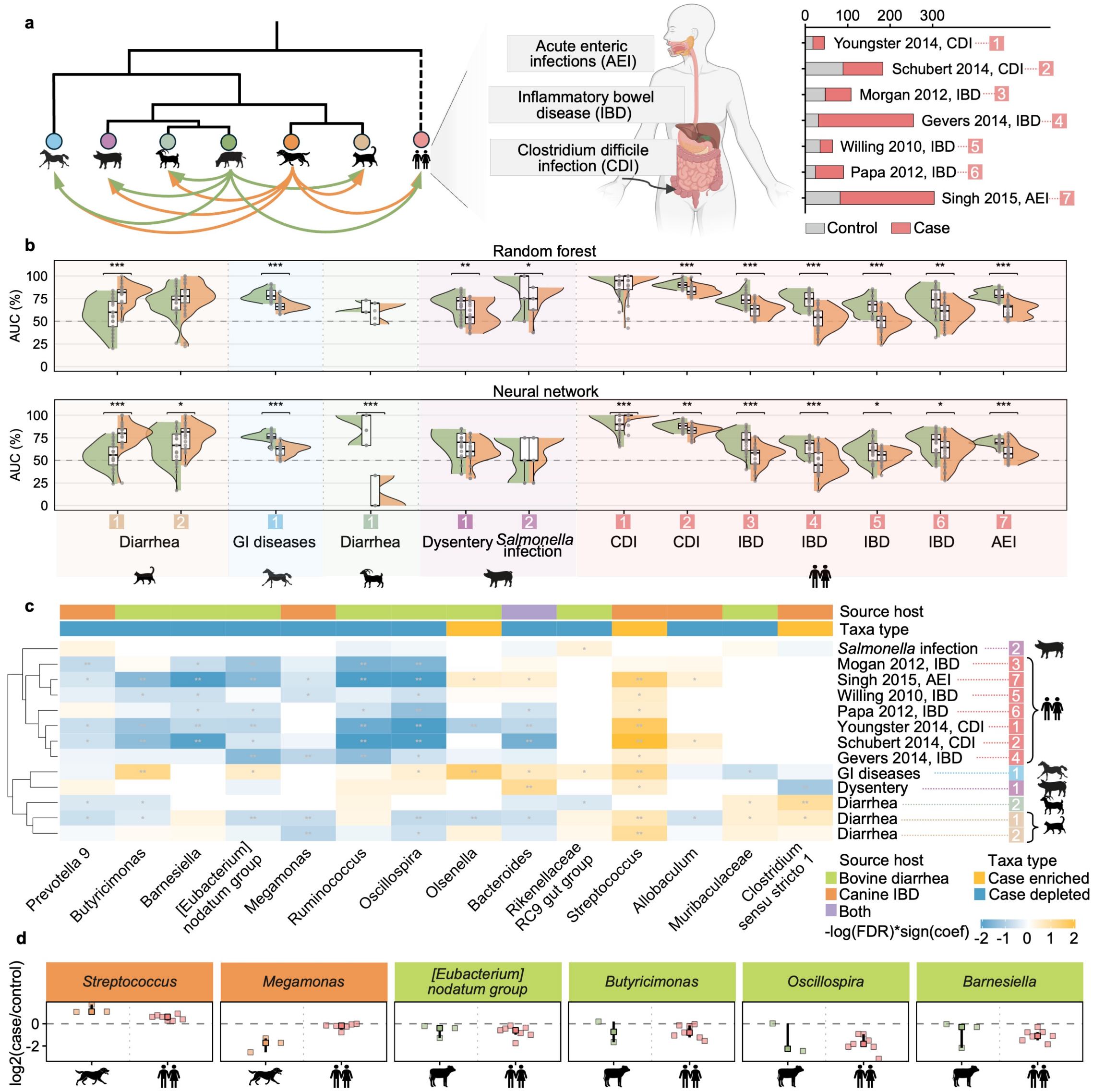
a
Acute enteric infections (AEI)
Inflammatory bowel disease (IBD)
Clostridium difficile infection (CDI)
0 100 200 300
Youngster 2014, CDI 1
Schubert 2014, CDI 2
Morgan 2012, IBD 3
Gevers 2014, IBD 4
Willing 2010, IBD 5
Papa 2012, IBD 6
Singh 2015, AEI 7
Control
Case
b
Random forest
Neural network
AUC (%)
Diarrhea
GI diseases
Diarrhea
Dysentery
Salmonella infection
CDI
CDI
IBD
IBD
IBD
IBD
AEI
c
Source host
Taxa type
Salmonella infection
Mogan 2012, IBD
Singh 2015, AEI
Willing 2010, IBD
Papa 2012, IBD
Youngster 2014, CDI
Schubert 2014, CDI
Gevers 2014, IBD
GI diseases
Dysentery
Diarrhea
Diarrhea
Diarrhea
Prevotella 9
Butyricimonas
Barnesiella
[Eubacterium] nodatum group
Megamonas
Ruminococcus
Oscillospira
Olsenella
Bacteroides
Rikenellaceae RC9 gut group
Streptococcus
Allobaculum
Muribaculaceae
Clostridium sensu stricto 1
Source host
Bovine diarrhea
Canine IBD
Both
Taxa type
Case enriched
Case depleted
-log(FDR)*sign(coef)
-2 -1 0 1 2
d
Streptococcus
Megamonas
[Eubacterium] nodatum group
Butyricimonas
Oscillospira
Barnesiella
log2(case/control)
0
-2

Point-by-Point Response to Reviewers

Manuscript: COMMSBIO-25-12342A

"Toward diseases-associated fecal microbiome signatures for domestic mammals"

We thank the editor and reviewers for reviewing our revised manuscript. The minor correction noted by Reviewer #2 regarding off-by-one supplementary figure references has been corrected. Below, we provide our point-by-point response.

Reviewer comments are shown in black and our responses are shown in blue.

Reviewer #2

The authors have redesigned the analytical pipeline in line with my critiques. The pooled Wilcoxon approach is replaced with a formal random-effects meta-analysis embedded in a leave-one-dataset-out framework. CLR-based compositional handling with a 16-combination sensitivity analysis is OK. Hyperparameter tuning is now proper and ComBat application to training data only is now explicitly stated. I recommend acceptance pending one minor correction: supplementary figure references on lines 387–388 are off-by-one (should be Figure S2 and Figure S3 respectively).

A: We thank the reviewer for the positive assessment of the revised pipeline. We have corrected the supplementary figure references as indicated (Results, Lines 168–173):

"The Leave-One-Dataset-Out (LODO)-nested meta-analysis identified 12 bovine and 8 canine stable taxa consistently altered across studies (Figure 3a; mean taxa retention across folds: 83.3% bovine, 91.7% canine; each fold's results in Supplementary Table 1 and Supplementary Table 2), corresponding to all taxa significant in the full pooled meta-analysis for bovine and eight of nine for canine (Supplementary Figure 2), and robust across CLR-based zero-handling parameterizations and FDR thresholds of 0.05–0.20 (Supplementary Figure 3)."

Reviewer #3

The authors have provided a meticulous response to the reviewers' comments. The revised manuscript is well-structured and significantly improved in terms of clarity and flow. I believe the paper is now well-suited for publication in its current form.

A: We thank the reviewer for the positive evaluation.

Reviewer #4

Thank you to the author for the revisions. After carefully reviewing the updated submission, I confirm that all my previous comments have been satisfactorily addressed. I have no further corrections or objections. Therefore, I recommend acceptance of the paper as is.

A: We thank the reviewer for confirming that all comments have been addressed.

Comments for the Authors

The authors present a well-structured and informative study that systematically investigates fecal microbiome signatures associated with intestinal diseases in domestic mammals, using cross-study integration alongside multi-disease and cross-host validation to assess the robustness and transferability of these signals. The abstract effectively communicates the overall motivation within a One Health framework and outlines a coherent analytical strategy to address study heterogeneity and batch effects. Overall, the work appears carefully executed and the study design is logically aligned with the stated objectives. While the main message is clear, several points could be refined to improve readability and to better delineate the scope and interpretation of the conclusions.

Major Comments

1. Could the authors clarify the rationale for focusing the meta-analysis on canine and bovine cohorts? For example, it would be helpful to understand whether this choice was driven primarily by data availability, study comparability, clinical relevance, geographic representation, or an a priori hypothesis about cross-host transferability.
2. The manuscript appears to apply a neural network approach (like a deep neural network; DNN) alongside other classifiers. Could the authors briefly explain why this model family was selected for the present task and what advantages were expected in this setting? Making this motivation more explicit in the Methods and/or Introduction may help readers interpret the modeling choices.

Minor Comments

1. Throughout the manuscript, there appear to be several minor issues such as omitted abbreviations, typographical errors, and formatting inconsistencies. A careful proofreading pass to correct these points would improve overall clarity and readability. (e.g. Page 4, Line 113 & 115: 3 → three; Page 6, Line 205: AUC; Page 8, Line 258:

AUPR; etc.)

2. Figure 1: It may improve clarity if the authors more explicitly indicate how datasets are partitioned (e.g., within-study vs cross-study splits; train/validation/test) and where batch correction is applied relative to data splitting and model training. A small annotation or step numbering could help readers follow the workflow.
3. Figure 2: The overall readability could be improved. In particular, creating a clearer visual separation between panels D and E could help. For panels F–I, placing index labels in a consistent position (e.g., aligned on the left) could improve uniformity and make the figure easier to scan.
4. Page 5, Lines 169–177 & Figure 3: It may be helpful to align the order of panel descriptions with the panel order (e.g., by swapping the order of 3B and 3C, or adjusting the narrative accordingly) to improve coherence. In addition, several text elements appear to overlap within the figure; reducing overlap and improving spacing would enhance legibility.
5. Page 6, Lines 205–207: Referring to "studies 1, 2, 3" may be slightly difficult to parse. Using a more explicit and consistent convention (e.g., "Study (1)", "Study (2)", "Study (3)" or "Study_1/ Study_2/ Study_3") may improve readability, especially if it matches the figure labels.
6. Figure 4A: The overall pipeline may be difficult to follow immediately. Could the authors consider making the directionality and sequence of steps more explicit (e.g., clearer arrows) to help readers understand the processing flow?
