# Supplementary material for: Toward disease-associated fecal microbiome signatures for domestic mammals
Source: Commun Biol. 2026 Jul 14;9:1474. doi: 10.1038/s42003-026-10647-5 (PMC13737101; doi:10.1038/s42003-026-10647-5)
Supplement: Supplementary file 2 [file 42003_2026_10647_MOESM2_ESM.pdf]

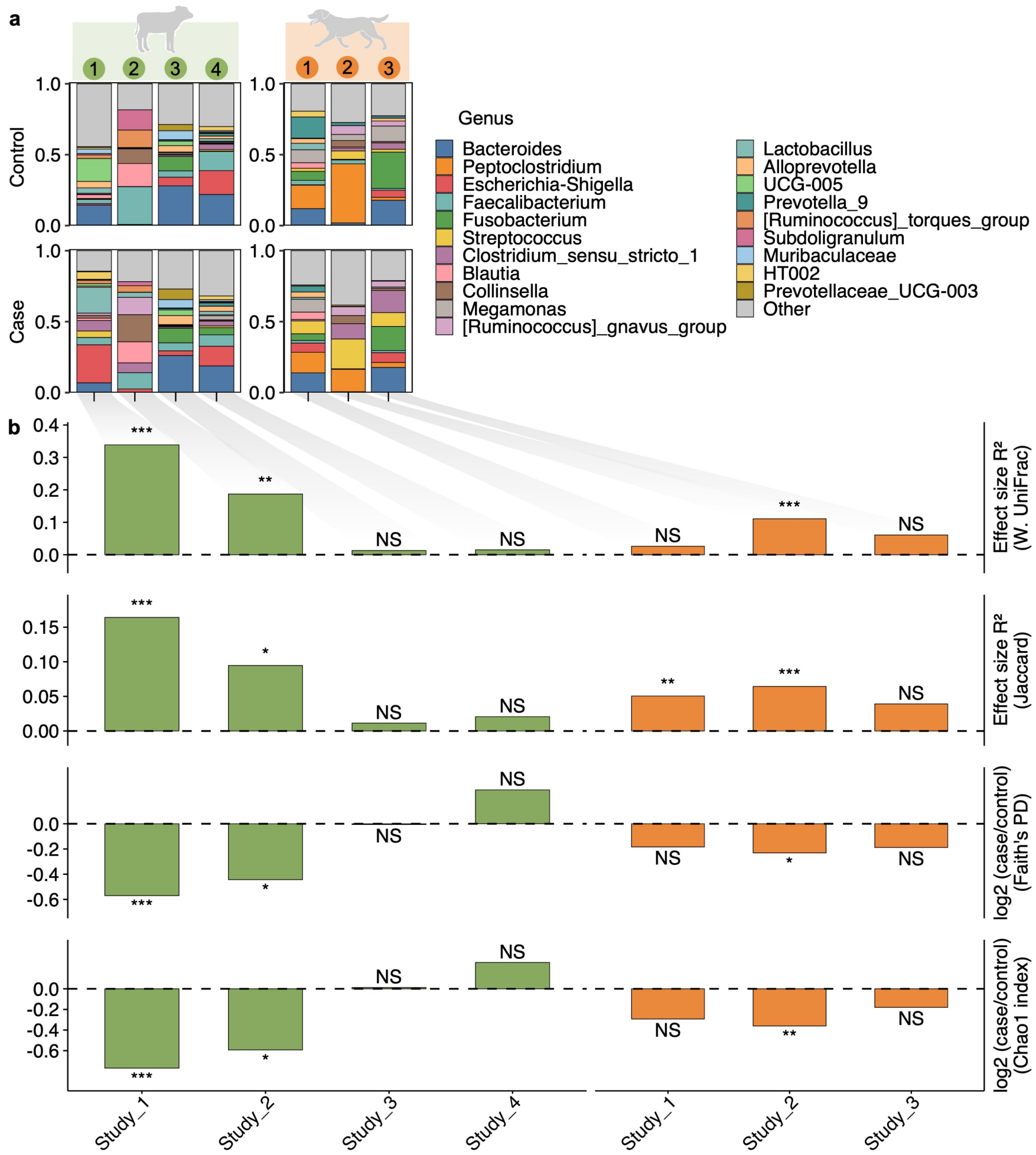

Supplementary Figure 1. Genus-level microbiome composition and diversity characteristics of the meta-analysis cohorts. (a) Mean genus-level relative abundance of fecal microbiota in control (top) and case (bottom) groups across the four bovine diarrhea and three canine IBD meta-analysis studies. The top 20 most abundant genera are shown individually; remaining genera are grouped as "Other". (b) Beta diversity effect size (R^2) assessed by PERMANOVA on weighted UniFrac (top) and Jaccard (second) distances (999 permutations), and $\log_2$ fold change (case/control) in alpha diversity measured by Faith's PD (third) and Chao1 index (bottom), for each meta-analysis study. Significance by Wilcoxon rank-sum test: NS, not significant; *$p < 0.05$, **$p < 0.01$, ***$p < 0.001$. Sample sizes (n biologically independent samples): bovine diarrhea meta-analysis: Study_1, n = 106 (53 controls, 53 cases); Study_2, n = 18 (9 controls, 9 cases); Study_3, n = 126 (33 controls, 93 cases); Study_4, n = 60 (30 controls, 30 cases); canine IBD meta-analysis: Study_1, n = 46 (12 controls, 34 cases); Study_2, n = 126 (87 controls, 39 cases); Study_3, n = 39 (26 controls, 13 cases).

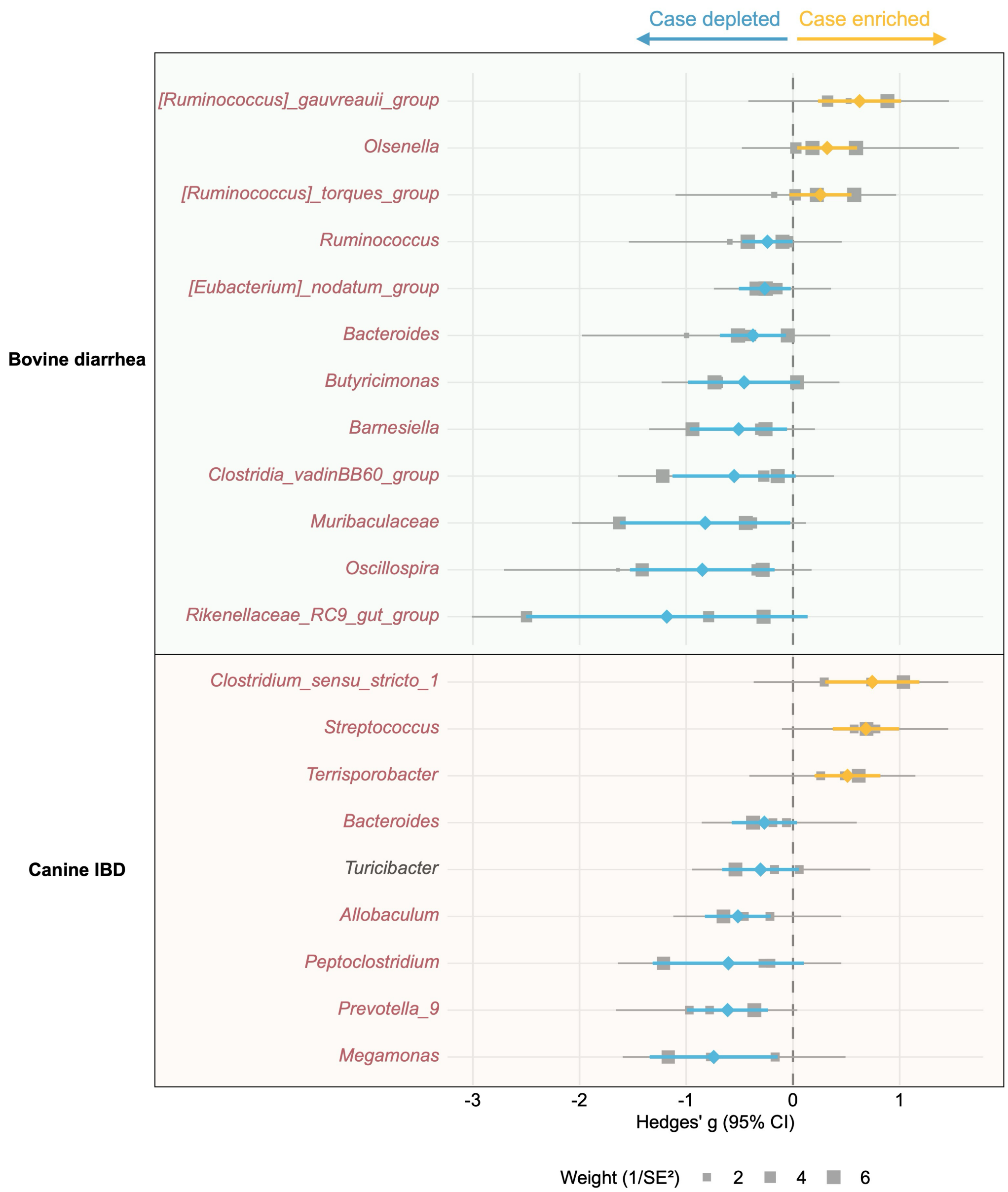

Supplementary Figure 2. Full meta-analysis forest plot for bovine diarrhea and canine IBD-associated signature genera. Forest plot showing the pooled Hedges'g effect size (diamond, 95% CI) for each signature genus derived from the full meta-analysis (all studies included) for bovine diarrhea (top) and canine IBD (bottom). Horizontal lines indicate 95% confidence intervals; square size reflects inverse-variance weight ($1/SE^2$) for each study. Yellow and blue colors indicate case enrichment and depletion, respectively.

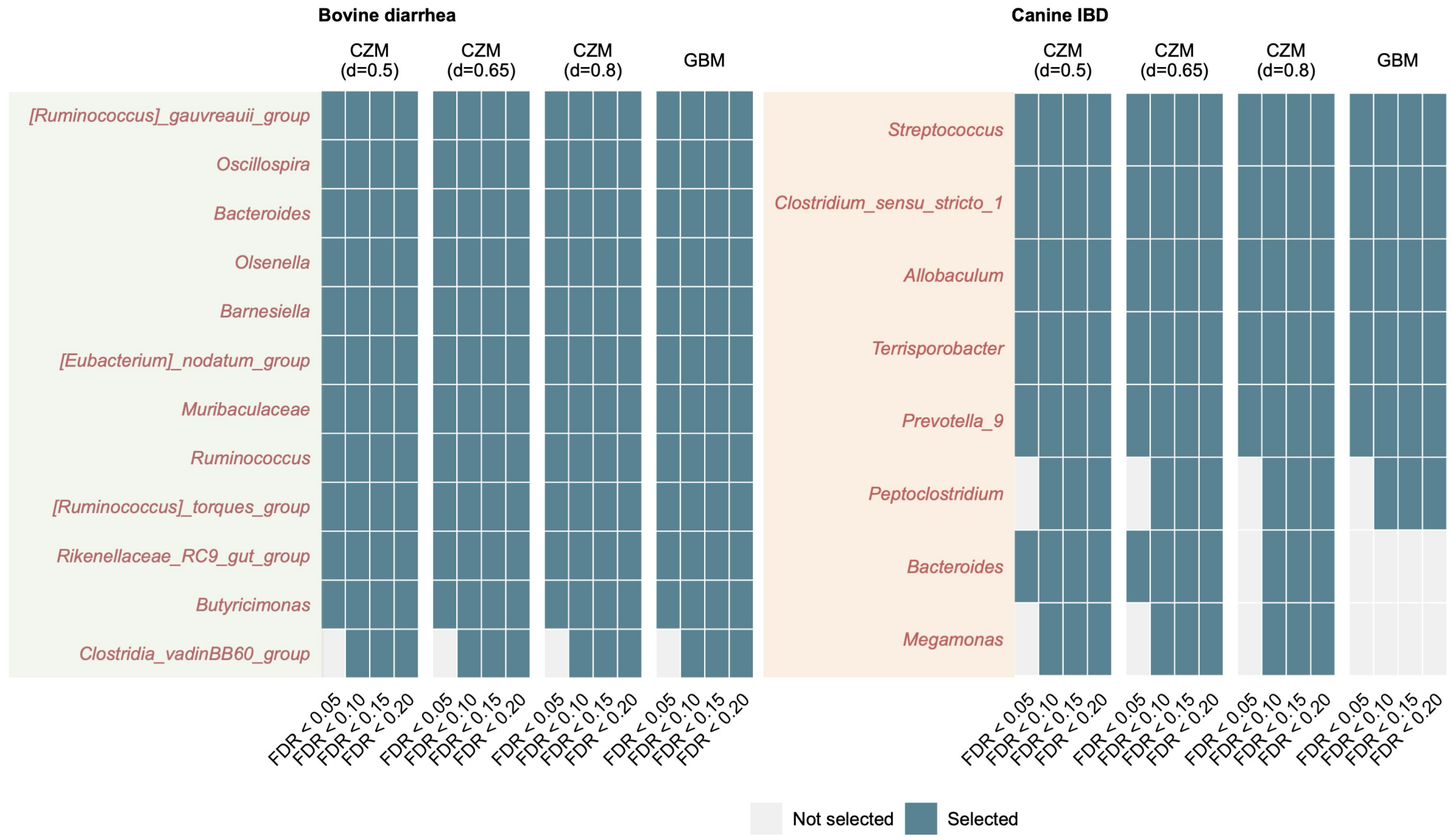

Supplementary Figure 3. Sensitivity analysis of signature genus selection across zero-replacement methods and FDR thresholds. Heatmap showing whether each candidate genus was selected (teal) or not selected (light grey) under different combinations of zero-replacement method and FDR threshold for bovine diarrhea (left) and canine IBD (right). Three count zero multiplicative (CZM) replacement fractions (d = 0.5, 0.65, 0.8) and a geometric Bayesian multiplicative (GBM) method were tested, each evaluated at four FDR thresholds (< 0.05, < 0.10, < 0.15, < 0.20). Genera are ordered by selection stability across conditions.

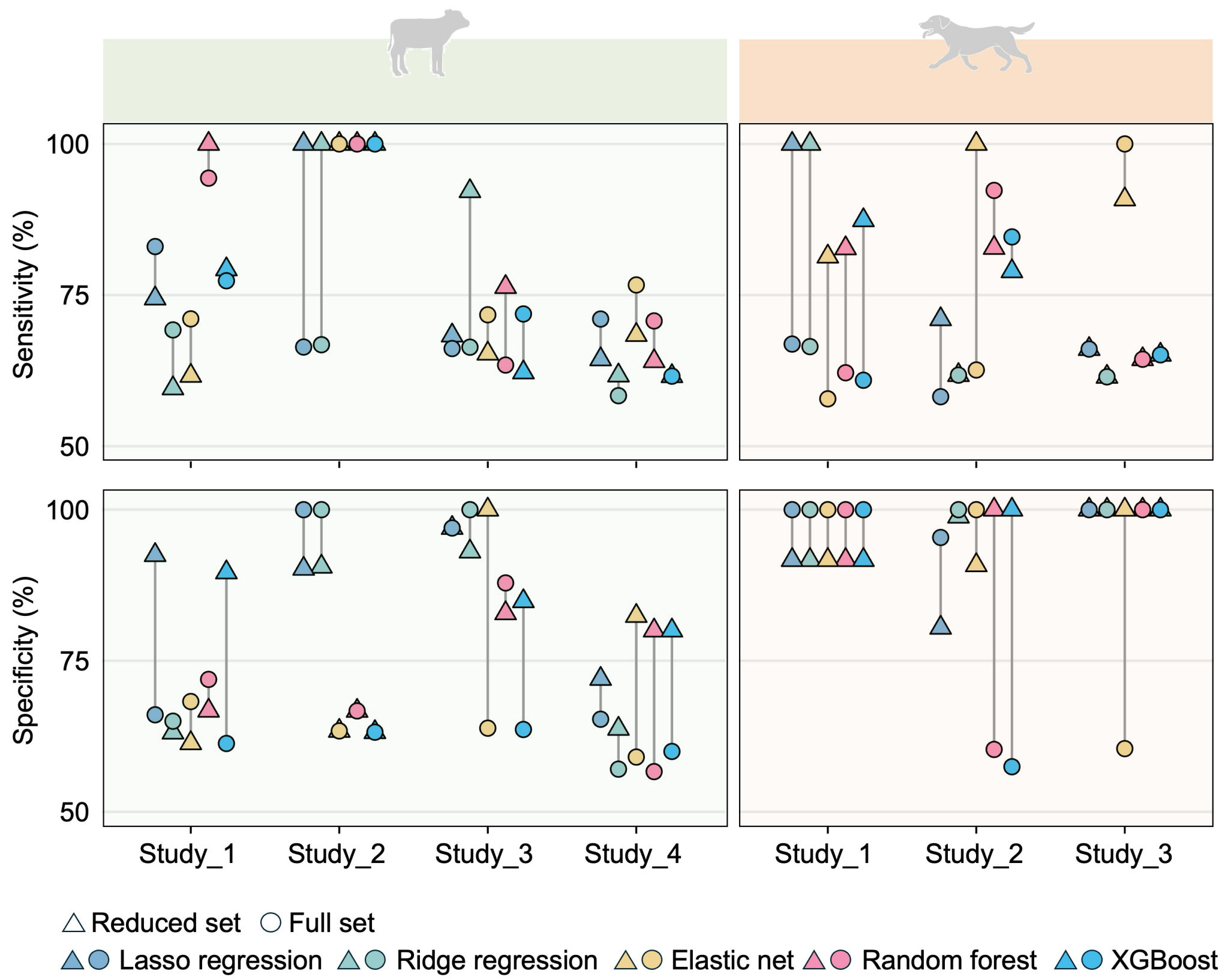

Supplementary Figure 4. Sensitivity and specificity of within-host cross-study classification under the LODO nested cross-validation framework. Sensitivity (top) and specificity (bottom) of five machine learning classifiers (LASSO, Ridge, Elastic Net, Random Forest, XGBoost) trained on signature genera (reduced set, triangles) or all genera (full set, circles) and evaluated on each held-out study for bovine diarrhea (left, 4 studies) and canine IBD (right, 3 studies). Vertical lines connect the reduced and full set values for each classifier within each study.

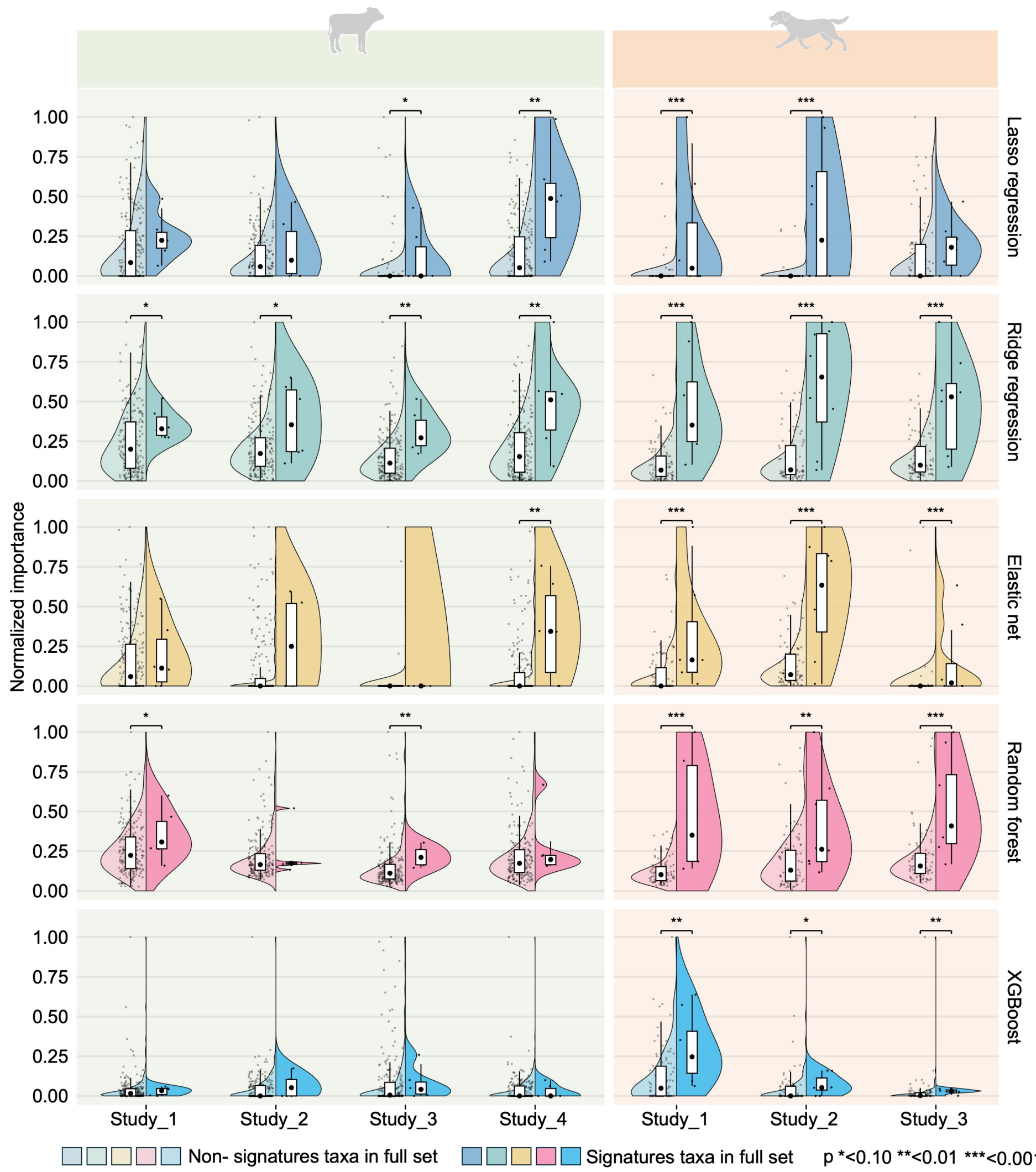

Supplementary Figure 5. Feature importance of signature versus non-signature genera across classifiers under the LODO nested cross-validation framework. Normalized feature importance distributions for signature genera (darker shades) versus non-signature genera in the full set (lighter shades) across five classifiers (LASSO, Ridge, Elastic Net, Random Forest, XGBoost) and each held-out study for bovine diarrhea (left, 4 studies) and canine IBD (right, 3 studies). Violin plots with embedded boxplots show the distribution of per-genus importance scores across cross-validation repeats; individual genus values are overlaid as points. FDR-corrected comparisons between signature and non-signature genera: *$p < 0.10$, **$p < 0.01$, ***$p < 0.001$. In all boxplots, the center line indicates the median, box limits represent the 25th and 75th percentiles, and whiskers extend to 1.5× the interquartile range.

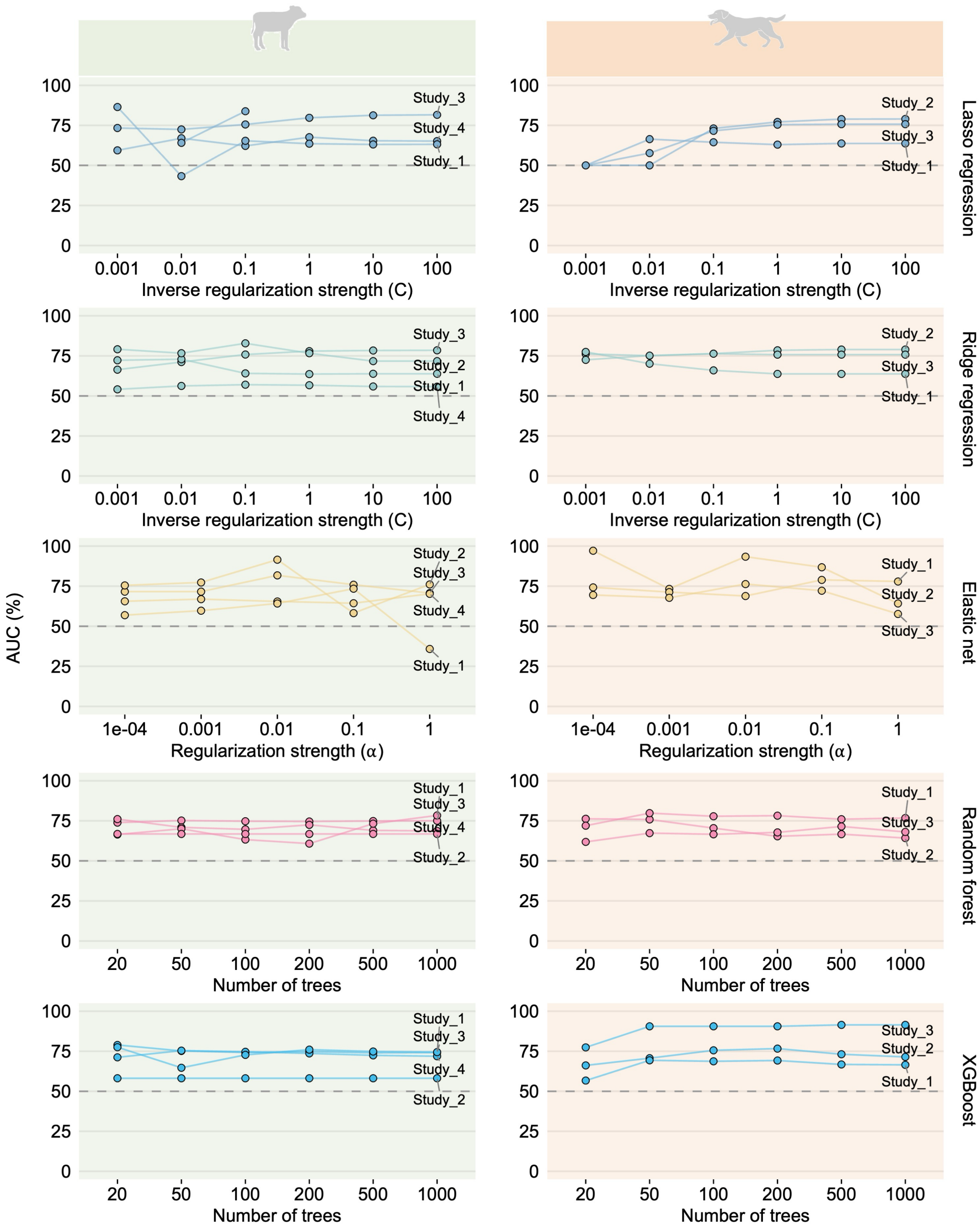

Supplementary Figure 6. Hyperparameter sensitivity analysis for five classifiers under the LODO nested cross-validation framework. Classification performance (AUC) across a range of hyperparameter values for each held-out study in bovine diarrhea (left, 4 studies) and canine IBD (right, 3 studies), evaluated using signature genera. Rows show results for LASSO and Ridge regression (inverse regularization strength C: 0.001–100), Elastic Net (regularization strength α: 1×10^{-4}–1), Random Forest, and XGBoost (number of trees: 20–1000). Each point represents the median AUC for one held-out study at a given hyperparameter value; lines connect values across the sweep for each study.

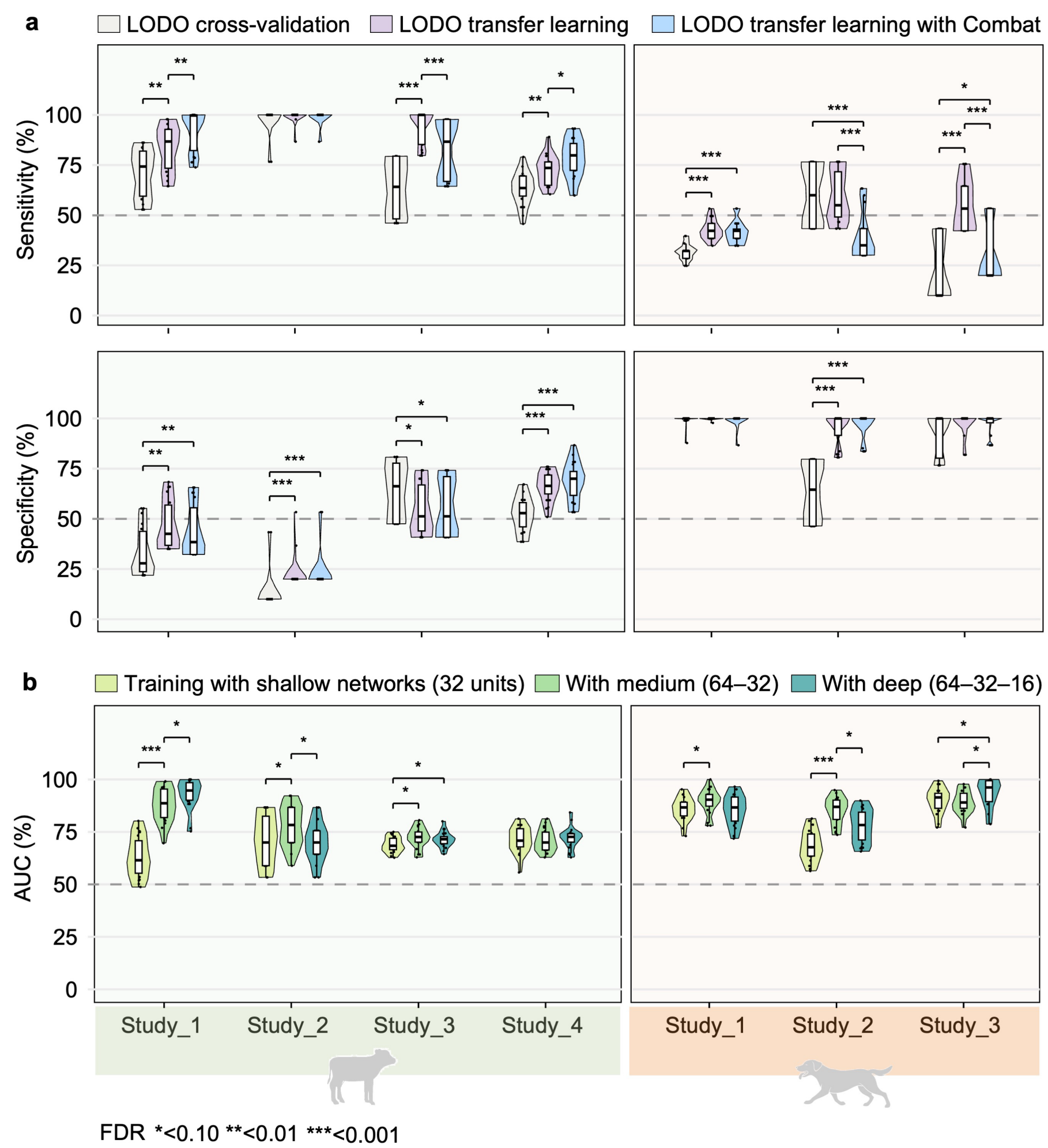

Supplementary Figure 7. Sensitivity and specificity of transfer learning strategies, and effect of neural network architecture on classification performance. (a) Sensitivity (top) and specificity (bottom) of three cross-study transfer strategies (LODO cross-validation, LODO transfer learning, and LODO transfer learning with ComBat) across each held-out study for bovine diarrhea (left, 4 studies) and canine IBD (right, 3 studies). Distributions across 20 repeated runs are shown as violin plots with embedded boxplots, with individual repeat values overlaid as points. FDR-corrected pairwise comparisons: *$p < 0.10$, **$p < 0.01$, ***$p < 0.001$. (b) Classification performance (AUC) of three neural network architectures (shallow: 32 units; medium: 64–32 units; deep: 64–32–16 units) evaluated under the LODO transfer learning framework for bovine diarrhea (left) and canine IBD (right). Distributions across 20 repeated runs are shown as violin plots with embedded boxplots, with individual repeat values overlaid as points. FDR-corrected pairwise comparisons: *$p < 0.10$, **$p < 0.01$, ***$p < 0.001$. In all boxplots, the center line indicates the median, box limits represent the 25th and 75th percentiles, and whiskers extend to 1.5× the interquartile range.

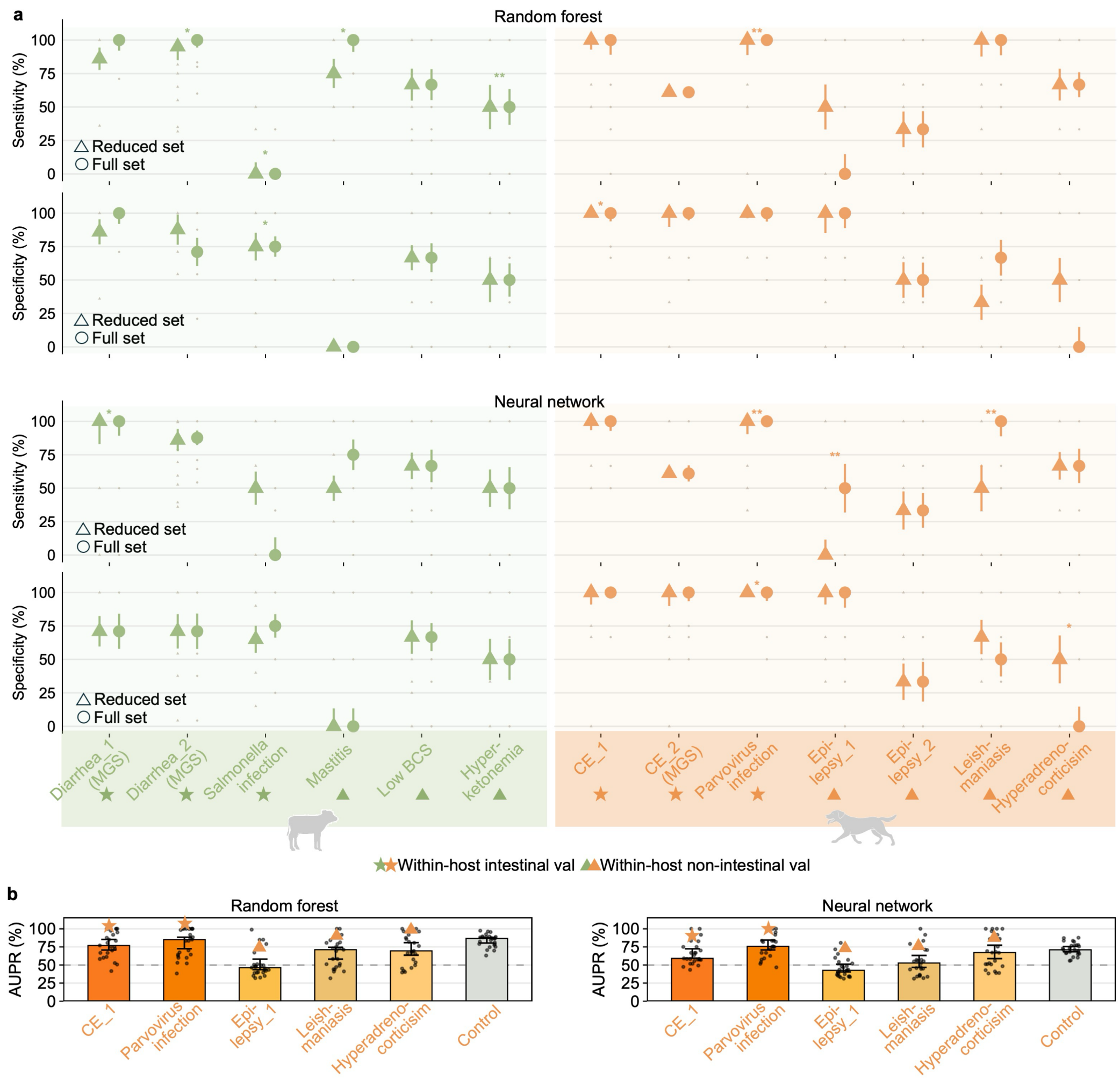

Supplementary Figure 8. Sensitivity, specificity, and AUPR of within-host cross-disease classification. (a) Sensitivity (top) and specificity (bottom) of Random Forest (top half) and Neural Network (bottom half) models using bovine diarrhea-associated (left) or canine IBD-associated (right) signature genera (reduced set, triangles) or all genera (full set, circles), evaluated across within-host intestinal (stars) and non-intestinal (filled triangles) validation datasets via 5 repeats × 5-fold cross-validation. Error bars indicate 95% confidence intervals. FDR-corrected significance: **p* < 0.10, ***p* < 0.01, ****p* < 0.001. (b) Area under the precision-recall curve (AUPR) per class for multi-class disease prediction in canine, using Random Forest (left) and Neural Network (right). Bar height indicates the median AUPR across 5-fold stratified cross-validation × 5 repeats; individual fold values are overlaid as points. Error bars indicate 95% confidence intervals. Sample sizes (n biologically independent samples) for within-host validation datasets. Bovine intestinal: Diarrhea_1 (n = 19; 9 controls, 10 cases), Diarrhea_2 (n = 42; 14 controls, 28 cases), *Salmonella* infection (n = 48; 30 controls, 18 cases). Bovine non-intestinal: Mastitis (n = 30; 10 controls, 20 cases), Low body condition score (BCS) (n = 32; 16 controls, 16 cases), Hyperketonemia (n = 21; 11 controls, 10 cases). Canine intestinal: *Parvovirus* infection (n = 26; 14 controls, 12 cases), CE_1 (n = 27; 16 controls, 11 cases), CE_2 (n = 22; 17 controls, 5 cases). Canine non-intestinal: Epilepsy_1 (n = 22; 12 controls, 10 cases), Epilepsy_2 (n = 28; 14 controls, 14 cases), Leishmaniasis (n = 26; 14 controls, 12 cases), Hyperadrenocorticism (n = 24; 9 controls, 15 cases).

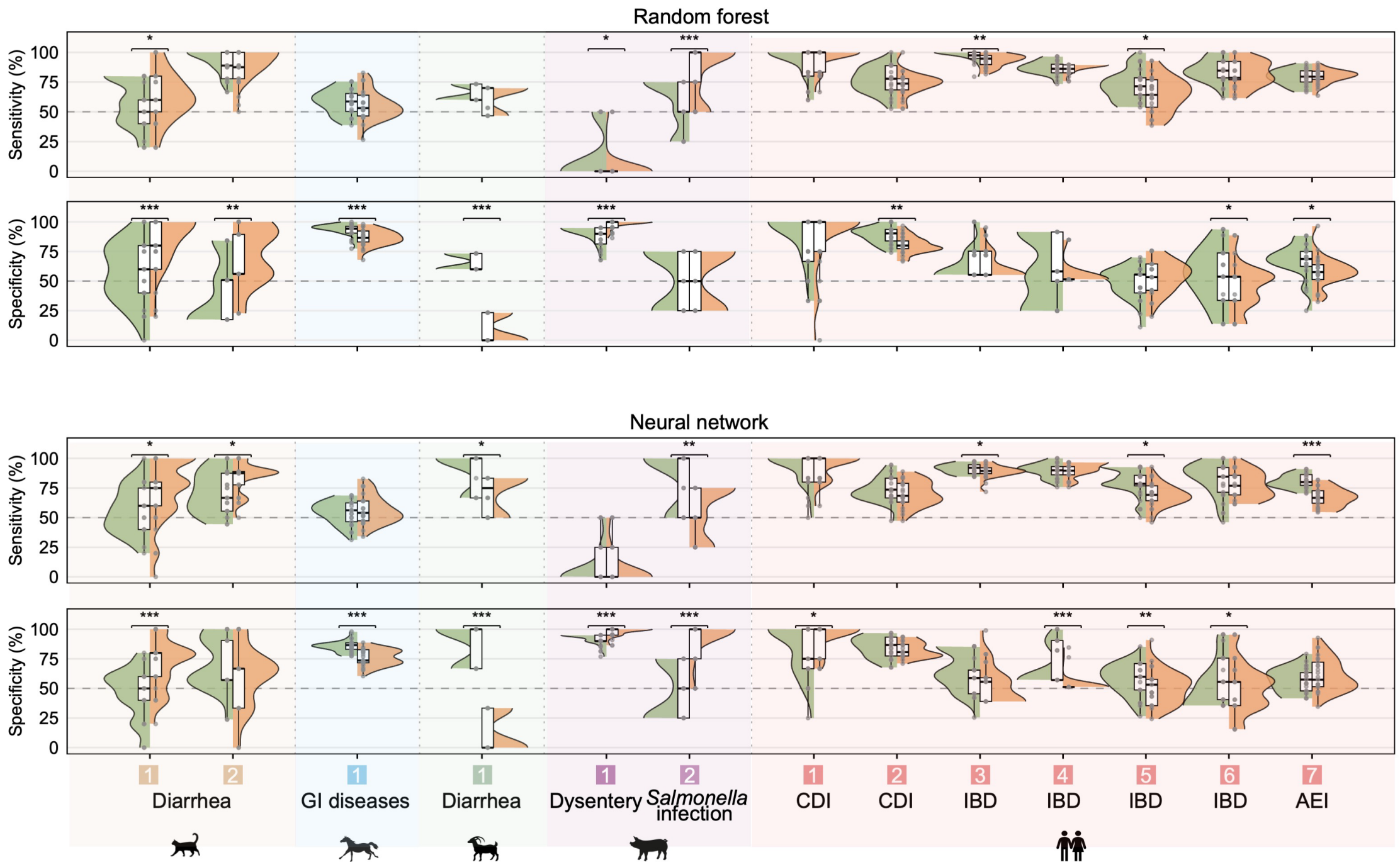

Supplementary Figure 9. Sensitivity and specificity of cross-host intestinal classification using bovine diarrhea and canine IBD signatures. Sensitivity (top) and specificity (bottom) of Random Forest (top half) and Neural Network (bottom half) models trained using bovine diarrhea-associated (green) or canine IBD-associated (orange) signature genera, evaluated across canine, equine, caprine, swine, and human intestinal disease datasets via 5 repeats × 5-fold cross-validation. Distributions are shown as violin plots with embedded boxplots, with individual repeat values overlaid as points. FDR-corrected pairwise comparisons between bovine and canine signatures: **p* < 0.10, ***p* < 0.01, ****p* < 0.001. In all boxplots, the center line indicates the median, box limits represent the 25th and 75th percentiles, and whiskers extend to 1.5× the interquartile range. Sample sizes (n biologically independent samples) for animal cross-host validation datasets: feline diarrhea (n = 48; 24 controls, 24 cases and n = 58; 15 controls, 43 cases), swine *Salmonella* infection (n = 15; 7 controls, 8 cases), swine dysentery (n = 60; 53 controls, 7 cases), caprine diarrhea (n = 10; 5 controls, 5 cases), equine gastrointestinal (GI) disease (n = 342; 263 controls, 79 cases).

Supplementary Table 1. Leave-one-dataset-out (LODO) signature stability across folds.

Host	fold_test	Number of signatures	Number of full set	Overlap with stable signatures	Number of stable signatures	Signature retention %
Bovine (calf) diarrhea	Study_3	35	214	10	12	10/12 ×100=83
Bovine (calf) diarrhea	Study_4	28	214	10	12	10/12 ×100=83
Bovine (calf) diarrhea	Study_1	20	214	8	12	8/12 ×100=67
Bovine (calf) diarrhea	Study_2	39	214	12	12	12/12 ×100=100
Canine diarrhea	Study_1	13	98	8	8	8/8 ×100=100
Canine diarrhea	Study_2	13	98	6	8	6/8 ×100=75
Canine diarrhea	Study_3	13	98	8	8	8/8 ×100=100

Supplementary Table 2. Per-fold pooled Hedges' g and FDR-adjusted p-values for stable signatures.

Host	Stable signatures	Study_1 pooled Hedges' g	Study_1 FDR	Study_2 pooled Hedges' g	Study_2 FDR	Study_3 pooled Hedges' g	Study_3 FDR	Study_4 pooled Hedges' g	Study_4 FDR
Bovine (calf) diarrhea	[Eubacterium]_nodatum_group	-0.26	0.01	-0.25	0.01	-0.20	0.02	-0.28	0.01
Bovine (calf) diarrhea	[Ruminococcus]_gauvreauii_group	0.38	0.01	0.65	0.01	0.64	0.00	0.86	0.00
Bovine (calf) diarrhea	[Ruminococcus]_torques_group	0.12	0.04	0.31	0.01	0.25	0.03	0.34	0.01
Bovine (calf) diarrhea	Bacteroides	-0.31	0.02	-0.29	0.01	-0.49	0.00	-0.37	0.01
Bovine (calf) diarrhea	Barnesiella	-0.27	0.01	-0.50	0.01	-0.62	0.01	-0.58	0.01
Bovine (calf) diarrhea	Butyricimonas	-0.31	0.04	-0.45	0.01	-0.71	0.00	-0.34	0.04
Bovine (calf) diarrhea	Clostridia_vadinBB60_group	-0.22	0.02	-0.54	0.02	-0.71	0.01	-0.65	0.01
Bovine (calf) diarrhea	Muribaculaceae	-0.41	0.00	-0.81	0.01	-1.01	0.01	-1.02	0.01
Bovine (calf) diarrhea	Olsenella	0.18	0.03	0.30	0.01	0.40	0.01	0.42	0.00
Bovine (calf) diarrhea	Oscillospira	-0.53	0.01	-0.66	0.01	-1.04	0.01	-1.02	0.01
Bovine (calf) diarrhea	Rikenellaceae_RC9_gut_group	-0.49	0.01	-1.17	0.01	-1.63	0.01	-1.37	0.02
Bovine (calf) diarrhea	Ruminococcus	-0.12	0.04	-0.20	0.01	-0.30	0.01	-0.28	0.01
Canine IBD	Allobaculum	-0.52	0.00	-0.34	0.02	-0.60	0.00		
Canine IBD	Bacteroides	-0.32	0.01	-0.12	0.06	-0.29	0.01		
Canine IBD	Clostridium_sensu_stricto_1	0.95	0.00	0.50	0.01	0.71	0.01		

Canine IBD	Megamonas	-1.06	0.00	-0.46	0.02	-0.70	0.02
Canine IBD	Peptoclostridium	-0.78	0.01	-0.24	0.03	-0.74	0.02
Canine IBD	Prevotella_9	-0.48	0.00	-0.88	0.00	-0.60	0.01
Canine IBD	Streptococcus	0.66	0.00	0.68	0.00	0.71	0.00
Canine IBD	Terrisporobacter	0.52	0.00	0.37	0.02	0.58	0.00

Supplementary Table 3. Number of retained stable signatures in each cross-host intestinal disease validation dataset.

Host	Dataset	Number of bovine diarrhea-derived signatures	Number of canine diarrhea-derived signatures
Feline	Diarrhea_1	11	8
Feline	Diarrhea_2	10	8
Swine	Dysentery	4	4
Swine	Salmonella infection	8	4
Equine	GI diseases	10	6
Caprine	Diarrhea	6	3
Human	Youngster 2014 CDI	7	4
Human	Schubert 2014 CDI	7	5
Human	Morgan 2012 IBD	6	5
Human	Gevers 2014 IBD	7	5
Human	Willing 2010 IBD	7	4
Human	Papa 2012 IBD	6	3
Human	Singh 2015 AEI	7	5
