# Supplementary material for: Toward disease-associated fecal microbiome signatures for domestic mammals
Source: Commun Biol. 2026 Jul 14;9:1474. doi: 10.1038/s42003-026-10647-5 (PMC13737101; doi:10.1038/s42003-026-10647-5)
Supplement: Supplementary file 3 [file 42003_2026_10647_MOESM3_ESM.pdf]

## Description of Additional Supplementary files

File name: Supplementary Data 1

Description: Studies with publicly available sequencing data from seven companion and economically important mammals, related to specific health events under a case-control design (as of February 2026).

File name: Supplementary Data 2

Description: Detailed information for all mammalian studies used in this project.
