# Supplementary material for: Toward disease-associated fecal microbiome signatures for domestic mammals
Source: Commun Biol. 2026 Jul 14;9:1474. doi: 10.1038/s42003-026-10647-5 (PMC13737101; doi:10.1038/s42003-026-10647-5)
Supplement: Supplementary file 4 [file 42003_2026_10647_MOESM6_ESM.pdf]

nature portfolio

Corresponding author(s): Dan Lin; Jessica McArt

Last updated by author(s): Apr 6, 2026

Reporting Summary

Nature Portfolio wishes to improve the reproducibility of the work that we publish. This form provides structure for consistency and transparency in reporting. For further information on Nature Portfolio policies, see our Editorial Policies and the Editorial Policy Checklist.

Statistics

For all statistical analyses, confirm that the following items are present in the figure legend, table legend, main text, or Methods section.

n/a	Confirmed	
☐	☒	The exact sample size (*n*) for each experimental group/condition, given as a discrete number and unit of measurement
☐	☒	A statement on whether measurements were taken from distinct samples or whether the same sample was measured repeatedly
☐	☒	The statistical test(s) used AND whether they are one- or two-sided<br>*Only common tests should be described solely by name; describe more complex techniques in the Methods section.*
☐	☒	A description of all covariates tested
☐	☒	A description of any assumptions or corrections, such as tests of normality and adjustment for multiple comparisons
☐	☒	A full description of the statistical parameters including central tendency (e.g. means) or other basic estimates (e.g. regression coefficient) AND variation (e.g. standard deviation) or associated estimates of uncertainty (e.g. confidence intervals)
☐	☒	For null hypothesis testing, the test statistic (e.g. *F*, *t*, *r*) with confidence intervals, effect sizes, degrees of freedom and *P* value noted<br>*Give P values as exact values whenever suitable.*
☒	☐	For Bayesian analysis, information on the choice of priors and Markov chain Monte Carlo settings
☒	☐	For hierarchical and complex designs, identification of the appropriate level for tests and full reporting of outcomes
☐	☒	Estimates of effect sizes (e.g. Cohen's *d*, Pearson's *r*), indicating how they were calculated

Our web collection on statistics for biologists contains articles on many of the points above.

Software and code

Policy information about availability of computer code

Data collection

No software was used for data collection by the authors, as the study utilized publicly available datasets. Raw sequencing data were downloaded from their respective public repositories (e.g., NCBI SRA or EBI), as described in the Data availability.

Data analysis

Microbiome Pipeline: QIIME 2 (v2024.10) with q2-cutadapt, DADA2 (v1.26), q2-feature-classifier, and q2-taxa modules. fastp (v0.23.1) for SMG quality filtering. BWA-MEM (v0.7.17) for host read removal.

Statistical Analysis (R v4.3.2):

Compositional data transformation: zCompositions (v1.6.0) for CZM/GBM zero replacement + CLR transformation.
Meta-analysis: metafor (v4.8.0) for DerSimonian-Laird random-effects model (Hedges' g).
Differential abundance: MaAsLin2.
Diversity & multivariate analysis: vegan (v2.6.10) and picante (v1.8.2).

Machine Learning (Python v3.11):

scikit-learn (v1.6.1) for LASSO, Ridge, elastic net (SGDClassifier), Random Forest, and MLP (MLPClassifier).
XGBoost (v3.2.0) for gradient boosting.
pyComBat (v0.20) for batch correction within CV.
Reference Database: SILVA (v138); MetaPhlAn4 (mpa_vJan25_CHOCOPhlAnSGB_202503; v4.2.4).

For manuscripts utilizing custom algorithms or software that are central to the research but not yet described in published literature, software must be made available to editors and reviewers. We strongly encourage code deposition in a community repository (e.g. GitHub). See the Nature Portfolio guidelines for submitting code & software for further information.

Data

Policy information about availability of data

All manuscripts must include a data availability statement. This statement should provide the following information, where applicable:

- Accession codes, unique identifiers, or web links for publicly available datasets
- A description of any restrictions on data availability
- For clinical datasets or third party data, please ensure that the statement adheres to our policy

All data used in the article are either publicly available or have been directly obtained from the authors of the original publications, as summarized in Table S2: NCBI's Sequence Read Archive (SRA) with the accession number PRJEB25741 for Whon et al., PRJNA918869 for Li et al., PRJNA716761 for Chen et al., PRJNA1263132 for Zhang et al., PRJNA956982 for Shi et al., PRJNA1184454 for Li et al., PRJEB25138 for Muñoz-Vargas et al., PRJNA1095683 for Zhu et al., PRJNA1173238 for Saraphol et al., PRJNA905458 for Díaz-Regañón et al., PRJEB13362 for Vázquez-Baeza et al., PRJNA861113 for Doulidis et al., PRJNA474706 for Park et al., PRJNA1012802 for Kaga et al., PRJNA1167336 for Vecchiato et al., PRJNA746550 for García-Belenguer et al., PRJNA612483 for Muñana et al., PRJNA837097 for Meazzi et al., and PRJNA1150743 for Kang et al., PRJNA521510 for Argüello et al., PRJNA436893 for Wang et al., PRJNA908214 for Bai et al., SRP047088 for Suchodolski et al., PRJNA1218713 for Pérez et al., PRJNA1032075 for McAdams et al. The MicrobiomeHD dataset validated in this study is available on Zenodo: https://zenodo.org/records/840333. Source data for the figures in this study are available at: https://doi.org/10.5281/zenodo.20693754.

Research involving human participants, their data, or biological material

Policy information about studies with human participants or human data. See also policy information about sex, gender (identity/presentation), and sexual orientation and race, ethnicity and racism.

Reporting on sex and gender	Not applicable. This study does not involve human participants and is based entirely on the re-analysis of publicly available animal microbiome datasets.
Reporting on race, ethnicity, or other socially relevant groupings	Not applicable. This study does not involve human participants and is based entirely on the re-analysis of publicly available animal microbiome datasets.
Population characteristics	Not applicable. This study does not involve human participants and is based entirely on the re-analysis of publicly available animal microbiome datasets.
Recruitment	Not applicable. This study does not involve human participants and is based entirely on the re-analysis of publicly available animal microbiome datasets.
Ethics oversight	Not applicable. This study does not involve human participants and is based entirely on the re-analysis of publicly available animal microbiome datasets.

Note that full information on the approval of the study protocol must also be provided in the manuscript.

Field-specific reporting

Please select the one below that is the best fit for your research. If you are not sure, read the appropriate sections before making your selection.

☒ Life sciences ☐ Behavioural & social sciences ☐ Ecological, evolutionary & environmental sciences

For a reference copy of the document with all sections, see nature.com/documents/nr-reporting-summary-flat.pdf

Life sciences study design

All studies must disclose on these points even when the disclosure is negative.

Sample size	A total of 4,501 samples were initially identified from 35 studies across public databases (NCBI SRA and ENA). After applying strict inclusion/exclusion criteria, the final dataset comprised 803 controls and 618 cases from 26 studies. Sample sizes were determined by the availability of all existing high-quality public data that met our rigorous metadata and study design requirements as of February 2026.
Data exclusions	Studies were excluded if they lacked a clear case-control design, had fewer than 10 samples, or had missing metadata. Individual samples were excluded if they were: (1) undergoing treatment or in recovery, (2) involved antibiotic use, or (3) were not fecal samples. A detailed exclusion scheme is provided in Supplementary Data 1 and 2.
Replication	Findings were validated across multiple independent studies (4 independent 16S studies each for bovine diarrhea and canine IBD) to ensure the robustness of the meta-analysis. Generalizability was further tested using 3 validation scenarios: within-host intestinal, within-host non-intestinal, and cross-host intestinal models. All computational processes used a unified 16S and shotgun metagenomics processing pipeline.
Randomization	Not applicable as this is a meta-analysis of existing observational studies. However, we only included studies with clearly defined group allocation criteria based on disease status (sick vs. normal, infected vs. uninfected). Potential biases were controlled using batch effect correction and including host age and sex as covariates.

Blinding	Not applicable. The study relied on pre-existing group assignments from publicly available datasets. Data processing and filtering were performed based on objective, pre-established criteria to minimize bias.

Reporting for specific materials, systems and methods

We require information from authors about some types of materials, experimental systems and methods used in many studies. Here, indicate whether each material, system or method listed is relevant to your study. If you are not sure if a list item applies to your research, read the appropriate section before selecting a response.

Materials & experimental systems

n/a	Involved in the study
☒	☐ Antibodies
☒	☐ Eukaryotic cell lines
☒	☐ Palaeontology and archaeology
☐	☒ Animals and other organisms
☒	☐ Clinical data
☒	☐ Dual use research of concern
☒	☐ Plants

Methods

n/a	Involved in the study
☒	☐ ChIP-seq
☒	☐ Flow cytometry
☒	☐ MRI-based neuroimaging

Animals and other research organisms

Policy information about studies involving animals; ARRIVE guidelines recommended for reporting animal research, and Sex and Gender in Research

Laboratory animals	Not applicable. This study does not involve new animal experiments performed by the authors. It is a re-analysis of publicly available 16S rRNA and SMG sequencing data from domestic mammals (bovine and canine) retrieved from public repositories (NCBI SRA/ENA).
Wild animals	Not applicable.
Reporting on sex	Not applicable.
Field-collected samples	Not applicable.
Ethics oversight	Not applicable. Ethical approval for the original data collection was obtained by the primary investigators of the source studies as described in the cited literature. No new animal samples were collected for this study.

Note that full information on the approval of the study protocol must also be provided in the manuscript.

Plants

Seed stocks	Not applicable.
Novel plant genotypes	Not applicable.
Authentication	Not applicable.
